# Sulfoxide-Directed Metal-Free *ortho*-Propargylation of Aromatics and Heteroaromatics

**DOI:** 10.1002/chem.201406424

**Published:** 2015-03-06

**Authors:** Andrew J Eberhart, Harry J Shrives, Estela Álvarez, Amandine Carrër, Yuntong Zhang, David J Procter

**Affiliations:** [a] School of Chemistry, University of Manchester Oxford Road, Manchester M13 9PL (UK), Fax: (+44) 161-275-4939 E-mail: david.j.procter@manchester.ac.uk Homepage: http://people.man.ac.uk/∼mbdssdp2/

**Keywords:** alkynes, cross-coupling, metal-free, Pummerer, sulfoxide

## Abstract

A sulfoxide-directed, metal-free *ortho*-propargylation of aromatics and heteroaromatics exploits intermolecular delivery of a propargyl nucleophile to sulfur followed by an intramolecular relay to carbon. The operationally simple cross-coupling procedure is general, regiospecific with regard to the propargyl nucleophile, and shows complete selectivity for products of *ortho*-propargylation over allenylation. The use of secondary propargyl silanes allows metal-free *ortho*-coupling to form carbon–carbon bonds between aromatic and heteroaromatic rings and secondary propargylic centres. The ‘safety-catch’ nature of the sulfoxide directing group is illustrated in a selective, iterative double cross-coupling process. The products of propargylation are versatile intermediates and they have been readily converted into substituted benzothiophenes.

## Introduction

Selective carbon–carbon bond formation to aromatic and heteroaromatic systems is an important synthetic objective because the resulting structural motifs are found in many pharmaceuticals, agrochemicals and functional materials. Such transformations are currently achieved by using late-transition-metal-mediated couplings, however, issues with the cost and future supply of such metals and the metal contamination of products makes the development of metal-free coupling processes of great importance.

In particular, coupling products arising from the propargylation[1] of aromatic and heteroaromatic systems are of high value because they are versatile synthetic intermediates of relevance to the synthesis of carbo- and heterocycles.[1] Unfortunately, the direct propargylation of aromatics is often challenging and can lead to mixtures of propargyl and allenyl products. Although metal-catalysed couplings are possible,[2] many methods rely on electrophilic Friedel–Crafts-type processes that can require stoichiometric metal reagents.[3] In recent years, a new strategy has emerged in which sulfoxide substituents have been exploited as activating groups in nucleophilic alkylations of electron-rich heteroaromatics[4] that proceed through Pummerer-type reactions.[5] In particular, Yorimitsu[6a–e] and Maulide[6f–i] have recently employed Pummerer reactions in approaches to targets such as benzofurans and α-aryl-β-ketoesters/α-arylamides, and we have described the use of an interrupted Pummerer approach for the allylation of aromatic and heteroaromatic rings.[7]

**Scheme 1:**
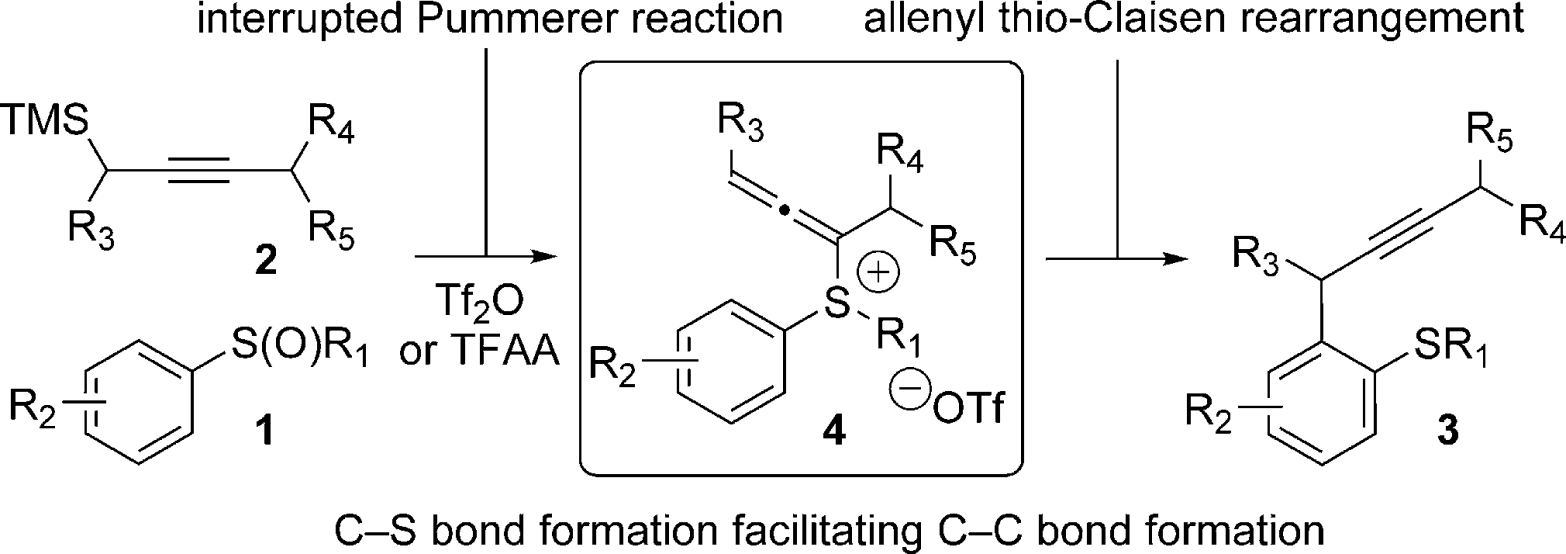
Sulfoxide-directed, metal-free *ortho*-propargylation of aromatic and heteroaromatics. TMS=trimethylsilyl, Tf=1,1,1-trifluoromethylsulfonyl.

Herein, we report in full our development of a sulfoxide-directed *ortho*-propargylation of aromatics and heteroaromatics that proceeds by a new interrupted Pummerer-allenyl thio-Claisen rearrangement[8] sequence involving allenyl sulfonium salts **4** (Scheme 1).[8j] The operationally simple, metal-free procedure is general, regiospecific with regard to the propargyl nucleophile, and shows complete selectivity for products of propargylation over allenylation.[9]

## Results and Discussion

Realising the value of a cross-coupling process that would allow propargyl groups to be selectively introduced to aromatic and heteroaromatic rings under metal-free coupling conditions, we sought to develop such a process by exploiting a strategy in which intermolecular delivery of a carbon nucleophile to sulfur would be followed by an intramolecular relay to carbon (Scheme 1). We began by investigating the reaction of diphenyl sulfoxide **1 a** with propargyl silane **2 a** (Table 1). The use of Tf_2_O (trifluoromethanesulfonic anhydride) as an electrophilic activating agent delivered propargylation product **3 a**, albeit in low yield (entry 1). We next varied the reaction solvent and obtained the best result by using MeCN (entry 4).[10] The reaction was found to proceed readily at room temperature in 36 h (entry 5) or in 1 h when the reaction temperature was increased to 60 °C (entry 6). Addition of base led to a significant improvement in yield, and **3 a** was isolated in 99 % yield (entry 8). The base prevents products of propargylation from undergoing acid-mediated cyclisation.

**Table 1:** Optimization of the sulfoxide-directed, metal-free *ortho*-propargylation^[a]^

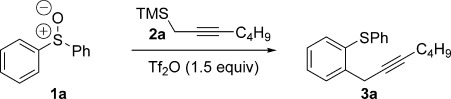					
Entry	Solvent	[h]	[°C]	Base	Yield3[%]
1	CH_2_Cl_2_	18	50	–	35
2	CHCl_3_	18	50	–	28
3	toluene	18	50	–	27
4	MeCN	18	50	–	63
5	MeCN	36	RT	–	72
6	MeCN	1	60	–	73
7^[b]^	MeCN	18	60	pyridine	16
8^[b]^	MeCN	18	60	2,6-lutidine	99^[c]^
9^[b]^	MeCN	18	60	2,6-DTBP	99

[a] Yield determined by ^1^H NMR spectroscopic analysis. [b] Base (2.5 equiv) added; [c] Isolated yield; 2,6-DTBP=2,6-di-*tert*-butylpyridine

Having optimised the reaction conditions, we next investigated the scope of the process with regard to the aromatic sulfoxide. Pleasingly, the *ortho*-propargylation reaction worked well with readily available, simple alkyl aryl sulfoxides **1 b**–**1 aa** to give the corresponding products **3 b**–**3 aa**, containing pharmaceutically relevant alkylsulfanyl groups (Table 2). Methyl phenyl sulfoxide was easily converted into **3 b** in excellent yield on a 1 gram scale. Surprisingly, the formation of classical Pummerer products was not observed, even in substrates containing electron-withdrawing alkyl chains on sulfur (e.g., formation of **3 d**, **3 e**, **3 f** and **3 aa**; see below). Attractively, the procedure also tolerates the synthetically important perfluorinated alkyl chain[11] in **3 f** and the medicinally relevant trifluoromethyl sulfide group in **3 g**.[12] The reaction also shows excellent generality with respect to ring substituents: neutral, electron-rich and electron-deficient benzene rings are propargylated in high yields (**3 h**–**z**) with no significant changes in the overall reaction efficiency observed when substitution position was varied (**3 h–p**; 71–93 %). Even sterically hindered *ortho*-substituted substrates underwent propargylation to give **3 l** and **3 t** in 85 and 96 % yield, respectively. The reaction exhibits excellent functional group tolerance: Substrates containing halogens (**3 n**–**r**), nitriles (**3 u**), nitro (**3 w**) and protected amines (**3 v**)[13] were all readily propargylated in good to excellent yields. Furthermore, substrates bearing ester, amide and acid groups underwent successful propargylation to give **3 x**–**z**. Importantly, a substrate known to undergo classical Pummerer chemistry,[14] gave **3 aa** in 89 % yield when exposed to Tf_2_O in the presence of **2 a** (see below).

**Table 2:** Sulfoxide-directed metal-free cross-couplings of aromatic substrates

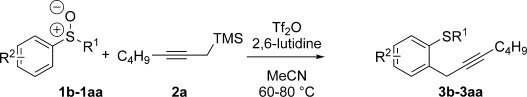		
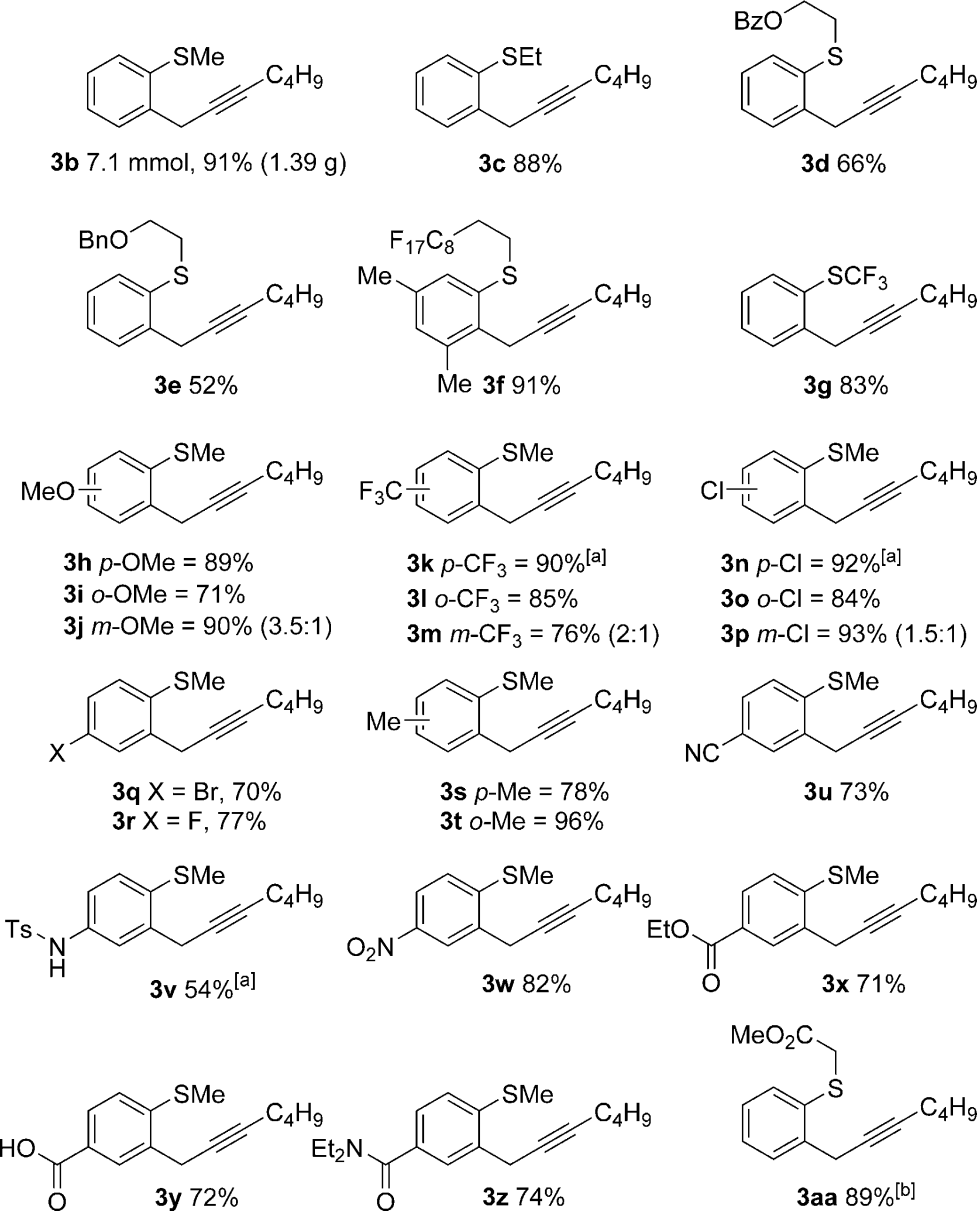		

[a] 2,6-DTBP was used as base; [b] No base.

Pleasingly, the *ortho*-propargylation reaction also works well with some readily available heteroaryl sulfoxides **1 ab**–**ak** (Table 3). Thiophene and furan substrates underwent smooth metal-free coupling provided that milder activation conditions employing trifluoroacetic anhydride (TFAA) and lower temperatures were employed for the more reactive heteroaryl sulfoxides (cf. arylsulfoxides; see Table 2). The optimal temperature in each case depended on the nature and position of the sulfoxide directing group (from −78 °C to RT). The propargylation was found to tolerate the presence of organylsulfanyl groups (**3 ak** and **3 ah**) and halogens (**3 ag**), thus suggesting further elaboration of the products by a second metal-free propargylation (after a selective oxidation of SPh; see below) or by more conventional metal-mediated couplings exploiting the carbon–halogen bond. Finally, two-directional coupling of a thiophene bearing two sulfoxide directing groups gave **3 ak** in good yield (Table 3).

**Table 3:** Sulfoxide-directed metal-free cross-couplings of heteroaromatics

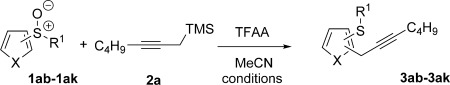		
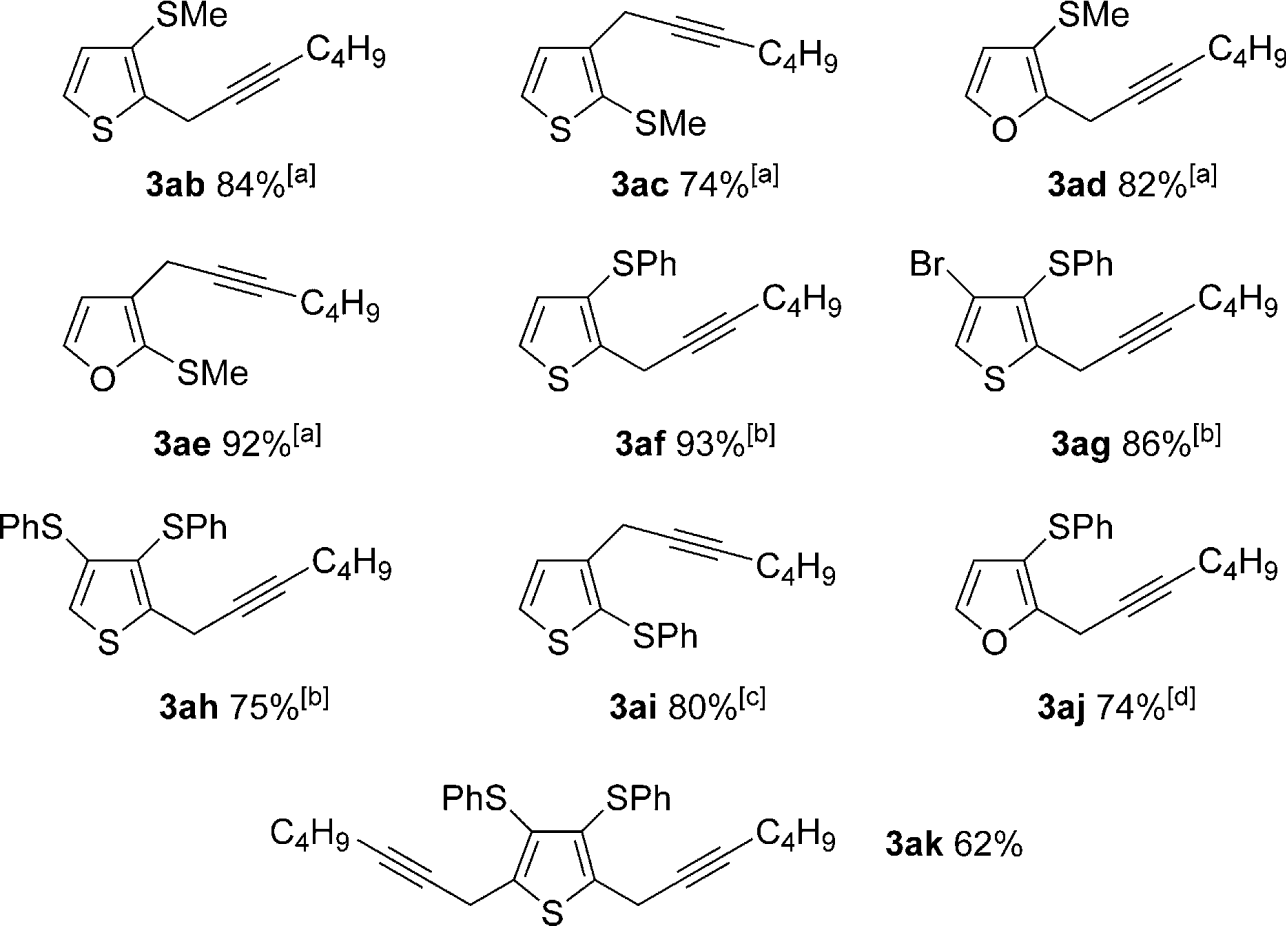		

[a] −40 °C to RT, 18 h; [b] RT, 1 h; [c] −78 °C, 2 h; [d] −20 °C to RT, 2 h.

We next explored the scope of the metal-free process with regard to the propargyl silane cross-coupling partner. Methyl phenyl sulfoxide **1 b** and/or 3-methylsulfinyl thiophene **1 ab** were exposed to propargyl silanes **2 b**–**n** under the described conditions (Table 4). In all cases, the expected products of propargylation were obtained in good to excellent yields. For example, commonly used silane **2 b** (entry 1) and the protected propargyl silane **2 d** (entries 3 and 6) produced products of selective propargylation in high yields. Sterically more demanding silanes (entries 2, 7 and 8–16) and functionalised silanes (entries 4 and 5) were also effective coupling partners. Importantly, nucleophiles having substitution at both propargylic positions **2 g**–**n** also participated in the metal-free cross-couplings to deliver products **3 as**–**ba**, in which a new carbon–carbon bond has been formed between an *ortho* sp^2^ carbon on the aromatic/heteroaromatic ring and a secondary sp^3^ propargylic centre (entries 8–16).

**Table 4:** Sulfoxide-directed metal-free cross-couplings: scope of propargyl nucleophile partner^[a]^

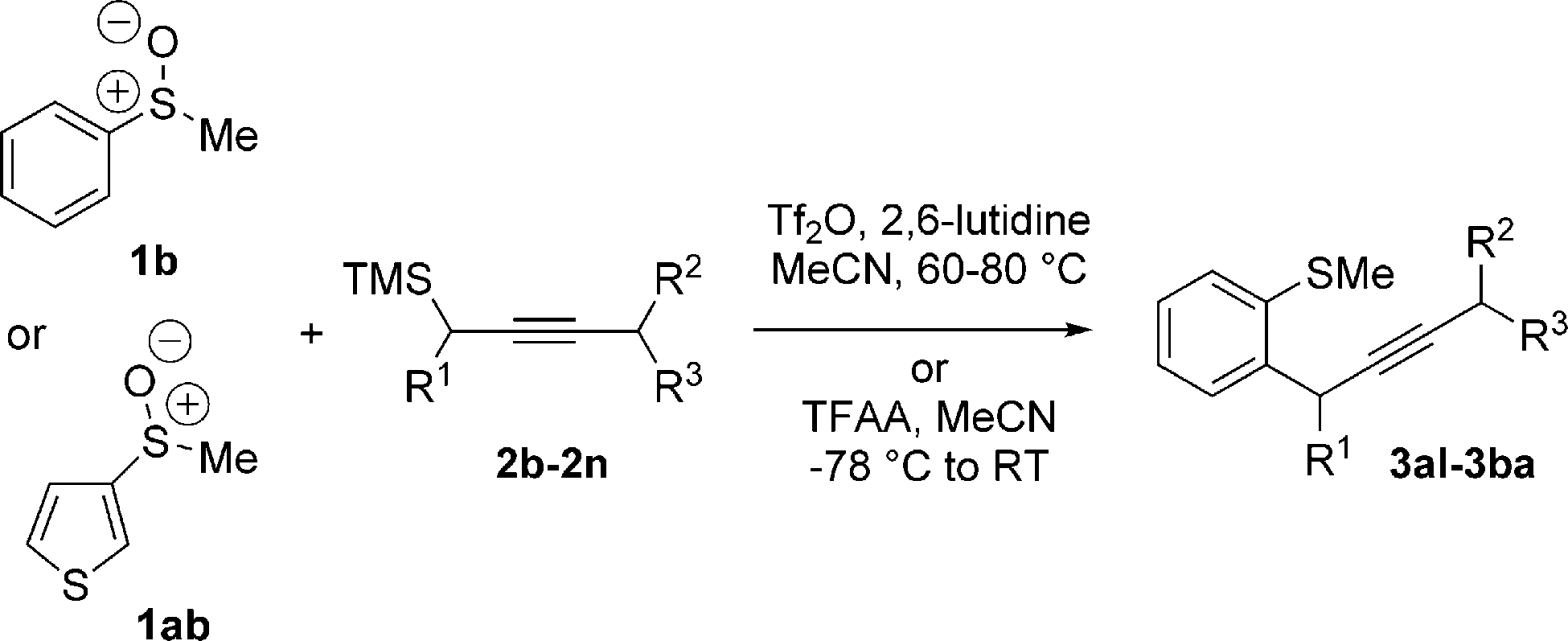							
Entry	Silane	Product	Yield [%]	Entry	Silane	Product	Yield [%]
1	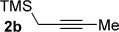	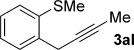	96	9	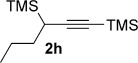	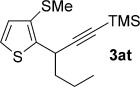	64
2	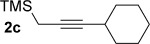	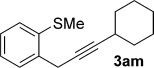	72	10	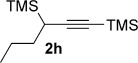	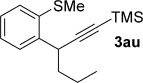	61
3	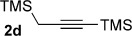	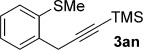	92	11	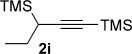	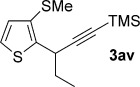	52
4	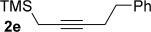	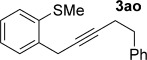	88^[b]^	12	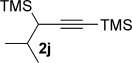	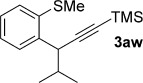	61
5	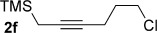	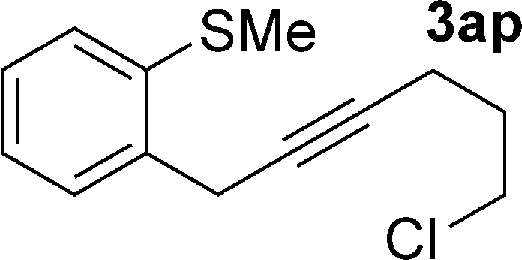	95^[b]^	13	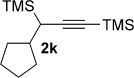	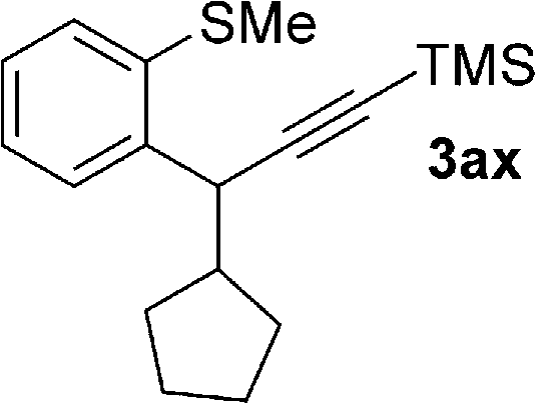	60
6	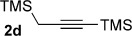	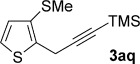	64	14	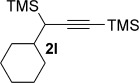	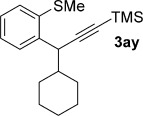	63
7	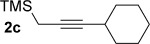	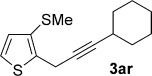	71	15	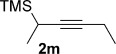	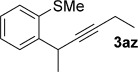	50
8	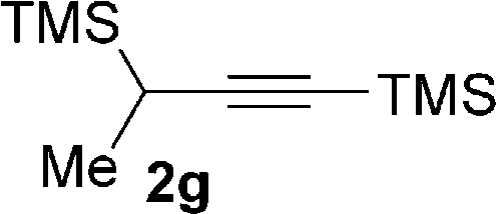	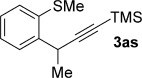	64^[b]^	16	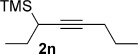	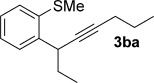	45

[a] Tf_2_O conditions used for propargylation of **1 b**; TFAA conditions used for propargylation of **1 ab**. [b] 2,6-DTBP was used as the base.

**Scheme 2:**
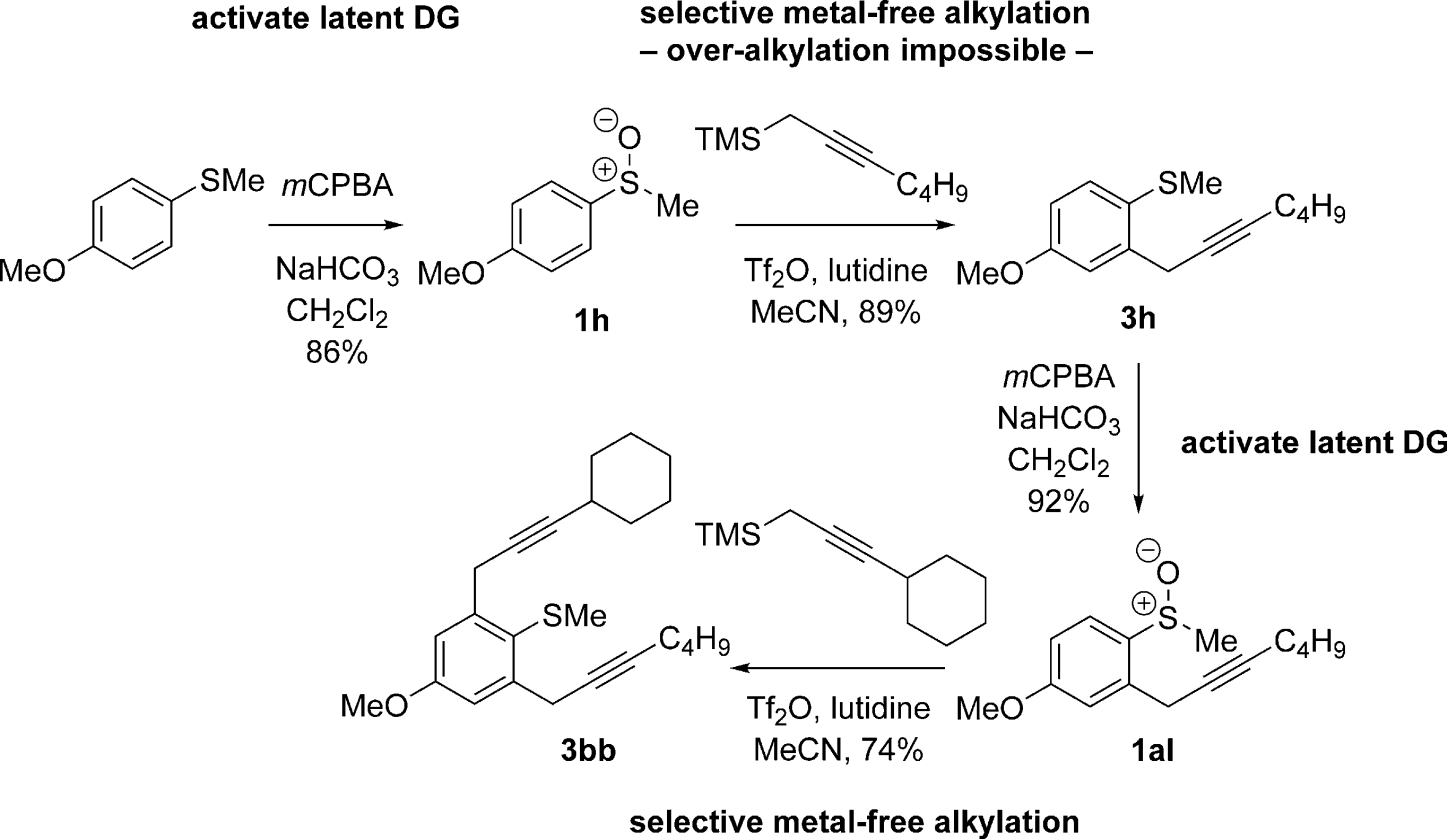
A safety-catch directing group for metal-free propargylation. DG=directing group.

Crucially, the sulfoxide in our approach acts as a ‘safety-catch’ directing group: Only upon oxidation to the sulfoxide is the substrate receptive to metal-free cross-coupling. Thus, a sulfide substituent can be carried through a synthesis before selective sulfur oxidation delivers the directing group effect precisely when and where it is required. Furthermore, over-alkylation to give mixtures is impossible because the directing group is ‘switched off’ during the metal-free coupling. Reoxidation of sulfur reactivates the directing group (releases the safety-catch) and a controlled second coupling using a different carbon nucleophile is then possible. This approach is illustrated in Scheme 2 for the selective synthesis of **3 bb** by using two metal-free cross-couplings.

**Scheme 3:**
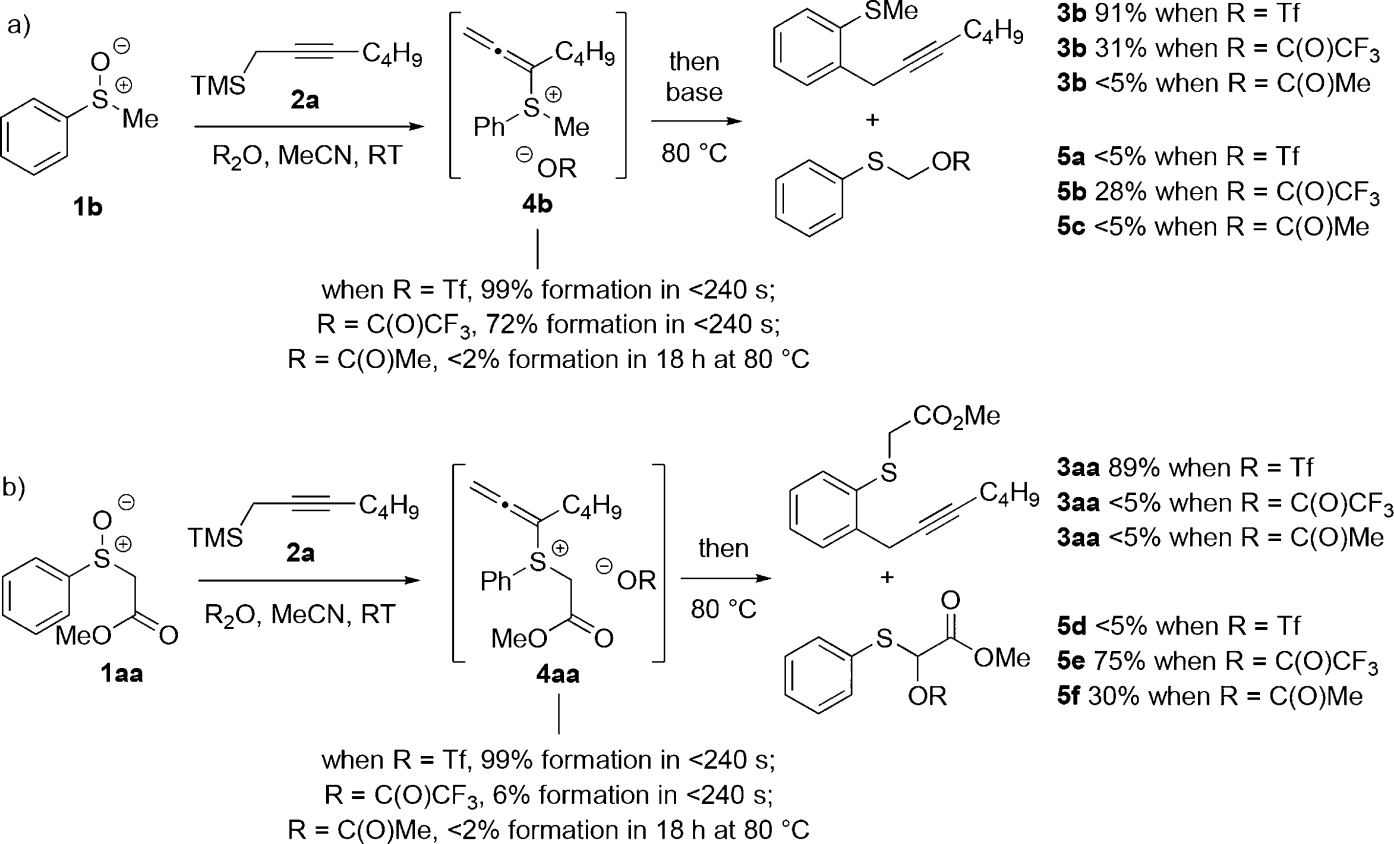
The importance of efficient sulfoxide activation in the metal-free propargylation. a) Methyl phenyl sulfoxide; b) methyl 2-(phenylsulfinyl)acetate. Yields and conversions obtained by ^1^H NMR spectroscopic analysis.

After electrophilic activation of the sulfoxide with Tf_2_O/TFAA,[15] the nucleophile reacts at sulfur in an interrupted Pummerer-type reaction,[7] followed by rearrangement during which the incoming group is passed to the aromatic ring. An alternative mechanism in which the nucleophile attacks directly at the aromatic ring with concomitant triflate expulsion followed by rearomatisation[4a,b] can be ruled out because regioisomeric products of *ortho* and *para* allenylation would result: our process provides products of propargylation, with no allenylation, with complete *ortho*-selectivity, clearly suggesting that the interrupted Pummerer pathway is operational. Furthermore, allenyl sulfonium salt **4** (Scheme 1), formed by nucleophilic addition to sulfur, can be observed when reactions are monitored by ^1^H and ^13^C NMR spectroscopy.[16, 17] The formation of **4** is surprisingly fast and outcompetes classical thionium ion formation and the Pummerer reaction. The choice of the electrophilic activator is key to the success of the interrupted Pummerer-type process. For example, in the reaction of sulfoxide **1 b** with nucleophile **2 a**, only activation with Tf_2_O leads to efficient formation of the allenylsulfonium salt intermediate **4 b** and thus to high yields of **3 b**. The use of TFAA as an activator leads to significant amounts of classical Pummerer product **5 b**, whereas the use of Ac_2_O results in no reaction (Scheme 3 a). Even sulfoxide **1 aa**, bearing acidic α-protons, underwent smooth formation of allenylsulfonium salt **4 aa** upon activation with Tf_2_O, thus delivering propargylation product **3 aa** in high yield. Attempted activation with TFAA and Ac_2_O led to inefficient allenylsulfonium salt formation and significant amounts of the classical Pummerer products **5 e** and **5 f** (Scheme 3 b).

**Scheme 4:**
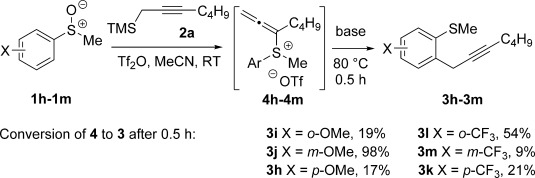
The effect of substituents and their location on the rate of rearrangement in the metal-free propargylation. Yields and conversions obtained by ^1^H NMR spectroscopic analysis.

The nature of substituents on the aryl ring, as well as their positions, affect the rate of the rearrangement of allenylsulfonium salts **4** to coupling products **3**. For example, the location of an electron-releasing ‘OMe’ substituent and an electron-withdrawing ‘CF_3_’ substituent on the benzene ring in a series of aryl sulfoxide substrates had a marked effect on the preliminary rate of conversion of allenylsulfonium salts **4** into propargylated products **3**.[18] This is particularly the case for *meta*-substitution: whereas electron-rich **4 j** underwent almost quantitative conversion into **3 j** after 30 min, electron-deficient **4 m** underwent only 10 % conversion into **3 m** after the same period. Interestingly, sterically hindered **4 l** also showed an increased rate of rearrangement to **3 l** (Scheme 4).

**Scheme 5:**
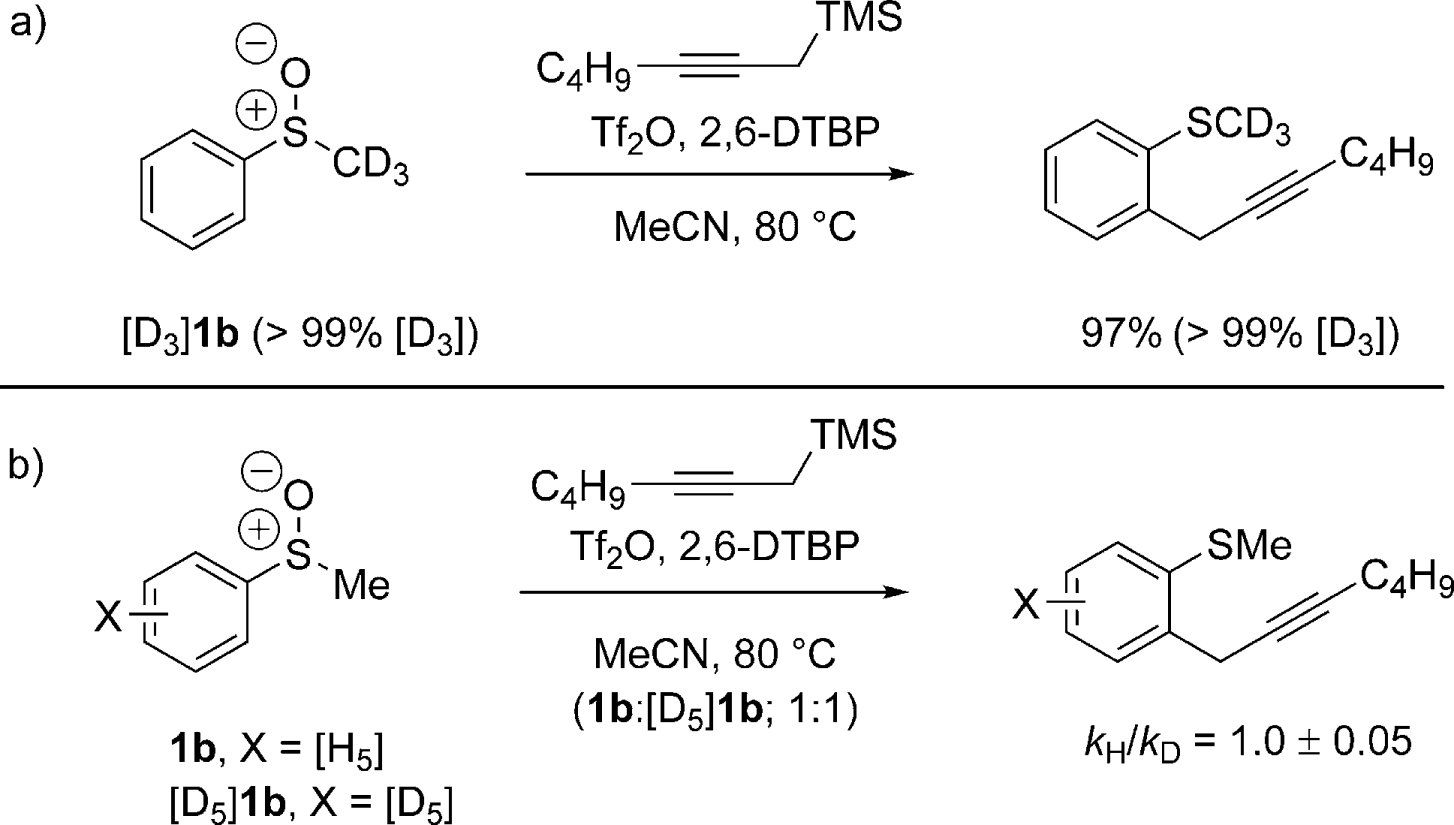
Mechanistic studies involving labelled substrates. Yields and conversions obtained by ^1^H NMR spectroscopic analysis.

In additional mechanistic investigations, labelled sulfoxide [D_3_]**1 b** (Scheme 5 a) was propargylated with no ^1^H incorporation at the methyl group, further highlighting the rapid formation of allenylsulfonium salts **4** by attack at sulfur rather than loss of a proton α- to sulfur. Furthermore, a competition experiment involving a 1:1 mixture of **1 b** and its aryl-deuterium labelled analogue [D_5_]**1 b** (Scheme 5 b) showed no kinetic isotope effect, suggesting that rearomatisation is not the rate-determining step.

**Scheme 6:**
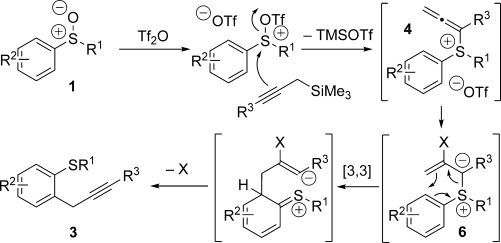
Proposed mechanism for the sulfoxide-directed, *ortho*-propargylation.

A possible mechanism for the selective *ortho*-propargylation is therefore summarised in Scheme 6. Interrupted Pummerer reactions, in which sulfoxides **1** activated by Tf_2_O (or an alternative activator) undergo nucleophilic attack by the propargyl silanes at sulfur, give allenylsulfonium salts **4** (observed by ^1^H and ^13^C NMR spectroscopy). These intermediates may then convert into ylides **6** prior to [3,3]-sigmatropic rearrangement[8, 19] and rearomatisation to yield **3**.[20] Substituent ‘X’ could be triflates,[9] although MeCN, sulfide or base cannot be ruled out.

**Scheme 7:**
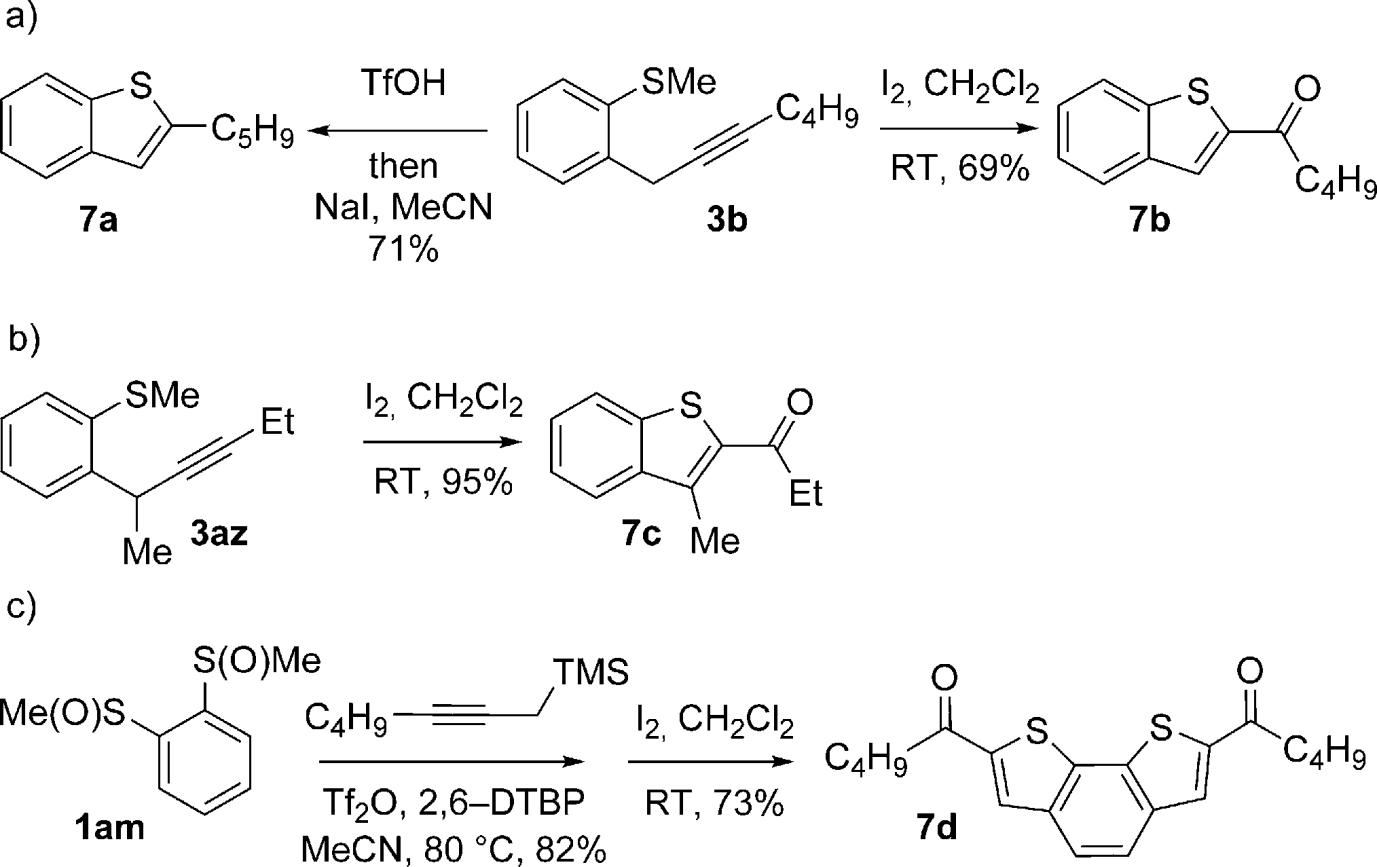
Manipulation of *ortho*-propargylation products.

The products of metal-free *ortho*-propargylation are rich in synthetic potential because the combination of the alkyne[1] and organosulfanyl groups opens up a wide range of bond-forming possibilities. In particular, recent developments have shown that substrates bearing C–S bonds are of growing utility as partners in transition-metal-catalysed cross-couplings to form C–C bonds.[6d, [7a], [21] Preliminary studies show the potential of the dual functionality in the products arising from sulfoxide-directed metal-free propargylation. For example, **3 b** was converted into benzothiophenes **7 a** and **7 b** by treatment with TfOH/NaI and I_2_,[22] respectively (Scheme 7 a). Coupling products arising from the use of nucleophilic partners having substitution at both propargylic positions also undergo heterocyclisation upon exposure to I_2_; for example, coupling product **3 az** is converted into 2,3-disubstituted benzothiophene **7 c** upon exposure to I_2_ (Scheme 7 b). Finally, double cyclisation of the adduct, formed by the two-directional propargylation of bis-sulfoxide **1 am**, completed an efficient metal-free approach to **7 d**, which contains a motif present in organic semiconductors[23] (Scheme 7 c).

## Conclusions

In summary, readily available aryl and heteroaryl sulfoxides undergo sulfoxide-directed *ortho*-selective propargylation under metal-free conditions. The cross-coupling process involves a new interrupted Pummerer/allenyl thio-Claisen rearrangement sequence. The operationally simple procedure allows propargylic carbon nucleophiles to be added *ortho* to sulfur on an aromatic or heteroaromatic ring, regiospecifically with regard to the propargyl nucleophile, and with complete selectivity for products of propargylation over allenylation. The use of coupling partners bearing substitution at both propargylic positions allows carbon–carbon bonds between aryl sp^2^ and secondary propargylic sp^3^ carbon centres to be constructed. The ‘safety-catch’ nature of the sulfoxide directing group is illustrated in a selective, iterative double cross-coupling process. The organosulfanyl group and the alkyne motif in the coupling products are versatile handles for further manipulation.

## Supporting Information

As a service to our authors and readers, this journal provides supporting information supplied by the authors. Such materials are peer reviewed and may be re-organized for online delivery, but are not copy-edited or typeset. Technical support issues arising from supporting information (other than missing files) should be addressed to the authors.

miscellaneous_information
