# Supplementary material for: Sulfoxide-Directed Metal-Free ortho-Propargylation of Aromatics and Heteroaromatics
Source: Chemistry. 2015 Mar 6;21(20):7428–34. doi: 10.1002/chem.201406424 (PMC4524421; doi:10.1002/chem.201406424)

CHEMISTRY
A European Journal

# Supporting Information

## Sulfoxide-Directed Metal-Free *ortho*-Propargylation of Aromatics and Heteroaromatics

Andrew J. Eberhart, Harry J. Shrives, Estela Álvarez, Amandine Carrër, Yuntong Zhang, and David J. Procter*[a]

chem_201406424_sm_miscellaneous_information.pdf

# Supporting Information

## Contents

General Experimental ....S2

General Procedure A Oxidation of Sulfides ....S3

General Procedure B Preparation of 1,3-*bis* propargyl silanes ....S7

General Procedure C Preparation of Propargyl Silanes ....S10

Propargylation of Heterocycles ....S12

General Procedure D: Propargylation of Aryl Sulfoxides ....S20

Iterative, selective *ortho*-propargylation of 4-methoxyphenylmethyl sulfide ....S25

Iodine Mediated Cyclization ....S26

1-(3-Methylbenzo[b]thiophen-2-yl)propan-1-one **7c** ....S26

$^{1}$H and $^{13}$C NMR ....S28

## General Experimental

All experiments were performed under an atmosphere of nitrogen, using anhydrous solvents, unless stated otherwise. THF was distilled from sodium/benzophenone and $CH_2Cl_2$ was distilled from $CaH_2$. All other solvents and reagents were purchased from commercial sources and used as supplied.

$^1$H NMR spectra were recorded on a 300, 400 or 500 MHz spectrometer. $^{13}$C NMR spectrawere recorded on a 75, 100 or 125 MHz spectrometer. All chemical shift values are reported in ppm, with coupling constants in Hz. Mass spectra were obtained using positive or negative electrospray (ES), electron ionization (EI), chemical ionization (CI) or gas chromatography-mass spectrometry methodology. Infra-red spectra were recorded as evaporated films or neat using FT/IR spectrometers.

Melting points were measured on solids as obtained after chromatography Column chromatography was carried out using 35 – 70 μ, 60Å silica gel. Routine TLC analysis was carried out on silica gel 60 F254 coated aluminium sheets of 0.2 mm thickness. Plates were viewed using a 254 nm ultraviolet lamp and developed by dipping in aqueous potassium permanganate solution.

Details for the preparation of all compounds (and associated characterization data) in table 2, compounds **2b-g**, **3al-p** and **3as** can be found in: A. J. Eberhart; D.J. Procter, *Angew. Chem. Int. Ed.*, **2013**, *52*, 4008.

For the preparation of compound **1ai** please see: A. J. Eberhart; J. E. Imbriglio, J. E.; D. J. Procter, *Org. Lett*, **2011**, 13, 5882-5885

**General Procedure A Oxidation of Sulfides**

**3-(Methylsulfinyl)thiophene 1ab**[1]

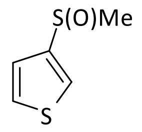


To a solution of 3-(methylthio)thiophene (1.07 g, 8.25 mmol) in dichloromethane (8.25 mL, 1 M) at 0 $^{o}$C, was added dropwise a solution of *m*-CPBA (1.99 g, 8.25 mmol) in dichloromethane. The resulting mixture was stirred at 0 $^{o}$C for 1 hour before warming to room temperature after which it was stirred for a further 1 hour. The reaction was quenched with sat. $NaHCO_3$ solution (10 mL) and the layers separated. The aqueous layer was washed with dichloromethane (3 x 5 mL) and the combined organic layers dried with $MgSO_4$ and the solvent removed *in vacuo*. The resulting crude mixture was purified by column chromatography eluting with chloroform: ethyl acetate (95:5) to give **1ab** as a clear oil (1.17 g, 7.65 mmol 92 %); $\delta_H$ (300 MHz, $CDCl_3$) 7.74 (1 H, dd, *J* = 2.9, 1.2 Hz, ArC-*H*), 7.48 (1 H, dd, *J* = 5.1, 3.0 Hz, ArC-*H*), 7.25 (1 H, dd, *J* = 5.3, 1.3 Hz, ArC-*H*), 2.78 (3 H, s, S-C$H_3$); $\delta_C$ (75 MHz, $CDCl_3$) 144.6 (Ar*C*), 128.5 (Ar*C*-H), 125.4 (Ar*C*-H), 122.6 (Ar*C*-H), 42.8 (S-*C*H$_3$); *m/z* (GCMS) 146.0; (measured 145.9856, $C_5H_6O_1S_2$ requires 145.9855).

**2-(Methylsulfinyl)thiophene 1ac**[1]

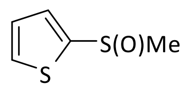


As described in General procedure **A**, to a solution of 2-(methylthio)thiophene (0.60 g, 4.60 mmol) in dichloromethane (5 mL, 1 M) at 0 $^{o}$C, was added dropwise a solution of *m*-CPBA (0.79 g, 4.60 mmol) in dichloromethane. The resulting crude mixture was purified by column chromatography eluting with chloroform: ethyl acetate (95:5) to give **1ac** as a clear oil (0.37 g, 2.46 mmol 58 %); $\delta_H$ (300 MHz, $CDCl_3$) 7.62 (1 H, dd, *J* = 5.0, 1.2 Hz, ArC-*H*), 7.45 (1 H, dd, *J* = 3.7, 1.2 Hz, ArC-*H*), 7.09 (1 H, dd, *J* = 5.0, 3.7 Hz, ArC-*H*), 2.89 (3 H, s, S-C$H_3$); $\delta_C$ (75 MHz,

[1] H. L. Hollan; C. D. Turner; P. R. Andreana; D. Nguyen *Can. J. Chem.*, **1999**, 77, 463

$CDCl_3$) 147.55 (ArC), 131.15 (ArC-H), 129.62 (ArC-H), 127.72 (ArC-H), 44.73 (S-$CH_3$); *m/z* (GCMS) 146.0; (measured 145.9855, $C_5H_6O_1S_2$ requires 145.9855)

**3-(Methylsulfinyl)furan 1ad**[1]

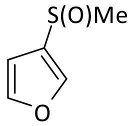

As described in General procedure **A**, to a solution of 3-(methylthio)thiophene (0.09 g, 0.78 mmol) in dichloromethane (1 mL, 1 M) at 0 °C, was added dropwise a solution of *m*-CPBA (0.13 g, 0.78 mmol) in dichloromethane. The resulting crude mixture was purified by column chromatography eluting with chloroform: ethyl acetate (95:5) to give **1ad** as a clear oil (0.06 g, 0.53 mmol 67 %); $\delta_H$ (300 MHz, $CDCl_3$) 7.75 (1 H, dd, *J* = 1.4, 0.9 Hz, ArC-*H*), 7.50 (1 H, t, *J* = 1.8 Hz, ArC-*H*), 6.67 (1 H, dd, *J* = 2.0, 0.8 Hz, ArC-*H*), 2.76 (3 H, s, S-$CH_3$); *m/z* (GCMS) 130.0; (measured 130.0078, $C_5H_6O_1S_2$ requires 130.0083).

**2-(methylsulfinyl)furan 1ae**[1]

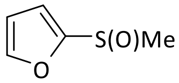

As described in General procedure **A**, to a solution of 2-(methylthio)thiophene (0.30 g, 2.63 mmol) in dichloromethane (2.6 mL, 1 M) at 0 °C, was added dropwise a solution of *m*-CPBA (0.43 g, 2.63 mmol) in dichloromethane. The resulting crude mixture was purified by column chromatography eluting with chloroform: ethyl acetate (95:5) to give **3ae** as a clear oil (0.26 g, 2.02 mmol 77 %); $\delta_H$ (300 MHz, $CDCl_3$) 7.59 - 7.67 (1 H, m, ArC-*H*), 6.92 (1 H, d, *J* = 3.4 Hz, ArC-*H*), 6.47 - 6.54 (1 H, m, ArC-*H*), 2.94 (3 H, s, S-$CH_3$); $\delta_C$ (75 MHz, $CDCl_3$) 152.8 (ArC), 146.7 (ArC-H), 115.2 (ArC-H), 111.4 (ArC-H), 38.6 (S-$CH_3$); *m/z* (GCMS) 130.0; (measured 130.0079, $C_5H_6O_1S_2$ requires 130.0083).

**3-(Phenylsulfinyl)thiophene 1af**

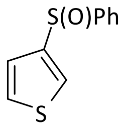

As described in General procedure **A**, to a solution of 3-(phenylthio)thiophene (0.60 g, 3.12 mmol) in dichloromethane (3 mL, 1 M) at 0 $^{o}$C, was added dropwise a solution of *m*-CPBA (0.53 g, 3.12 mmol) in dichloromethane. The resulting crude mixture was purified by flash column chromatography eluting with chloroform: ethyl acetate (95:5) to give **1af** as a clear oil (0.37 g, 1.87 mmol 60 %); $\nu_{max}$ (neat)/cm$^{-1}$ 3110, 3078, 1474, 1444, 1412, 1304, 1196, 1095, 1080, 1036, 999, 988, 971, 894, 803, 745, 684, 625; $\delta_H$ (300 MHz, $CDCl_3$) 7.07 (1H, dd, *J* 5.1, 1.2 Hz, HetAr-*H*), 7.39 (1H, dd, *J* 5.1, 3.0 Hz, HetAr-*H*), 7.45 – 7.55 (3H, m, Ar-*H*), 7.61 – 7.69 (2H, m, Ar-*H*), 7.79 (1H, dd, *J* 3.0, 1.2 Hz, HetAr-*H*); $\delta_C$ (75 MHz, $CDCl_3$) 124.1 (Ar*C*H), 124.6 (2 × Ar*C*H), 127.2 (Ar*C*H), 128.3 (Ar*C*H), 129.2 (2 × Ar*C*H), 131.0 (Ar*C*H), 144.6 (Ar*C*), 144.7 (Ar*C*); *m/z* (GCMS) 208.0; (measured 208.0006, $C_{10}H_8O_1S_2$ requires 208.0011).

**3-Bromo-4-(phenylsulfinyl)thiophene 1ag**

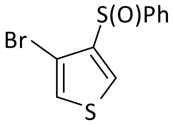


As described in General procedure **A**, to a solution of 4-bromo-3-(phenylthio)thiophene (0.27 g, 1.02 mmol) in dichloromethane (1 mL, 1 M) at 0 $^{o}$C, was added dropwise a solution of *m*-CPBA (0.17 g, 1.02 mmol) in dichloromethane. The resulting crude mixture was purified by flash column chromatography eluting with chloroform: ethyl acetate (95:5) to give **1ag** as a clear oil (0.20 g, 0.75 mmol 74 %); $\nu_{max}$ (neat)/cm$^{-1}$ 3064, 1580, 1474, 1442, 1399, 1330, 1157, 1099, 1079, 1038, 997, 924, 844, 800, 747, 684, 626, 547; $\delta_H$ (500 MHz, $CDCl_3$) 7.36 (1H, d, *J* 3.5 Hz, HetAr-*H*), 7.47 – 7.53 (3H, m, Ar-*H*), 7.74 – 7.79 (2H, m, Ar-*H*), 7.86 (1H, d, *J* 3.5 Hz, HetAr-*H*); $\delta_C$ (100 MHz, $CDCl_3$) 107.2 (Ar*C*), 125.7 (Ar*C*H), 125.9 (Ar*C*H), 127.9 (2 x Ar*C*H), 129.2 (2 × Ar*C*H), 131.6 (Ar*C*H), 143.3 (Ar*C*), 143.5 (Ar*C*); *m/z* (GCMS) 287.9; (measured 285.9919, $C_{10}H_7O_1Br_1S_2$ requires 285.9116).

**3-(Phenylsulfinyl)-4-(phenylthio)thiophene 1ah**

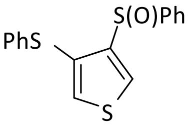

As described in General procedure **A**, to a solution of 3,4-*bis*(phenylthio)thiophene (0.40 g, 1.36 mmol) in dichloromethane (1.3 mL, 1 M) at 0 $^{o}$C, was added dropwise a solution of *m*-CPBA (0.23 g, 1.36 mmol) in dichloromethane. The resulting crude mixture was purified by flash column chromatography eluting with chloroform: ethyl acetate (95:5) to give **1ah** as a clear oil (0.10 g, 0.34 mmol 25 %); $\nu_{max}$ (neat)/cm$^{-1}$ 3064, 2033, 1477, 1438, 1110, 1078, 1037, 857, 837, 749, 735; $\delta_H$ (500 MHz, $CDCl_3$) 7.00 – 7.05 (2H, m, Ar-*H*), 7.13 – 7.22 (3H, m, Ar-*H*), 7.34 – 7.43 (3H, m, Ar-*H*), 7.47 (1H, d, *J* 3.4 Hz, HetAr-*H*), 7.65 – 7.70 (2H, m, Ar-*H*), 7.99 (1H, d, *J* 3.4 Hz, HetAr-*H*); $\delta_C$ (100 MHz, $CDCl_3$) 125.6 (2 × ArCH), 126.3 (ArC), 126.4 (ArCH), 127.9 (ArCH), 127.9 (2 × ArCH), 129.0 (2 × ArCH), 129.0 (2 × ArCH), 131.2 (ArCH), 132.9 (CH), 135.5 (C), 143.9 (C), 146.4 (C); *m/z* (GCMS) 316.0; (measured 316.0039, $C_{16}H_{12}O_1S_3$ requires 316.0045).

**3-(Phenylsulfinyl)furan 1aj**

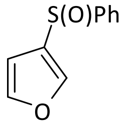


As described in General procedure **A**, to a solution of 3-(phenylthio)furan (0.41 g, 2.36 mmol) in dichloromethane (2.36 mL, 1 M) at 0 $^{o}$C, was added dropwise a solution of *m*-CPBA (0.57 g, 2.36 mmol) in dichloromethane. The resulting crude mixture was purified by flash column chromatography eluting with chloroform: ethyl acetate (95:5) to give **1aj** as a clear oil (0.34 g, 1.77 mmol 75 %); $\nu_{max}$ (neat)/cm$^{-1}$ 3180, 1580, 1498, 1443, 1201, 1133, 1084, 1021, 934, 852, 746, 687, 597; $\delta_H$ (300 MHz, $CDCl_3$) 6.40 (1H, dd, *J* 2.0, 0.8 Hz, HetAr-*H*), 7.42 – 7.47 (1H, m, HetAr-*H*), 7.48 – 7.56 (3H, m, Ar-*H*), 7.63 – 7.71 (2H, m, Ar-*H*), 7.81 (1H, dd, *J* 1.5, 0.8 Hz, HetAr-*H*); $\delta_C$ (75 MHz, $CDCl_3$) 107.3 (ArCH), 124.4 (2 × ArCH), 124.4 (ArC), 129.1 (2 × ArCH), 130.9 (ArCH), 143.7 (ArC), 144.1 (ArCH), 144.9 (ArCH); *m/z* (GCMS) 192.0; (measured 192.0233, $C_{10}H_8O_2S_1$ requires 192.0240).

**3,4-*bis*(Phenylsulfinyl)thiophene 1ak**

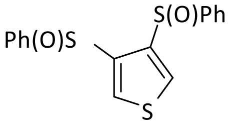

As described in General procedure **A**, to a solution of 3,4-*bis*(phenylthio)thiophene (0.40 g, 1.36 mmol) in dichloromethane (1.3 mL, 1 M) at 0 $^{o}$C, was added dropwise a solution of *m*-CPBA (0.23 g, 1.36 mmol) in dichloromethane. The resulting crude mixture was purified by flash column chromatography eluting with hexane: ethyl acetate (3:1) to give **1ak** as a mixture of diastereo-isomers and a clear oil (0.15 g, 0.45 mmol 33 %); $\nu_{max}$ (neat)/cm$^{-1}$ 3054, 2133, 1428, 1438, 1210, 1028, 1012, 849, 837, 779, 730; $\delta_H$ (400 MHz, $CDCl_3$) 7.36 − 7.52 (6H, m, Ar-*H*), 7.57 − 7.70 (4H, m, Ar-*H*), 7.81 (1H, s, Ar-*H* ), 7.85 (1H, s, Ar-*H*); $\delta_C$ (100 MHz, $CDCl_3$) 125.2 (2 × ArCH), 125.4 (2 × ArCH), 129.1 (2 × ArCH), 129.3 (2 × ArCH), 129.3 (ArCH), 130.0 (ArCH), 131.3 (ArCH), 131.6 (ArCH), 142.6 (ArC), 143.2 (ArC), 143.3 (ArC), 143.7 (ArC); *m/z* (GCMS) 332.0; (measured 331.9981, $C_{16}H_{12}O_2S_3$ requires 331.9994).

**General Procedure B Preparation of 1,3-*bis* propargyl silanes**

**(1,3-*bis*(Trimethylsilyl))-3-cyclohexyl-prop-1-yne 2l**[2]

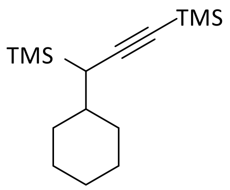


To a solution of 3-cyclohexyl-1-propyne (5 mmol, 0.89 ml) in THF (1 M, 5 ml) under nitrogen at -78 $^{o}$C was added *n*-butyllithium (1.6 M in hexanes, 5.25 mmol, 3.28 ml). The mixture was stirred at -78 $^{o}$C for 30 min before chlorotrimethylsilane (5.5 mmol, 0.69 ml) was added. The resultant white slurry was warmed to room temperature where it was maintained for 1 hour, after which it was cooled to -40 $^{o}$C and treated with *t*-butyllithium (1.7 M in pentane, 5.5 mmol, 3.23 ml). The yellow solution formed was then stirred at -40 $^{o}$C for 2 hours before addition of chlorotrimethylsilane (6 mmol, 0.76 ml). The reaction mixture was then allowed to warm to room temperature and was stirred for a further 1 hour before quenching the reaction with water (15 ml). The layers where separated and the organic layer extracted with diethyl ether (3 x 10 ml). The solution was then dried with $MgSO_4$ and the solvent

[2] S. Rajagopalan; G. Zweifel, *Synthesis,* **1984**, 2, 111-112

removed *in vacuo.* The crude product was purified using column chromatography on silica gel using 100% hexane as the eluent to give **2k** as a clear oil (0.153 g, 3.2 mmol, 64%); $\nu_{max}$ (neat)/$cm^{-1}$ 2926, 2852, 2147, 1889, 1656, 1449, 1247, 1053, 834, 757, 731, 693, 643; $\delta_H$ (400 MHz, $CDCl_3$) 0.11 (9H, s, Si(C$H_3$)$_3$), 0.14 (9H, s, Si(C$H_3$)$_3$), 1.09-1.49 (7H, m, cyclohexyl 7 x C*H*), 1.55-1.64 (2H, m, cyclohexyl 2 x C*H*), 1.67 (1H, d, J = 4.5, Si-C*H*), 1.71-1.74 (2H, m, cyclohexyl 2 x C*H*); $\delta_C$ (125 MHz) -1.8 (Si(*C*$H_3$)$_3$), 0.4 (Si(*C*$H_3$)$_3$), 26.0 (cyclohexyl-*C*$H_2$), 26.5 (cyclohexyl-*C*$H_2$), 26.6 (cyclohexyl-*C*$H_2$),29.2 (cyclohexyl-*C*$H_2$), 31.0 (cyclohexyl-*C*$H_2$), 34.1 (cyclohexyl-*C*H), 37.7 (Si-*C*H), 86.2 (C*C*Si), 108.5 (*C*CSi); *m*/*z* (GCMS) 266.1.

**(1,3-*bis*(Trimethylsilyl))-pent-1-yne 2h**[2]

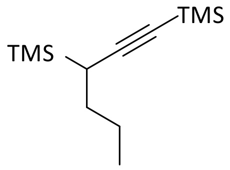


As described in general procedure **B**, to 1-hexyne (0.814 g, 10 mmol) ) was added *n*-BuLi (3.28 ml**,** 5.25 mmol), chlorotrimethylsilane (0.69 ml, 5.5 mmol), *t*-BuLi (3.23 ml, 5.5 mmol) and chlorotrimethylsilane (0.76 ml, 6.0 mmol). Purification by flash column chromatography using *n*-hexane as eluent gave **2h** (2.10 g, 9.29 mmol, 93 %) as a clear oil. $\nu_{max}$ (neat)/$cm^{-1}$ 2957, 2155, 1465, 1247, 1138, 1053, 962, 832, 757, 695, 636, 617; $\delta_H$ (500 MHz, $CDCl_3$) 0.08 (9H, s, Si(C$H_3$)$_3$), 0.13 (9H, s, Si(C$H_3$)$_3$), 0.92 (3H, t, *J* = 7.0 Hz, C$H_3$), 1.32 – 1.45 (3H, m), 1.62 – 1.71 (2H, m); $\delta_C$ (125 MHz) -3.6 (Si(*C*$H_3$)$_3$), 0.0 (Si(*C*$H_3$)$_3$), 13.4 (*C*$H_3$), 20.8 (*C*$H_2$), 22.3 (*C*$H_2$), 30.8 (*C*H), 84.0 (C*C*-Si), 109.7 (*C*C-Si); *m/z* (GCMS), 226.2.

**(1,3-*bis*(Trimethylsilyl))-pent-1-yne 2i**[3]

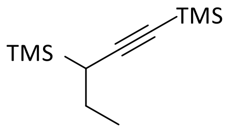


As described in general procedure **B**, to pent-1-yne (0.98 ml, 10 mmol) was added *n*-BuLi (6.56 ml, 10.5 mmol), chlorotrimethylsilane (1.38 ml, 11 mmol), *t*-BuLi (6.46 ml, 11 mmol) and chlorotrimethylsilane (1.52 ml, 12 mmol). Purification by flash column chromatography

[3] T. P. Lockhart; P. B. Comita; R. G. Bergman, *J. Am. Chem. Soc*, **1981**, 103, 14, 4082-4090

using *n*-hexane as eluent gave **2i** (0.53 g, 35 %) as a clear oil that gradually turned to clear pink on exposure to air. $\nu_{max}$ (neat)/cm$^{-1}$ 2958, 2151, 1406, 1248, 1146, 1067, 1027, 984, 896, 833, 757, 696, 658, 636; $\delta_H$ (400 MHz, CDCl$_3$) 0.08 (9H, s, Si(C*H*$_3$)$_3$), 0.14 (9H, s, Si(C*H*$_3$)$_3$), 1.07 (3H, t, J = 7.2, C*H*$_3$), 1.35-1.55 (2H, m, C*H*$_2$), 1.58-1.61 (1H, m, C*H*); $\delta_C$ (125 MHz) -3.5 (Si(*C*H$_3$)$_3$), 0.0 (Si(*C*H$_3$)$_3$), 13.9 (*C*H$_3$), 22.0 (*C*H), 23.1 (*C*H$_2$), 84.3 (C*C*-Si), 109.6 (*C*C-Si); *m/z* (GCMS), 212.2.

**(1,3-*bis*(Trimethylsilyl))-4-methyl-pent-1-yne 2j**

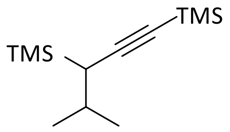


As described in general procedure **B**, to 4-methyl-1-pentyne (0.59 ml, 5 mmol) was added *n*-BuLi (3.28 ml**,** 5.25 mmol), chlorotrimethylsilane (0.69 ml, 5.5 mmol), *t*-BuLi (3.23 ml, 5.5 mmol) and chlorotrimethylsilane (0.76 ml, 6.0 mmol). Purification by flash column chromatography using *n*-hexane as eluent gave **2j** (0.63 g, 2.84 mmol, 57 %) as a clear oil. $\nu_{max}$ (neat)/cm$^{-1}$ 2972, 2359, 2342, 1159, 1104, 950, 627, 595, 555, 538; $\delta_H$ (400 MHz, CDCl$_3$) 0.11 (9H, s, Si(C*H*$_3$)$_3$), 0.14 (9H, s, Si(C*H*$_3$)$_3$), 0.98 (3H, d, J = 6.7, C*H*$_3$), 1.04 (3H, d, J = 6.6, C*H*$_3$), 1.69 (1H, d, J = 4.3, C*H*-Si(C*H*$_3$)$_3$), 1.86 (1H, m, C*H*(CH$_3$)$_2$); $\delta_C$ (125 MHz) 0.0 (Si(*C*H$_3$)$_3$), 2.4 (Si(*C*H$_3$)$_3$), 22.4 (*C*H-(Si(CH$_3$)$_3$), 26.0 (*C*H$_3$), 29.9 (*C*H$_3$), 31.9 (*C*H(CH$_3$)$_2$), 88.5 (C*C*-Si), 110.0 (*C*C-Si); *m/z* (GMCS), 226.1; (measured 226.1569 $C_{12}H_{26}Si_2$ requires 226.1573).

**(1,3-*bis*(Trimethylsilyl))-3-cyclopropyl-prop-1-yne 2k**

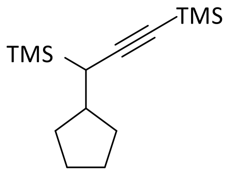


As described in general procedure **B**, to 3-cyclopentyl-1-propyne (0.65 ml, 5.0 mmol) was added *n*-BuLi (3.28 ml**,** 5.25 mmol), chlorotrimethylsilane (0.69 ml, 5.5 mmol), *t*-BuLi (3.23 ml, 5.5 mmol) and chlorotrimethylsilane (0.76 ml, 6.0 mmol). Purification by flash column chromatography using *n*-hexane as eluent gave **2k** (0.88 g, 4.92 mmol, 98 %) as a clear oil that gradually turned to clear pink on exposure to air. $\nu_{max}$ (neat)/cm$^{-1}$ 2954, 2151, 1247, 833, 757, 694, 644, 627; $\delta_H$ (400 MHz, CDCl$_3$) 0.10 (9H, s, Si(C*H*$_3$)$_3$), 0.14 (9H, s, Si(C*H*$_3$)$_3$),

1.38-1.54 (4H, m, cyclopentyl 2 x C$H_2$), 1.61-1.75 (4H, m, cyclopentyl 2 x C$H_2$), 1.82 (1H, d, J = 5.5, C*H*-Si), 1.89-1.97 (1H, m, cyclopentyl C*H*); $\delta_C$ (125 MHz) -1.9 (Si(C$H_3$)$_3$), 0.7 (Si(C$H_3$)$_3$), 25.7 (cyclopentyl C$H_2$), 25.8 (cyclopentyl C$H_2$), 30.9 (cyclopentyl C$H_2$), 33.7 (cyclopentyl C$H_2$), 39.6 (cyclopentyl *C*H), 85.5 (*C*CSi), 109.1 (C*C*Si); *m/z* (GCMS) 252.3; 25; (measured 252.1725 $C_{14}H_{28}Si_2$ requires 252.1729).

**General Procedure C Preparation of Propargyl Silanes**

**2-Trimethylsilyl-hex-3-yne 2m**

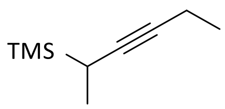


To a solution of 4-octyne (0.56 ml, 5 mmol) in THF (1 M) under nitrogen at -40 $^oC$ was added *t*-BuLi (1.7 M in pentane, 3.23 ml, 5 mmol). The mixture was stirred at -40 $^oC$ for 2 hours before the addition of chlorotrimethylsilane (0.75 ml, 10 mmol). The reaction was then allowed to warm to room temperature and stirred for an additional hour before the addition of water 15 ml. The resultant layers were separated and the aqueous phase extracted with diethyl ether (3 x 15 ml) and the organic phases combined, dried with $MgSO_4$, and the solvent removed *in vacuo*. The crude mixture was purified by flash column chromatography on silica gel using 100% hexane as the eluent to give **2m** as a clear oil (0.18 g, 1.2 mmol, 24 %); $\nu_{max}$ (neat)/cm$^{-1}$ 2958, 1715, 1680, 1461, 1319, 1247, 1057, 1000, 970, 835, 753, 692, 642, 582; $\delta_H$ (400 MHz, $CDCl_3$) 0.06 (9H, s, Si(C$H_3$)$_3$), 1.10-1.14 (6H, m, 2 x C$H_3$), 1.58-1.64 (1H, m, C*H*), 2.17 (2H, qd, J = 7.5, 2.6, C$H_2$); $\delta_C$ (125 MHz) -3.9 (Si(C$H_3$)$_3$), 12.3 (C$H_3$), 12.5 (C$H_3$), 14.4 (C$H_2$), 14.7 (*C*H-Si), 80.7 (*C*C), 82.4 (C*C*); *m/z* (GCMS) 154.1; (measured 154.1172 $C_9H_{18}Si_1$ requires 154.1177).

**3-Trimethylsilyl-oct-4-yne 2n**

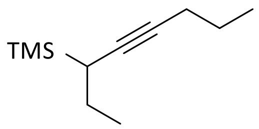

As described in general procedure **C**, to 4-octyne (1.46 ml, 10 mmol) was added *t*-BuLi (6.46 ml, 5 mmol) and chlorotrimethylsilane (1.5 mL, 10 mmol). Purification by flash column chromatography using *n*-hexane as eluent gave **2n** (1.16 g, 6.4 mmol, 64 % yield) as a clear oil; $\nu_{max}$ (neat)/cm$^{-1}$ 2959, 2932, 2871, 1714, 1461, 1377, 1338, 1247, 1159, 1046, 836, 757, 692, 613, 572; $\delta_H$ (400 MHz, CDCl$_3$) 0.07 (9H, s, Si(C*H*$_3$)$_3$), 0.98 (3H, t, J = 7.4, C*H*$_3$), 1.07 (3H, t, J = 7.2, C*H*$_3$), 1.29-1.40 (1H, m, C*H*), 1.47-1.54 (4H, m, 2 x C*H*$_2$), 2.16 (2H, td, J = 6.9, 2.5, C*H*$_2$); $\delta_C$ (100 MHz) -3.1 (Si(*C*H$_3$)$_3$), 13.5 (*C*H$_3$), 14.4 (*C*H$_3$), 21.0 (*C*H$_2$), 21.9 (*C*H$_2$), 22.8 (*C*H$_2$), 22.9 (*C*H-Si), 80.7 (*C*C), 81.7 (C*C*); *m/z* (GCMS) 182.2; (measured 182.1487 $C_{11}H_{22}Si_1$ requires 182.1490).

**Propargylation of Heterocycles**

**2-(Hept-2-ynyl)-3-(phenylthio)thiophene 3ab**

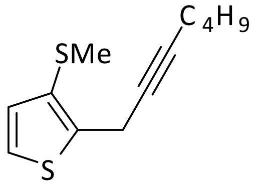


To an oven dried tube flushed with $N_2$, was added 3-(methylsulfinyl)thiophene (0.080 g, 0.54 mmol) and hept-2-yn-1-yltrimethylsilane (0.13 g, 0.81 mmol) in MeCN (3.3 mL). Trifluoroacetic anhydride was then added (0.19 mL, 1.35 mmol) at -40 $^{o}$C and the reaction was allowed to warm to room temperature over 18 hours. The solution was quenched with aqueous saturated $NaHCO_3$ (6 mL) and the aqueous layer was extracted with EtOAc (3 x 5 mL). The combined organic layer was dried ($MgSO_4$) and concentrated *in vacuo*. The crude product was purified by column chromatography on silica gel eluting with 100% *n*-hexane to yield **3ab** as a colourless oil (0.10 g, 0.45 mmol, 84 %). $\nu_{max}$ (neat)/cm$^{-1}$ 2955, 2927, 2870, 1508, 1464, 1431,1377, 1346, 1315, 1294, 1198, 1153, 1077, 969, 875, 853, 778, 704, 636,572; $\delta_H$ (400 MHz, $CDCl_3$) 0.92 (3H, t, *J* 7.2 Hz, C*H*$_3$), 1.38 – 1.55 (4H, m, C*H*$_2$C*H*$_2$), 2.21 (2H, tt, *J* 7.0, 2.4 Hz, CC*H*$_2$), 2.38 (3H, s, S-C*H*$_3$), 3.77 (2H, t, *J* 2.4 Hz, HetAr-C*H*$_2$C), 6.99 (1H, d, *J* 5.3 Hz, HetAr-*H*), 7.17 (1H, d, *J* 5.3 Hz, HetAr-*H*); $\delta_C$ (100 MHz, $CDCl_3$) 13.6 ($CH_3$), 18.4 ($CH_2$), 18.9 ($CH_3$), 18.9 ($CH_2$), 21.9 ($CH_2$), 30.8 ($CH_2$), 76.9 (C), 82.4 (C), 122.9 (ArCH), 128.7 (ArC), 129.9 (ArCH), 139.7 (ArC); *m/z* (GCMS) 224.0; (measured 224.0689, $C_{12}H_{16}S_2$ requires 224.0693).

**3-(Hept-2-yn-1-yl)-2-(methylthio)thiophene 3ac**

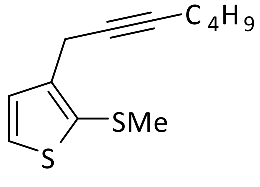


To an oven dried tube flushed with $N_2$, was added 2-(methylsulfinyl)thiophene (14.5 mg, 0.09 mmol) and hept-2-yn-1-yltrimethylsilane (25 mg, 0.13 mmol) in MeCN (3 mL). Trifluoroacetic anhydride was then added (0.03 mL, 0.25 mmol) at -40 $^{o}$C and the reaction

was allowed to warm to room temperature over 18 hours. The solution was quenched with aqueous saturated $NaHCO_3$ (6 mL) and the aqueous layer was extracted with EtOAc (3 x 5 mL). The combined organic layer was dried ($MgSO_4$) and concentrated *in vacuo*. The crude product was purified by column chromatography on silica gel eluting with 100% *n*-hexane to yield **3ac** as a colourless oil (0.06 g, 0.07 mmol, 74 %). $\nu_{max}$ (neat)/cm$^{-1}$ 2955, 2922, 2858, 1728, 1523, 1464, 1420, 1377, 1312, 1216, 1093, 1020, 969, 877, 848, 831, 689, 651, 579; $\delta_H$ (400 MHz, $CDCl_3$) 0.91 (3H, t, *J* 7.2 Hz, C*H*$_3$), 1.33 – 1.55 (4H, m, C*H*$_2$C*H*$_2$), 2.19 (2H, tt, *J* 7.0, 2.4 Hz, CC*H*$_2$), 2.39 (3H, s, S-C*H*$_3$), 3.60 (2H, t, *J* 2.4 Hz, HetAr-C*H*$_2$C), 7.12 (1H, d, *J* 5.4 Hz, HetAr-*H*), 7.28 (1H, d, *J* 5.4 Hz, HetAr-*H*); $\delta_C$ (100 MHz, $CDCl_3$) 13.6 ($CH_3$), 18.5 ($CH_2$), 19.0 ($CH_2$), 21.9 ($CH_3$), 21.9 ($CH_2$), 31.0 ($CH_2$), 77.4 (C), 81.5 (C), 127.1 (ArCH), 129.0 (ArCH), 130.6 (ArC), 141.4 (ArC); *m/z* (GCMS) 209.0 (M-$CH_3$); (measured 225.0780, $C_{12}H_{17}S_2$ requires 225.0772).

**2-(Hept-2-yn-1-yl)-3-(methylthio)furan 3ad**

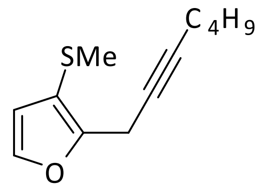


To an oven dried tube flushed with $N_2$, was added 3-(methylsulfinyl)furan (0.057 g, 0.43 mmol) and hept-2-yn-1-yltrimethylsilane (0.11 g, 0.64 mmol) in MeCN (3 mL). Trifluoroacetic anhydride was then added (0.15 mL, 1.0 mmol) at -40 $^oC$ and the reaction was allowed to warm to room temperature over 18 hours. The solution was quenched with aqueous saturated $NaHCO_3$ (6 mL) and the aqueous layer was extracted with EtOAc (3 x 5 mL). The combined organic layer was dried ($MgSO_4$) and concentrated *in vacuo*. The crude product was purified by column chromatography on silica gel eluting with 100% *n*-hexane to yield **3ad** as a Colourless oil (0.077 g, 0.35 mmol, 82 %). $\nu_{max}$ (neat)/cm$^{-1}$ 2956, 2928, 2871, 1771, 1582, 1465, 1378, 1248, 1193, 1054, 1006, 971, 988, 880, 728; $\delta_H$ (500 MHz, $CDCl_3$) 0.90 (3H, t, *J* 7.3 Hz, C*H*$_3$), 1.34 – 1.44 (2H, m, CH$_2$C*H*$_2$), 1.44 – 1.52 (2H, m, C*H*$_2$CH$_2$), 2.17 (2H, tt, *J* 7.1, 2.4 Hz, CC*H*$_2$), 2.30 (3H, s, S-C*H*$_3$), 3.65 (2H, t, *J* 2.4 Hz, HetAr-C*H*$_2$C), 6.39 (1H, d, *J* 1.9 Hz, HetAr-*H*), 7.34 (1H, d, *J* 1.9 Hz, HetAr-*H*); $\delta_C$ (125 MHz, $CDCl_3$) 13.6 ($CH_3$), 16.7 ($CH_2$), 18.4 ($CH_2$), 19.3 ($CH_3$), 21.9 ($CH_2$), 30.9 ($CH_2$), 74.4 (C), 81.5 (C), 113.0 (ArC), 114.0 (ArCH), 141.4 (ArCH), 151.4 (ArC): *m/z* (GCMS) 208.0; (measured 208.0906 $C_{12}H_{16}O_1S_1$ requires 208.0916).

**3-(Hept-2-yn-1-yl)-2-(methylthio)furan 3ae**

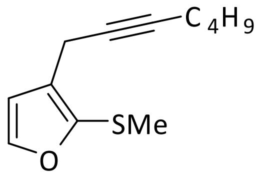

To an oven dried tube flushed with $N_2$, was added 2-(methylsulfinyl)furan (0.059 g, 0.45 mmol) and hept-2-yn-1-yltrimethylsilane (0.11 g, 0.67 mmol) in MeCN (3 mL). Trifluoroacetic anhydride was then added (0.16 mL, 1.12 mmol) at -40 $^{o}$C and the reaction was allowed to warm to room temperature over 18 hours. The solution was quenched with aqueous saturated $NaHCO_3$ (6 mL) and the aqueous layer was extracted with EtOAc (3 x 5 mL). The combined organic layer was dried ($MgSO_4$) and concentrated *in vacuo*. The crude product was purified by column chromatography on silica gel eluting with 100% *n*-hexane to yield **3ae** as a colourless oil (0.082 g, 0.41 mmol, 92 %). $\nu_{max}$ (neat)/cm$^{-1}$ 2933, 1685, 1574, 1467, 1425, 1390, 1321, 1250, 1196, 1144, 1064, 996, 900, 884, 859, 755, 649; $\delta_H$ (300 MHz, $CDCl_3$) 0.91 (3H, t, *J* 7.2 Hz, C*H*$_3$), 1.31 – 1.56 (4H, m C*H*$_2$C*H*$_2$), 2.18 (2H, tt, *J* 6.9, 2.4 Hz, CC*H*$_2$), 2.33 (3H, s, S-C*H*$_3$), 3.40 (2H, t, *J* 2.4 Hz, HetAr-C*H*$_2$C), 6.48 (1H, d, *J* 2.0 Hz, HetAr-*H*), 7.44 (1H, d, *J* 2.0 Hz, HetAr-*H*); $\delta_C$ (75 MHz, $CDCl_3$) 13.6 ($CH_3$), 16.0 ($CH_2$), 18.4 ($CH_2$), 18.9 ($CH_3$), 21.9 ($CH_2$), 31.0 ($CH_2$), 77.1 (C), 81.0 (C), 112.4 (ArCH), 126.4 (ArC), 142.9 (ArC), 144.4 (ArCH); *m/z* (GCMS) 208.0; (measured 208.0910 $C_{12}H_{16}O_1S_1$ requires 208.0916).

**2-(Hept-2-yn-1-yl)-3-(phenylthio)thiophene 3af**

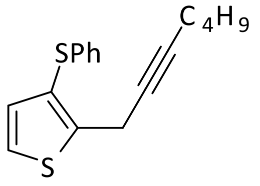

To an oven dried-tube flushed with $N_2$, was added 3-(phenylsulfinyl)thiophene (0.087 g, 0.41 mmol) and hept-2-yn-1-yltrimethylsilane (0.10 g, 0.62 mmol) in MeCN (3 mL). Trifluoroacetic anhydride was then added (0.14 mL, 1.0 mmol) at room temperature and the reaction was stirred for 2 hours. The solution was quenched with aqueous saturated $NaHCO_3$ (6 mL) and the aqueous layer was extracted with EtOAc (3 x 5 mL). The combined organic layer was

dried ($MgSO_4$) and concentrated *in vacuo*. The crude product was purified by column chromatography on silica gel eluting with 100% *n*-hexane to yield **3af** as a colourless oil (0.11 g, 0.38 mmol, 93 %). $\nu_{max}$ (neat)/cm$^{-1}$ 3072, 2955, 2929, 2870, 1661, 1581, 1521, 1439, 1398, 1297, 1218, 1135, 1080, 1023, 998, 832, 737, 688; $\delta_H$ (300 MHz, $CDCl_3$) 0.92 (3H, t, *J* 7.2 Hz, C*H*$_3$), 1.33 – 1.54 (4H, m, C*H*$_2$C*H*$_2$), 2.19 (2H, tt, *J* 6.8, 2.4 Hz, CC*H*$_2$), 3.77 (2H, t, *J* 2.4 Hz, HetAr-C*H*$_2$C), 6.99 (1H, d, *J* 5.2 Hz, HetAr-*H*), 7.03 – 7.16 (3H, m, Ar-*H*), 7.24 (1H, d, *J* 5.2 Hz, HetAr-*H*), 7.17 – 7.26 (2H, m, Ar-*H*); $\delta_C$ (75 MHz, $CDCl_3$) 13.6 ($CH_3$), 18.4 ($CH_2$), 19.2 ($CH_2$), 21.9 ($CH_2$), 30.8 ($CH_2$), 77.2 (C), 82.8 (C), 123.3 (ArCH), 123.9 (ArC), 125.5 (ArCH), 127.0 (2 × ArCH), 128.9 (2 × ArCH), 132.6 (ArCH), 137.5 (ArC), 145.3 (ArC); *m/z* (GCMS) 209.0 (M-$C_6H_5$); (measured 286.0833, $C_{17}H_{18}S_2$ requires 286.0844).

**4-Bromo-2-(hept-2-yn-1-yl)-3-(phenylthio)thiophene 3ag**

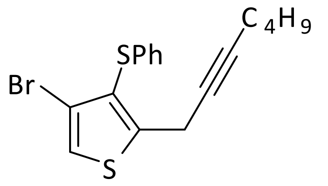


To an oven-dried tube flushed with $N_2$, was added 3-bromo-4-(phenylsulfinyl)thiophene (0.080 g, 0.27 mmol) and hept-2-yn-1-yltrimethylsilane (0.070 g, 0.41 mmol) in MeCN (3 mL). Trifluoroacetic anhydride (0.097 mL, 0.67 mmol) was then added at room temperature and the reaction was stirred for 2 hours. The solution was quenched with aqueous saturated $NaHCO_3$ (6 mL) and the aqueous layer was extracted with EtOAc (3 x 5 mL). The combined organic layer was dried ($MgSO_4$) and concentrated *in vacuo*. The crude product was purified by column chromatography on silica gel eluting with 100% *n*-hexane to yield **3ag** as a colourless oil (0.083 g, 0.23 mmol, 86 %). $\nu_{max}$ (neat)/cm$^{-1}$ 2955, 2928, 2869, 1724, 1581, 1477, 1439, 1377, 1317, 1081, 1024, 906, 734, 687, 630; $\delta_H$ (400 MHz, $CDCl_3$) 0.92 (3H, t, *J* 7.2 Hz, C*H*$_3$), 1.36 – 1.55 (4H, m, C*H*$_2$C*H*$_2$), 2.18 (2H, tt, *J* = 6.8, 2.4 Hz, CC*H*$_2$), 3.82 (2H, t, *J* = 2.4 Hz, HetAr-C*H*$_2$C), 7.00 – 7.08 (2H, m, Ar-*H*), 7.10 – 7.17 (1H, m, Ar-*H*), 7.18 – 7.25 (2H, m, Ar-*H*), 7.31 (1H, s, HetAr-*H*); $\delta_C$ (100 MHz, $CDCl_3$) 13.6 ($CH_3$), 18.3 ($CH_2$), 20.8 ($CH_2$), 21.9 ($CH_2$), 30.7 ($CH_2$), 75.6 (C), 83.8 (C), 117.4 (ArC), 121.0 (ArCH), 124.9 (ArC), 125.6 (ArCH), 126.5 (2 × ArCH), 128.9 (2 × ArCH), 136.2 (ArC), 148.1 (ArC); *m/z* (GCMS) 364.0; (measured 363.9943 $C_{17}H_{17}Br_1S_1$ requires 363.9950).

**2-(Hept-2-yn-1-yl)-3,4-bis(phenylthio)thiophene 3ah**

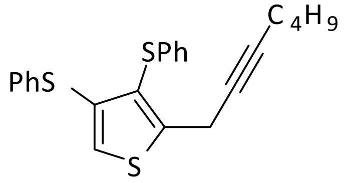


To an oven-dried tube flushed with $N_2$, was added 3-(phenylsulfinyl)-4-(phenylthio)thiophene (0.080 g, 0.25 mmol) and hept-2-yn-1-yltrimethylsilane (0.063 g, 0.37 mmol) in MeCN (3 mL). Trifluoroacetic anhydride (0.088 mL, 0.92 mmol) was then added at room temperature and the reaction was stirred for 2 hours. The solution was quenched with aqueous saturated $NaHCO_3$ (6 mL) and the aqueous layer was extracted with EtOAc (3 x 5 mL). The combined organic layer was dried ($MgSO_4$) and concentrated *in vacuo*. The crude product was purified by column chromatography on silica gel eluting with 100% *n*-hexane to yield **3ah** as a colourless oil (0.071 g, 0.27 mmol, 75 %). $\nu_{max}$ (neat)/cm$^{-1}$ 3057, 2955, 2928, 2869, 1666, 1581, 1476, 1377, 1308, 1081, 1023, 998, 948, 907, 853, 734, 687; $\delta_H$ (500 MHz, $CDCl_3$) 0.89 (3H, t, *J* 7.2 Hz, C*H*$_3$), 1.35 – 1.43 (2H, m, $CH_2$C*H*$_2$), 1.43 – 1.50 (2H, m, C*H*$_2$$CH_2$), 2.16 (2H, tt, *J* 7.0, 2.4 Hz, CC*H*$_2$), 3.75 (2H, t, *J* 2.4 Hz, HetAr-C*H*$_2$C), 6.94 – 7.00 (2H, m, Ar-*H*), 7.04 – 7.08 (1H, m, Ar-*H*), 7.09 (1H, s, HetAr-*H*), 7.12 – 7.18 (3H, m, Ar-*H*), 7.18 – 7.26 (4H, m, Ar-*H*); $\delta_C$ (125 MHz, $CDCl_3$) 13.6 ($CH_3$), 18.4 ($CH_2$), 20.4 ($CH_2$), 21.9 ($CH_2$), 30.8 ($CH_2$), 75.9 (C), 83.5 (C), 124.0 (ArCH), 125.4 (ArCH), 125.6 (ArC), 126.7 (2 × ArCH), 126.8 (ArCH), 128.8 (2 × ArCH), 129.0 (2 × ArCH), 130.4 (2 × ArCH), 135.4 (ArC), 135.9 (ArC), 136.6 (ArC), 148.8 (ArC); *m/z* (GCMS) 394.1; (measured 394.0875 $C_{23}H_{22}S_3$ requires 394.0878).

**3-(Hept-2-yn-1-yl)-2-(phenylthio)thiophene 3ai**

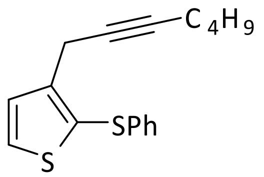


To an oven dried tube flushed with $N_2$, was added 2-(phenylsulfinyl)thiophene (0.029 g, 0.14 mmol) and hept-2-yn-1-yltrimethylsilane (0.058 g, 0.35 mmol) in MeCN (3 mL). Trifluoroacetic anhydride (0.058 mL, 0.35 mmol) was then added at -78 °C and the reaction

was stirred for 2 hours. The solution was quenched with aqueous saturated $NaHCO_3$ (6 mL) and the aqueous layer was extracted with EtOAc (3 x 5 mL). The combined organic layer was dried ($MgSO_4$) and concentrated *in vacuo*. The crude product was purified by column chromatography on silica gel eluting with 100% *n*-hexane to yield **3ai** as a colourless oil (0.031 g, 0.11 mmol, 80 %). $\nu_{max}$ (neat)/cm$^{-1}$ 2955, 2928, 2869, 1724, 1581, 1477, 1439, 1377, 1317, 1081, 1024, 906, 734, 687, 630; $\delta_H$ (300 MHz, $CDCl_3$) 0.91 (3H, t, *J* 7.2 Hz, $CH_3$), 1.29 – 1.53 (4H, m, $CH_2CH_2$), 2.16 (2H, tt, *J* 6.9, 2.4 Hz, $CCH_2$), 3.56 (2H, t, *J* 2.4 Hz, HetAr-$CH_2$C), 7.02 – 7.18 (3H, m, Ar-*H*), 7.18 – 7.32 (2H, m, Ar-*H*), 7.27 (1H, d, *J* 5.4 Hz, HetAr-*H*), 7.45 (1H, d, *J* 5.4 Hz, HetAr-*H*); $\delta_C$ (75 MHz, $CDCl_3$) 13.6 ($CH_3$), 18.4 ($CH_2$), 19.2 ($CH_2$), 21.9 ($CH_2$), 31.0 ($CH_2$), 77.2 (C), 81.9 (C), 124.7 (ArC), 125.7 (ArCH), 126.5 (2 ×ArCH), 129.0 (2 × ArCH), 129.4 (ArCH), 129.6 (ArCH), 138.0 (ArC), 144.8 (ArC); *m/z* (GCMS) 209.0 (M-$C_6H_5$); (measured 286.0834, $C_{17}H_{18}S_2$ requires 286.0844).

**2-(Hept-2-yn-1-yl)-3-(phenylthio)furan 3aj**

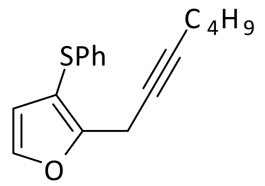


To an oven dried tube flushed with $N_2$, was added 3-(phenylsulfinyl)furan (0.030 g, 0.15 mmol) and hept-2-yn-1-yltrimethylsilane (0.039 g, 0.23 mmol) in MeCN (3 mL). Trifluoroacetic anhydride (0.054 mL, 0.39 mmol) was then added at -20 °C and the reaction allowed to warm to room temperature over 2 hours. The solution was quenched with aqueous saturated $NaHCO_3$ (6 mL) and the aqueous layer was extracted with EtOAc (3 x 5 mL). The combined organic layer was dried ($MgSO_4$) and concentrated *in vacuo*. The crude product was purified by column chromatography on silica gel eluting with 100% *n*-hexane to yield **3aj** as a colourless oil (0.081 g, 0.11 mmol, 74 %). $\nu_{max}$ (neat)/cm$^{-1}$ 2956, 2930, 2870, 1582, 1511, 1477, 1412, 1305, 1249, 1195, 1123, 1084, 1024, 1008, 885, 736, 689, 652, 558; $\delta_H$ (300 MHz, $CDCl_3$) 0.89 (3H, t, *J* 7.1 Hz, $CH_3$), 1.28 – 1.52 (4H, m, $CH_2CH_2$), 2.14 (2H, tt, *J* 6.9, 2.4 Hz, $CCH_2$), 3.68 (2H, t, *J* 2.4 Hz, HetAr-$CH_2$C), 6.40 (1H, d, *J* 1.9 Hz, HetAr-*H*), 7.08 – 7.18 (3H, m, Ar-*H*), 7.18 – 7.26 (2H, m, Ar-*H*), 7.45 (1H, d, *J* 1.9 Hz, HetAr-*H*); $\delta_C$ (75 MHz, $CDCl_3$) 13.6 ($CH_3$), 16.8 ($CH_2$), 18.4 ($CH_2$), 21.9 ($CH_2$), 30.8 ($CH_2$), 77.2 (C), 82.8 (C), 123.3

(ArCH), 123.9 (ArC), 125.5 (ArCH), 127.0 (2 × ArCH), 128.9 (2 × ArCH), 132.6 (ArCH), 137.5 (ArC), 145.3 (ArC); *m/z* (GCMS) 271.7; (measured 270.1080 $C_{17}H_{18}O_1S_3$ requires 270.1073).

**2,5-Di(hept-2-yn-1-yl)-3,4-*bis*(phenylthio)thiophene 3ak**

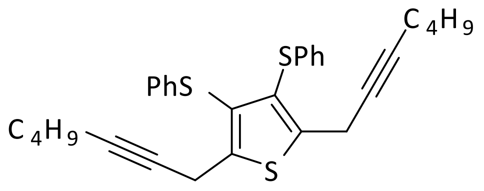


To an oven dried tube flushed with $N_2$, was added 3,4-*bis*(phenylsulfinyl)thiophene (0.058 g, 0.17 mmol) and hept-2-yn-1-yltrimethylsilane (0.088 g, 0.51 mmol) in MeCN (1.5 mL). Trifluoroacetic anhydride (0.098 mL, 0.85 mmol) was then added at room temperature and the reaction was stirred for 2 hours. The solution was quenched with aqueous saturated $NaHCO_3$ (6 mL) and the aqueous layer was extracted with EtOAc (3 x 5 mL). The combined organic layer was dried ($MgSO_4$) and concentrated *in vacuo*. The crude product was purified by column chromatography on silica gel eluting with 100% *n*-hexane to yield **3ak** as a colourless oil (0.045 g, 0.10 mmol, 62 %). $\nu_{max}$ (neat)/cm$^{-1}$ 2956, 2930, 2870, 1717, 1663, 1582, 1477, 1439, 1378, 1082, 1023, 737, 688, 603, 581; $\delta_H$ (500 MHz, $CD_3CN$) 0.90 (6H, t, *J* 7.2 Hz, 2 × C$H_3$), 1.36 – 1.49 (8H, m, 2 × C$H_2$C$H_2$), 2.18 (4H, tt, *J* 6.8, 2.4 Hz, 2 × CC$H_2$), 3.80 (4H, t, *J* 2.4 Hz, 2 × HetAr-C$H_2$C), 6.93 – 6.97 (4H, m, Ar-*H*), 7.07 – 7.11 (2H, m, Ar-*H*), 7.15 – 7.20 (4H, m, Ar-*H*); $\delta_C$ (125 MHz, $CD_3CN$) 13.9 (2 × $CH_3$), 18.9 (2 × $CH_2$), 20.7 (2 × $CH_2$), 22.7 (2 × $CH_2$), 31.6 (2 × $CH_2$), 77.1 (2 × C), 84.3 (2 × C), 126.6 (2 × ArCH), 127.5 (4 × ArCH), 128.8 (2 × ArC), 130.0 (4 × ArCH), 137.9 (2 × ArC), 146.9 (2 × ArC); *m/z* (GCMS) 488.1; (measured 488.1647 $C_{30}H_{32}S_3$ requires 488.1661).

**(3-(3-(Methylthio)thiophen-2-yl)prop-1-yn-1-yl)trimethylsilane 3aq**

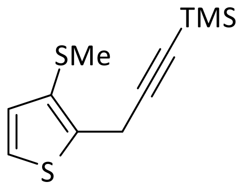

To an oven-dried tube flushed with $N_2$, was added 3-(methylsulfinyl)thiophene (0.032 g, 0.21 mmol) and 1,3-bis(trimethylsilyl)-prop-1-yne (0.060 g, 0.32 mmol) in MeCN (3 mL). Trifluoroacetic anhydride (0.077 mL, 0.52 mmol) was then added at -40 $^{o}$C and the reaction was allowed to warm to room temperature over 18 hours. The solution was quenched with aqueous saturated $NaHCO_3$ (6 mL) and the aqueous layer was extracted with EtOAc (3 x 5 mL). The combined organic layer was dried ($MgSO_4$) and concentrated *in vacuo*. The crude product was purified by column chromatography on silica gel eluting with 100% *n*-hexane to yield **3aq** as a colourless oil (0.031 g, 0.13 mmol, 64 %). $\nu_{max}$ (neat)/cm$^{-1}$ 2921, 1249, 1025, 842, 759; $\delta_H$ (500 MHz, $CDCl_3$) 0.19 (9H, s, Si(C*H*$_3$)$_3$), 2.38 (3H, s, S-C*H*$_3$), 3.84 (2H, s, HetAr-C*H*C), 7.00 (1H, d, *J* 5.3 Hz, HetAr-*H*), 7.19 (1H, d, *J* 5.3 Hz, HetAr-*H*); $\delta_C$ (100 MHz, $CDCl_3$) 0.1 (3 × $CH_3$), 19.0 ($CH_3$), 20.0 ($CH_2$), 86.7 (C), 103.3 (C), 123.2 (ArCH), 129.3 (ArC), 130.1 (ArCH), 138.3 (ArC); *m/z* (GCMS) 240.0; (measured 240.0447 $C_{11}H_{16}S_2Si_1$ requires 240.0457).

**2-(3-Cyclohexylprop-2-yn-1-yl)-3-(methylthio)thiophene 3ar**

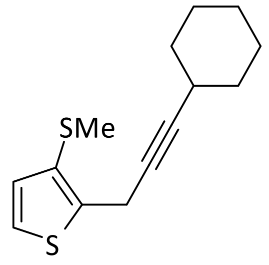


To an oven-dried tube flushed with $N_2$, was added 3-(methylsulfinyl)thiophene (0.060 g, 0.41 mmol) and (3-cyclohexylprop-2-yn-1-yl)trimethylsilane (0.11 g, 0.61 mmol) in MeCN (3 mL). Trifluoroacetic anhydride (0.14 mL, 1.02 mmol) was then added at -40 $^{o}$C and the reaction was allowed to warm to room temperature over 18 hours. The solution was quenched with aqueous saturated $NaHCO_3$ (6 mL) and the aqueous layer was extracted with EtOAc (3 x 5 mL). The combined organic layer was dried ($MgSO_4$) and concentrated *in vacuo*. The crude product was purified by column chromatography on silica gel eluting with 100% *n*-hexane to yield **3ar** as a colourless oil (0.071 g, 0.29 mmol, 71 %). $\nu_{max}$ (neat)/cm$^{-1}$ 2925, 2851, 1709, 1651, 1499, 1332, 1291, 1205, 1157, 1027, 969, 888, 859, 769, 709, 642, 609; $\delta_H$ (500 MHz, $CDCl_3$) 1.13 – 1.28 (3H, m), 1.31 – 1.45 (3H, m), 1.58 – 1.66 (2H, m, C*H*$_2$), 1.66 – 1.76 (2H, m, C*H*$_2$), 2.24 – 2.35 (1H, m, CC*H*), 2.27 (3H, s, S-C*H*$_3$), 3.68 (2H, d, *J* 2.2 Hz HetAr-C*H*C), 6.88 (1H, d, *J* 5.3 Hz, HetAr-*H*), 7.06 (1H, d, *J* 5.3 Hz, HetAr-*H*); $\delta_C$ (125 MHz, $CDCl_3$)

18.8 ($CH_3$), 18.9 ($CH_2$), 24.8 ($CH_2$), 25.9 (2 × $CH_2$), 29.0 (CH), 32.7 (2 × $CH_2$), 76.9 (C), 86.7(C), 122.8 (ArCH), 128.6 (ArC), 129.9 (ArCH), 139.9 (ArC); *m/z* (GCMS) 235.1 (-$CH_3$) ; (measured 235.0603 $C_{13}H_{15}S_2$ requires 235.0610).

**(3-(3-(Methylthio)thiophen-2-yl)hex-1-yn-1-yl)trimethylsilane 3at**

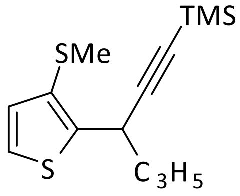


To an oven-dried tube flushed with $N_2$, was added 3-(methylsulfinyl)thiophene (0.06 g, 0.41 mmol) and 1,3-*bis*(trimethylsilane)hex-1-yne (0.13 g, 0.61 mmol) in MeCN (3 mL). Trifluoroacetic anhydride (0.14 mL, 0.61 mmol) was then added at -40 $^{o}C$ and the reaction was allowed to warm to room temperature over 18 hours. The solution was quenched with aqueous saturated $NaHCO_3$ (6 mL) and the aqueous layer was extracted with EtOAc (3 x 5 mL). The combined organic layer was dried ($MgSO_4$) and concentrated *in vacuo*. The crude product was purified by column chromatography on silica gel eluting with 100% *n*-hexane to yield **3at** as a colourless oil (0.070 g, 0.26 mmol, 64 %). $\nu_{max}$ (neat)/$cm^{-1}$ 2957, 2169, 1248,1029, 953, 838, 759, 700, 666, 637; $\delta_H$ (500 MHz, $CDCl_3$) 0.18 (9H, s, Si(C$H_3$)$_3$), 0.95 (3H, t, *J* 7.4 Hz, C$H_3$), 1.40 – 1.62 (2H, m, $CH_3$C$H_2$), 1.68 – 1.85 (2H, m, CHC$H_2$), 2.39 (3H, s, S-C$H_3$), 4.27 (1H, dd, *J* 8.4, 6.2 Hz, HetAr-C*H*C), 6.98 (1H, d, *J* 5.3 Hz, HetAr-*H*), 7.19 (1H, d, *J* 5.3 Hz, HetAr-*H*); $\delta_C$ (125 MHz, $CDCl_3$) 0.0 (3 × $CH_3$), 13.7 ($CH_3$), 19.2 ($CH_3$), 20.4 ($CH_2$), 32.3 (CH), 40.4 ($CH_2$), 86.6 (C), 107.8 (C), 123.1 (ArCH), 128.5 (ArC), 129.7 (ArCH), 144.5 (ArC); *m/z* (GCMS) 282.1; (measured 282.0913 $C_{14}H_{22}S_2Si_1$ requires 282.0927).

**General Procedure D: Propargylation of Aryl Sulfoxides**

**Trimethyl(3(2-(methylsulfanyl)phenyl)hex-1-yn-1yl)silane 3au**

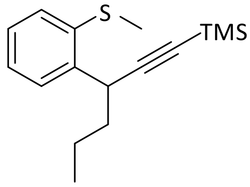

To an oven dried tube flushed with $N_2$, was added methyl phenyl sulfoxide (70.0 mg, 0.5 mmol) and 1,3-*bis*(trimethylsilyl)-1-hexyne (169 mg, 0.75 mol) in MeCN (3 mL). Triflic anhydride (12.6 µL, 0.75 mmol) and 2,6-lutidine (14.5 µL, 1.25 mmol) were then added sequentially at room temperature and the reaction mixture was heated for 18 hours at 60 °C. After cooling to room temperature, the solution was quenched with aqueous saturated $NaHCO_3$ (6 mL) and the aqueous layer was extracted with EtOAc (3 x 5 mL). The combined organic layer was washed successively with aqueous HCl 1.0 M (5ml) and brine (5ml), dried ($MgSO_4$) and concentrated *in vacuo*. The crude product was purified by column chromatography on silica gel eluting with 1% EtOAc in *n*-hexane to yield **3au** (0.072 g, 52 %) as a yellow oil; $\nu_{max}$ (neat)/cm$^{-1}$ 2957, 2872, 2168, 1588, 1465, 1438, 1248, 1111, 1040, 957, 908, 868, 838, 756, 733, 697, 651, 567; $\delta_H$ (400 MHz, $CDCl_3$) 0.21 (9H, s, Si(C*H*$_3$)$_3$), 0.97 (3H, t, J 7.3, C*H*$_3$), 1.49-1.75 (4H, m, 2 x C*H*$_2$), 2.48 (3H, s, SC*H*$_3$), 4.24 (1H, dd, J 8.9, 5, C*H*-C), 7.18-7.25 (3H, m, Ar-*H*), 7.59-7.62 (1H, m, Ar-*H*); $\delta_C$ (125 MHz) 0.1 (Si(*C*H$_3$)$_3$), 13.7 (CH$_2$CH$_2$*C*H$_3$), 16.7 (CH$_2$*C*H$_2$CH$_3$), 20.6 (*C*H$_2$CH$_2$CH$_3$), 35.3 (*C*HCH$_2$), 39.3 (S*C*H$_3$), 86.7 (C*C*-Si), 108.5 (*C*C-Si), 125.6 (Ar-*C*H), 126.9 (Ar-*C*H), 127.2 (Ar-*C*H), 127.8 (Ar-*C*H), 135.9 (Ar-*C*), 140.9 (Ar-*C*); *m/z* (GCMS) 276.1; (measured: 276.1356, $C_{16}H_{24}S_1Si_1$ requires 276.1368).

**Trimethyl(3-(2-methylsulfanyl)pent-1-yn-yl)silane 3av**

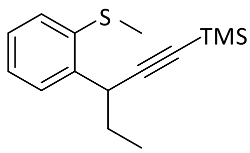

As described in general procedure **D**, methyl phenyl sulfoxide (323 mg, 2.3 mmol), 1,3-*bis*(trimethylsilyl)-1-pentyne (538 mg, 3.4 mmol), triflic anhydride (580 µL, 3.4 mmol), 2,6-lutidine (670 µL, 5.7 mmol) and MeCN (14 mL) were heated at 60 °C for 18 hours. Purification by column chromatography on silica gel eluting with 1% EtOAc in *n*-hexane gave **3av** (0.317 g, 52 %) as a yellow oil; $\nu_{max}$ (neat)/cm$^{-1}$ 2961, 2168, 1464, 1438, 1248, 1038, 984, 838, 746, 697, 650; $\delta_H$ (400 MHz, $CDCl_3$) 0.20 (9H, s, Si(C*H*$_3$)$_3$), 1.05 (3H, t, J = 7.3, C*H*$_3$), 1.6 -

1.84 (2H, m, C$H_2$), 2.47 (3H, s, S-C$H_3$), 4.16 (1H, dd, J 8.7, 4.9, C*H*), 7.17-7.25 (3H, m, Ar-*H*), 7.59 (1H, m, Ar-*H*); $\delta_C$ (125 MHz) 0.4 (Si(C$H_3$)$_3$), 12.0 ($CH_3$), 17.0 ($CH_2$), 30.5 (*C*H), 37.4 (S$CH_3$), 87.3 (Si-*C*C), 108.6 (Si-C*C*), 125.8 (Ar-*C*H), 127.2 (Ar-*C*H), 127.5 (Ar-*C*H), 128.2 (Ar-*C*H), 136.3 (Ar-*C*), 140.8 (Ar-*C*); *m/z/* (GCMS) 262.1; (measured 262.1200, $C_{15}H_{22}S_1Si_1$ requires 262.1211).

**Trimethyl(4-methyl-3-(2-(methylsulfanyl)phenyl)pent-1-yn-1-yl)silane 3aw**

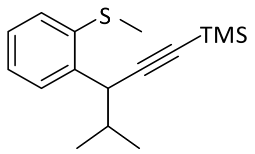


As described in general procedure **D**, methyl phenyl sulfoxide (70 mg, 0.5 mmol), 1,3-*bis*(trimethylsilyl)-4-methyl-1-pentyne (170 mg, 0.75 mmol), triflic anhydride (12.6 µL, 0.75 mmol), 2,6-lutidine (14.5 µL, 1.25 mmol) and MeCN (3 mL) were heated at 60 °C for 18 hours. Purification by column chromatography on silica gel eluting with 1% EtOAc in *n*-hexane gave **3aw** (0.084 g, 61 %) as a yellow oil; $\nu_{max}$ (neat)/cm$^{-1}$ 2959, 2870, 2169, 1587, 1465, 1438, 1383, 1366, 1318, 1248, 1135, 1040, 1009, 908, 838, 744, 698, 678, 630, 615, 561; $\delta_H$ (400 MHz, $CDCl_3$) 0.21 (9H, s, Si(C$H_3$)$_3$), 0.90 (3H, d, J 6.7, C$H_3$), 1.08 (3H, d, J 6.7, C$H_3$), 1.96-2.04 (1H, m, C*H*($CH_3$)$_2$), 2.47 (3H, s, SC$H_3$), 4.16 (1H, d, J 4.8, C*H*-C), 7.17-7.27 (3H, m, Ar-*H*), 7.56 (1H, dd, J 7.4, 1.5, Ar-*H*); $\delta_C$ (125 MHz) -0.1 (Si(C$H_3$)$_3$), 16.4 ($CH_3$), 17.1 ($CH_3$), 21.1 (*C*H($CH_3$)$_2$), 32.5 (*C*H), 42.2 (S$CH_3$), 87.8 (*C*C-Si), 106.2 (C*C*-Si), 124.8 (Ar-*C*H), 126.5 (Ar-*C*H), 126.8 (Ar-*C*H), 128.4 (Ar-*C*H), 136.1 (Ar-*C*), 139.4 (Ar-*C*); *m/z* (GCMS) 276.1; (measured 276.1356, $C_{16}H_{24}S_1Si_1$ requires 276.1368).

**(3-Cyclopentyl-3-(2-(methylsulfanyl)phenyl)prop-1-yn-1-yl)trimethylsilane 3ax**

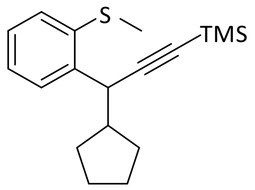


As described in general procedure **D**, methyl phenyl sulfoxide (70 mg, 0.5 mmol), 1,3-*bis*(trimethylsilyl)-3-cyclopentyl-1-propyne (189 mg, 0.75 mmol), triflic anhydride (12.5 µL, 0.75 mmol), 2,6-lutidine (14.5 µL, 1.25 mmol) and MeCN (3 mL) were heated at 60 °C for 18

hours. Purification by column chromatography on silica gel eluting with 1% EtOAc in *n*-hexane gave **3ax** (0.087 g, 57 %) as a yellow oil; $\nu_{max}$ (neat)/cm$^{-1}$ 2954, 2865, 2168, 1587, 1465, 1438, 1247, 1042, 941, 838, 757, 697, 635; $\delta_H$ (400 MHz, $CDCl_3$) 0.20 (9H, s, Si($CH_3$)$_3$), 1.46-1.70 (9H, m, cyclopentyl H), 2.47 (3H, s, S$CH_3$), 4.30 (1H, d, J 6.1, C*H*), 7.17-7.25 (3H, m, Ar-*H*), 7.58 (1H, dd, J 7.4, 1.6, Ar-*H*); $\delta_C$ (125 MHz) 0.5 (Si($CH_3$)$_3$), 17.1 (cyclopentyl-$CH_2$), 25.5 (cyclopentyl-$CH_2$), 25.7 (cyclopentyl-$CH_2$), 29.1 (cyclopentyl-$CH_2$), 31.1 (cyclopentyl-*C*H), 39.9 (*C*H), 45.5 (S$CH_3$), 87.3 (C*C*-Si), 108.0 (*C*C-Si), 125.7 (Ar-*C*H), 127.1 (Ar-*C*H), 127.4 (Ar-*C*H). 128.6 (Ar-*C*H), 136.3 (Ar-*C*), 140.8 (Ar-*C*); *m/z* (GCMS) 287.1 (-$CH_3$); (measured 287.1277, $C_{17}H_{23}S_1Si_1$ requires 287.1284).

**(3-Cyclohexyl-3-(2-methylsulfanyl)phenyl)prop-1-yn-1-yl)trimethylsilane 3ay**

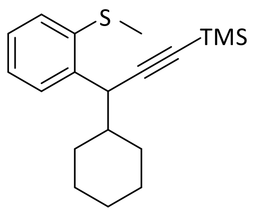


As described in general procedure **D**, methyl phenyl sulfoxide (70 mg, 0.5 mmol), 1,3-*bis*(trimethylsilyl)-3-cyclohexyl-1-propyne (199 mg, 0.75 mmol), triflic anhydride (12.6 µL, 0.75 mmol), 2,6-lutidine (14.5 µL, 1.25 mmol) and MeCN (3 mL) were heated at 60 °C for 18 hours. Purification by column chromatography on silica gel eluting with 1% EtOAc in *n*-hexane gave **3ay** (0.120g, 63%) as a yellow oil; $\nu_{max}$ (neat)/cm$^{-1}$ 2923, 2851, 2168, 1587, 1464, 1442, 1317, 1247, 1061, 1042, 996, 948, 878, 838, 752, 734, 699, 661, 639, 627, 584, 543; $\delta_H$ (500 MHz, $CDCl_3$) 0.20 (9H, s, Si($CH_3$)$_3$), 1.10-1.37 (5H, m, cyclohexyl), 1.58- 1.75 (6H, m, cyclohexyl), 2.47 (3H, s, S$CH_3$), 4.13 (1H, d, J 5.2, Ph-C*H*C), 7.16-7.27 (3H, m, Ar-*H*), 7.53 (1H, d, J 7.5, Ar-*H*); $\delta_C$ (125 MHz) 0.05 (($CH_3$)$_3$Si), 16.5 (cyclohexyl $CH_2$), 25.9 (cyclohexyl $CH_2$), 26.1 (cyclohexyl $CH_2$), 26.2 (cyclohexyl $CH_2$), 28.0 (cyclohexyl $CH_2$), 31.4 (cyclohexyl CH), 41.6 (Ph-*CH*), 42.3 (S-$CH_3$), 87.4 (Si-*C*C-C), 107.1 (Si-C*C*-C), 124.8 (Ar-*C*H), 126.6 (Ar-*C*H), 126.8 (Ar-*C*H), 128.6 (Ar-*C*H), 136.25 (Ar-*C*), 139.2 (Ar-*C*); *m/z* (GCMS) 316.2; (measured 316.1680, $C_{19}H_{28}S_1Si_1$ requires 316.1675).

**(2-(Hex-3-yn-2-yl)phenyl)(methyl)sulfide 3az**

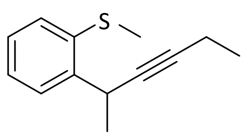


As described in general procedure **D**, methyl phenyl sulfoxide (70 mg, 0.5 mmol), 2-trimethylsilyl-3-hexyne (115 mg, 0.75 mmol), triflic anhydride (12.5 µL, 0.75 mmol), 2,6-lutidine (14.5 µL, 1.25 mmol) and MeCN (3 mL) were heated at 60 °C for 18 hours. Purification by column chromatography on silica gel eluting with 1% EtOAc in *n*-hexane gave **3az** (0.054 g, 53 %) as a yellow oil; $\nu_{max}$ (neat)/cm$^{-1}$ 3058, 2973, 2920, 1715, 1587, 1568, 1467, 1437, 1368, 1318, 1257, 1189, 1154, 1042, 967, 686, 608, 560; $\delta_H$ (400 MHz, $CDCl_3$) 1.16 (3H, t, J 7.6, C*H*$_3$CH$_2$), 1.43 (3H, d, J 7.1, C*H*$_3$CH), 2.24 (2H, qd, J 7.5, 2.3, CH$_3$C*H*$_2$), 2.48 (3H, s, SC*H*$_3$), 4.23 (1H, qt, J 7.0, 2.2, CH$_3$C*H*), 7.17-7.26 (3H, m, Ar-*H*), 7.62 (1H, m, Ar-*H*); $\delta_C$ (125 MHz) 12.5 (CH$_2$*C*H$_3$), 14.2 (CH*C*H$_3$), 16.6 (*C*H$_2$CH$_3$), 23.5 (*C*HCH$_3$), 28.9 (S*C*H$_3$), 82.2 (*C*C-CH$_2$CH$_3$), 83.2 (C*C*-CH$_2$CH$_3$), 125.7 (Ar-*C*H), 126.7 (Ar-*C*H), 127.14 (Ar-*C*H), 127.1 (Ar-*C*H), 135.6 (Ar-*C*), 142.6 (Ar-*C*); *m/z* (GCMS) 204.1; (measured 203.0891, $C_{13}H_{25}S_1$ requires 203.0889).

**Methyl(2-(oct-4-yn-3-yl)phenyl)sulfide 3ba**

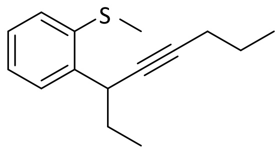


As described in general procedure **D**, methyl phenyl sulfoxide (70 mg, 0.5 mmol), 3-trimethylsilyl-4-octyne (136 mg, 0.75 mmol), triflic anhydride (12.5 µL, 0.75 mmol), 2,6-lutidine (14.5 µL, 1.25 mmol) and MeCN (3 mL) were heated at 60 °C for 18 hours. Purification by column chromatography on silica gel eluting with 1% EtOAc in *n*-hexane gave **3aaa** (0.046 g, 45 %) as a yellow oil; $\nu_{max}$ (neat)/cm$^{-1}$ 2961, 2929, 2870, 1587, 1438, 1377, 1338, 1276, 1061, 1042, 967, 906, 816, 692, 625; $\delta_H$ (400 MHz, $CDCl_3$) 1.01-1.07 (6H, m, 2 x C*H*$_3$), 1.53- 1.84 (4H, m, 2 x C*H*$_2$), 2.23 (2H, td, J 7, 2.2, C*H*$_2$), 2.47 (3H, s, SC*H*$_3$), 4.10 (1H, m, Ph-C*H*), 7.17-7.26 (3H, m, Ar-*H*), 7.60 (1H, dd, J 7.5, 5.9, Ar-*H*); $\delta_C$ (125 MHz) 11.5 (*C*H$_3$(CH$_2$)$_2$), 13.2 (*C*H$_3$CH$_2$CH), 16.3 (CH$_3$*C*H$_2$CH$_2$), 20.5 (CH$_3$*C*H$_2$CH), 22.2 (CH$_3$CH$_2$*C*H$_2$), 30.1 (CH$_3$CH$_2$*C*H), 35.8 (S-*C*H$_3$), 81.3 (CH$_2$*C*C), 82.4 (C*C*-CH), 125.1 (Ar-*C*H), 126.3 (Ar-*C*H), 126.7

(Ar-*C*H), 127.5 (Ar-*C*H), 135.62 (Ar-*C*), 141.1 (Ar-*C*); *m/z* (GCMS) 232.1; (measured 232.1276, $C_{15}H_{20}S_1$ requires 232.1280).

**Iterative, selective *ortho*-propargylation of 4-methoxyphenylmethyl sulphide**

**2-(Hept-2-yn-1-yl)-4-methoxy-1-(methylsulfinyl)benzene 1al**

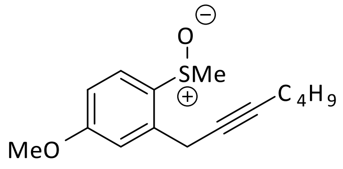


To a solution of (2-(Hept-2-ynyl)-4-methoxyphenyl)(methyl)sulfide (0.186 g, 0.75 mmol) in $CH_2Cl_2$ (10 mL) was added $NaHCO_3$ (0.069 g, 0.825 mmol) followed by a solution of *m*-CPBA (0.188 g, 0.825 mmol) in $CH_2Cl_2$ (5 mL) at 0 °C. The reaction mixture was then allowed to warm to room temperature for two hours before quenching with aqueous saturated $NaHCO_3$ (10 mL). The aqueous layer was then extracted with $CH_2Cl_2$ (3 x 10 mL) and the combined organic layers were washed with brine (20 mL), dried ($MgSO_4$) and concentrated in vacuo. The crude product was purified by column chromatography on silica gel eluting with 10% EtOAc in $CH_2Cl_2$ to yield the product (0.182 g, 92% yield) as a yellow oil; $\nu_{max}$ (neat)/cm$^{-1}$ 2956, 2930, 1594, 1455, 1322, 1282, 1159, 1084, 732; $\delta_H$ (400 MHz, $CDCl_3$) 0.90 (3H, t, *J* 7.2, *CH₃*), 1.51 - 1.34 (4H, m, 2 × *CH₂*), 2.17 (2H, tt, *J* 2.4, 7.2, C*CH₂*), 2.74 (3H, s, S*CH₃*), 3.61 (2H, t, *J* 2.4, Ph-*CH₂*C), 3.85 (3H, s, O*CH₃*), 7.02-6.99 (2H, m, Ar-H) 7.91 (1H, m, ArC*H*); $\delta_C$ (100 MHz, $CDCl_3$) 13.7 (*C*H₃), 18.6 (C*C*H₂), 22.1 (*C*H₂), 22.2 (Ph*C*H₂), 31.0 (*C*H₂), 43.4 (S*C*H₃), 55.6 (O*C*H₃), 76.4 (*CC*), 84.2 (*CC*), 114.0 (Ar*C*H), 115.1 (Ar*C*H), 125.7 (Ar*C*H), 134.8 (Ar*C*), 136.6 (Ar*C*), 162.2 (Ar*C*); *m/z* (ES+) (M + H), 265; (Found: M + H, 265.1259. $C_{15}H_{21}O_2S$ requires M + H, 265.1257).

**(2-(3-Cyclohexylprop-2-yn-1-yl)-6-(hept-2-yn-1-yl)-4-methoxyphenyl)(methyl)sulfane 3bb**

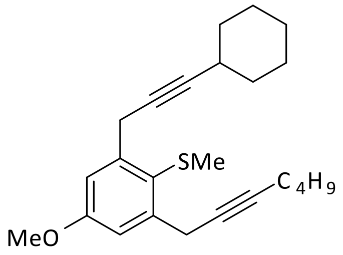

An oven-dried microwave tube was flushed with $N_2$ before adding a solution containing 2-(hept-2-yn-1-yl)-4-methoxy-1-(methylsulfinyl)benzene (132 mg, 0.50 mmol) and (3-cyclohexylprop-2-ynyl)trimethylsilane (146 mg, 0.75 mol) in MeCN (2.5 mL). Triflic anhydride (125 µL, 0.75 mmol) and 2,6-lutidine (145 µL, 0.625 mmol) were added sequentially at room temperature and the reaction mixture was then heated in a microwave reactor for 15 min at 130 °C. After cooling to room temperature, the solution was quenched with aqueous saturated $NaHCO_3$ (10 mL) and the aqueous layer was extracted with EtOAc (3 x 10 mL). The combined organic layers were washed successively with aqueous HCl 1.0 M (10 mL) and brine (10 mL), dried ($MgSO_4$) and concentrated *in vacuo*. The crude product was purified by column chromatography on silica gel eluting with 5% $CH_2Cl_2$ in hexane to yield the product (0.136 g, 74% yield) as a clear oil; $\nu_{max}$ (neat)/cm$^{-1}$ 2928, 1594, 1452, 1322, 1283, 1159, 1084; $\delta_H$ (400 MHz, $CDCl_3$) 0.91 (3H, t, *J* 7.2, *$CH_3$*), 1.34 - 1.26 (3H, m, $CH_2$), 1.53 - 1.40 (7H, m, $CH_2$), 1.72-1.69 (2H, m, *$CH_2$*), 1.83-1.80 (2H, m, *$CH_2$*), 2.19 (3H, s, S*$CH_3$*), 2.24-2.22 (2H, m, C*$CH_2$*), 2.46-2.38 (1H, m, *CH*), 3.84 (3H, s, O*$CH_3$*), 3.91-3.88 (4H, m, Ph-*$CH_2$*C), 7.11 (1H, d, *J* 2.8, Ar-H), 7.15 (1H, d, *J* 2.8, Ar-H); $\delta_C$ (100 MHz, $CDCl_3$) 13.8 (*C*$H_3$), 18.7 (C*C*$H_2$), 19.5 (S*C*$H_3$), 22.1 (*C*$H_2$), 25.0 (Ph*C*$H_2$ + *C*$H_2$), 25.1 (C*C*H), 26.1 (Ph*C*$H_2$), 29.4 (*C*$H_2$), 31.3 (*C*$H_2$), 33.2 (*C*$H_2$), 55.3 (O*C*$H_3$), 78.0 (*CC*), 78.1 (*CC*), 83.3 (*CC*), 87.7 (*CC*), 113.2 (Ar*C*H), 113.4 (Ar*C*H), 124.7 (Ar*C*), 144.1 (Ar*C*), 144.2 (Ar*C*), 160.1 (Ar*C*); *m/z* (GCMS) 353.2 ($C_{23}H_{29}O_1S_1$); (measured 369.2253, $C_{24}H_{33}O_1S_1$ requires 369.2252).

**Iodine Mediated Cyclisation**

**1-(3-methylbenzo[b]thiophen-2-yl)propan-1-one 7c**

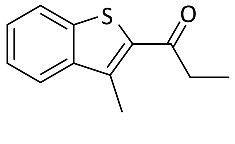

To an oven-dried tube was added (2-Hex-3-yn-2-yl)phenyl)(methyl)sulfide **3az** (20.0 mg, 0.1 mmol), Iodine (17.7 mg, 0.07 mmol) and toluene (2 mL, 0.05 M). Oxygen was bubbled through the reaction for 15 minutes before the mixture was heated to 80 °C for 18 hours. After this period the reaction was allowed to stir for 48 hours at room temperature before it was quenched with sat. sodium thiosulphate solution (5 mL). The aqueous phase was

separated and extracted with $Et_2O$ (3 X 5 mL). The combined organic layers were then washed with brine (5 mL), dried using $Na_2SO_4$ and filtered before the solvent was removed *in vacuo*. Purification by preparative thin layer chromatography eluting with hexane gave **7c** as a white solid (19.5 mg, 0.095 mmol, 95%); mp: 83-85 °C; $\nu_{max}$ (neat/cm$^{-1}$); 3064, 2971, 2918, 2846, 1644, 1558, 1517, 1449, 1408, 1380, 1315, 1262, 1157, 1090, 1040, 977, 874, 814, 729; $\delta_H$ (400 MHz, $CDCl_3$) 1.26 (3 H, t, *J*=7.19 Hz, C$H_3$), 2.78 (3 H, s, C$H_3$), 2.99 (2 H, q, *J*=7.31 Hz, C$H_2$), 7.37 - 7.59 (2 H, m, ArC*H*), 7.76 - 7.97 (2 H, m, ArC*H*); $\delta_C$ (100 MHz, $CDCl_3$) 8.28 (*C*$H_3$), 13.84 (*C*$H_3$), 36.00 (*C*$H_2$), (122.73 (Ar*C*H), 124.08 (Ar*C*H), 124.64 (Ar*C*H), 127.38 (Ar*C*H), 135.08 (Ar*C*), 139.15 (Ar*C*), 139.76 (Ar*C*), 140.49 (Ar*C*), 196.37 (*C*O); *m/z* (GCMS); 204.0; (measured 204.0597, $C_{12}H_{12}O_1S_1$ requires 204.0603).

## $^{1}$H and $^{13}$C NMR

## 3-(Phenylsulfinyl)thiophene 1af

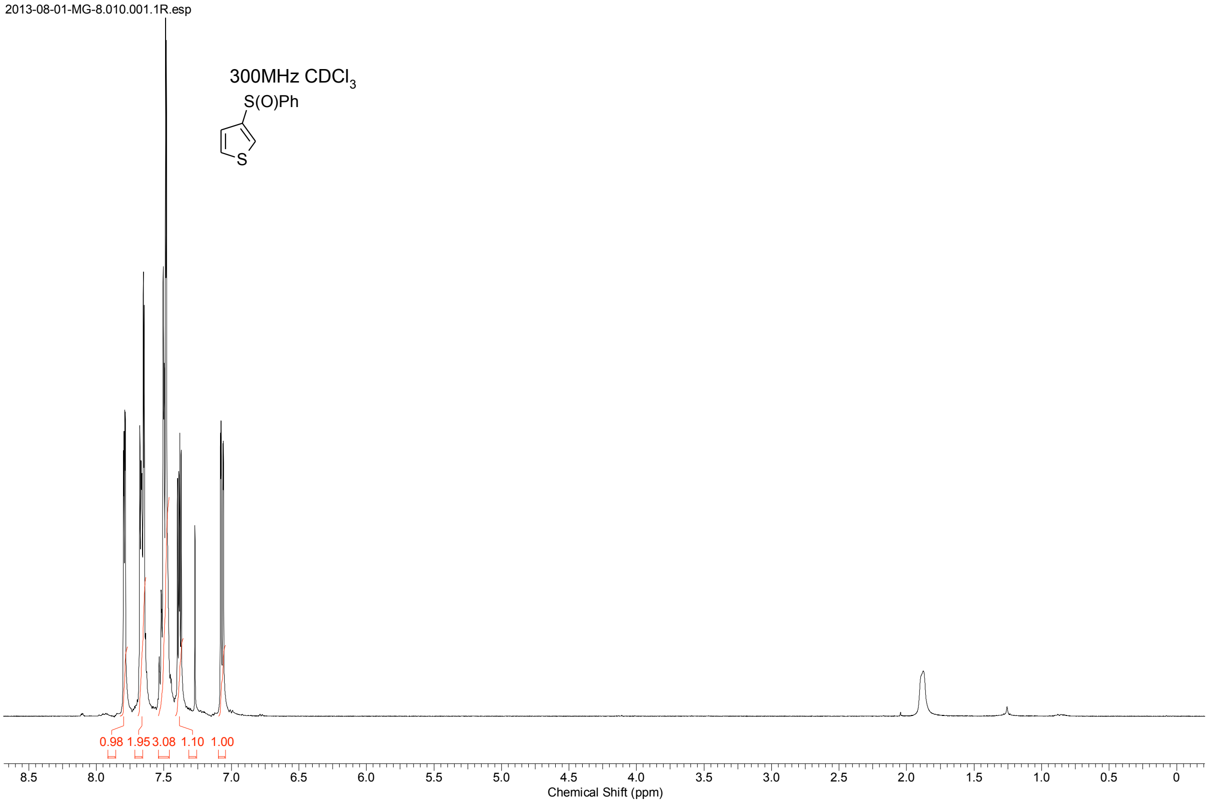


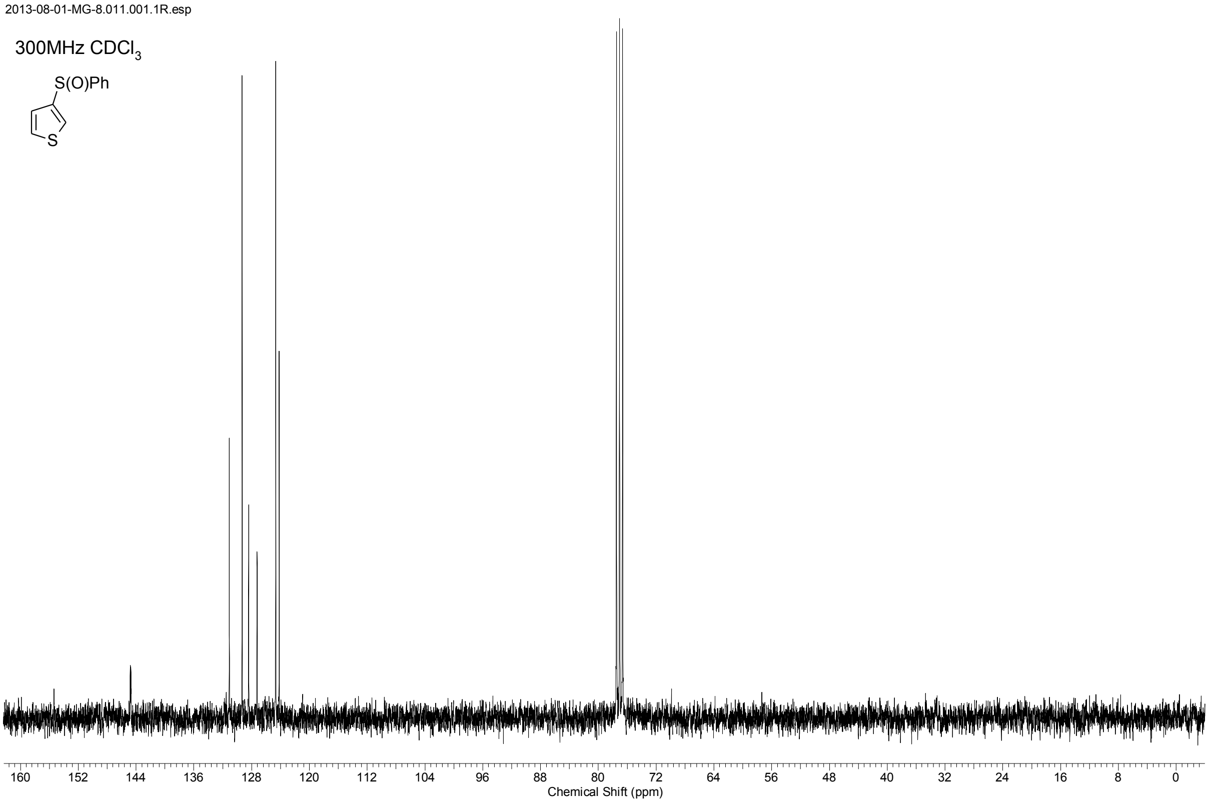

## 3-Bromo-4-(phenylsulfinyl)thiophene 1ag

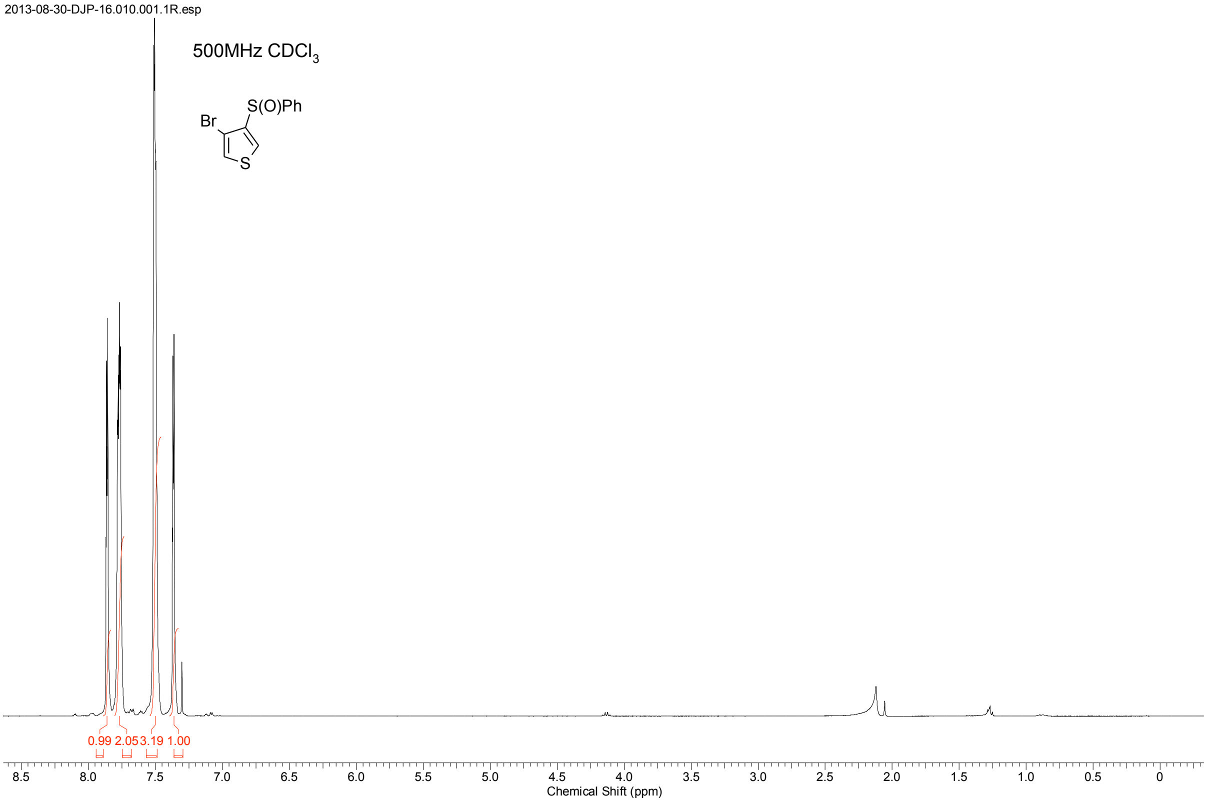


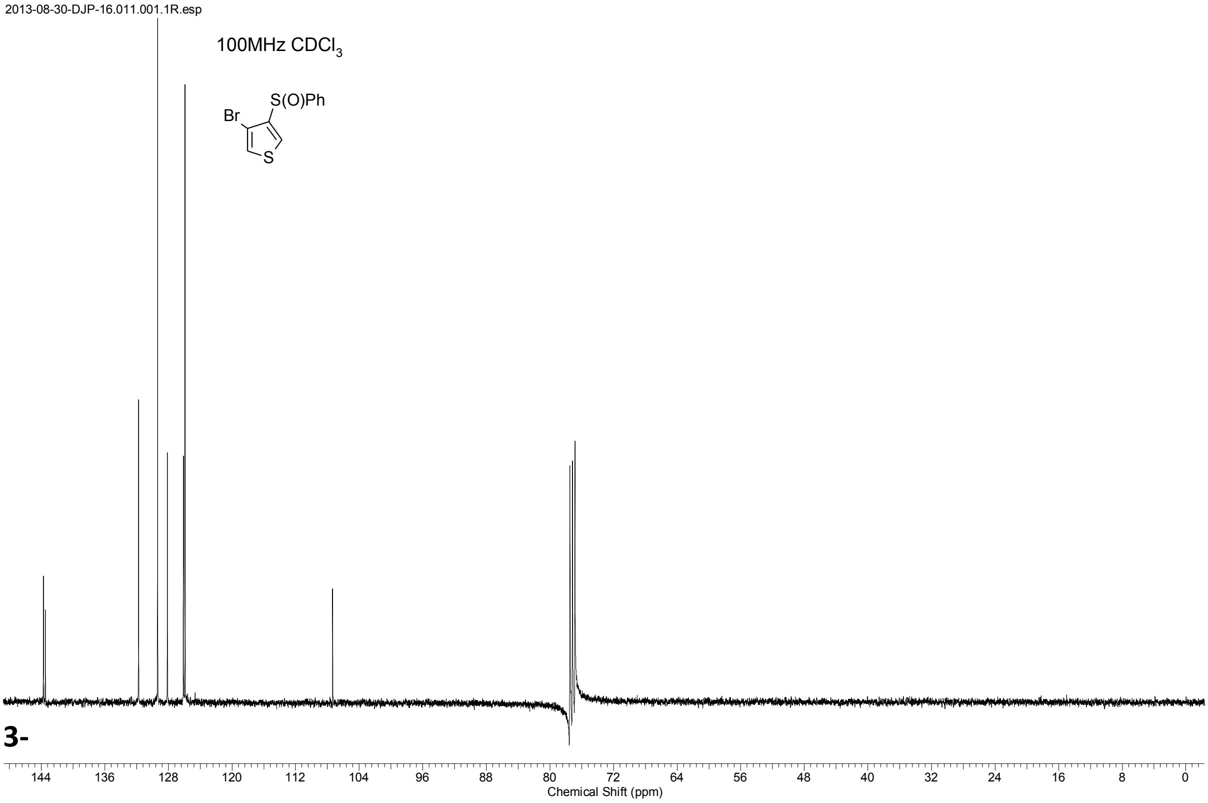


3-

## (Phenylsulfinyl)-4-(phenylthio)thiophene 1ah

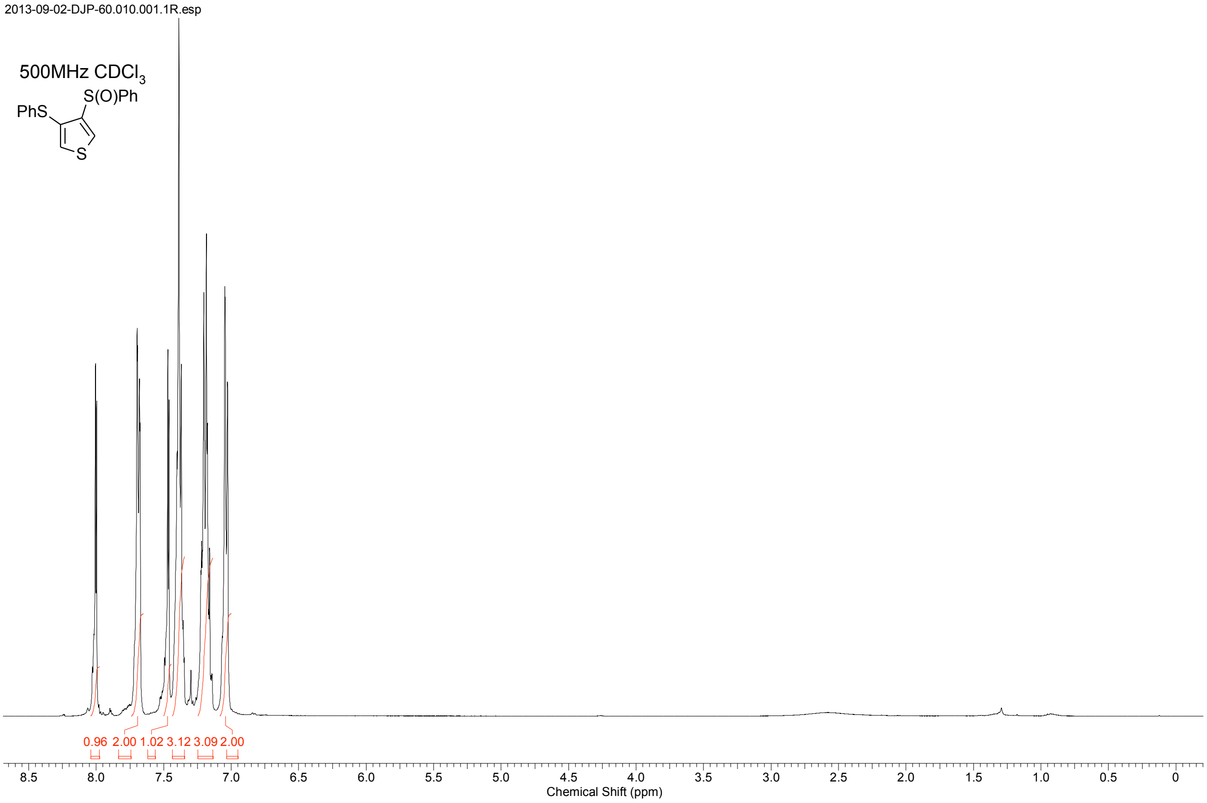


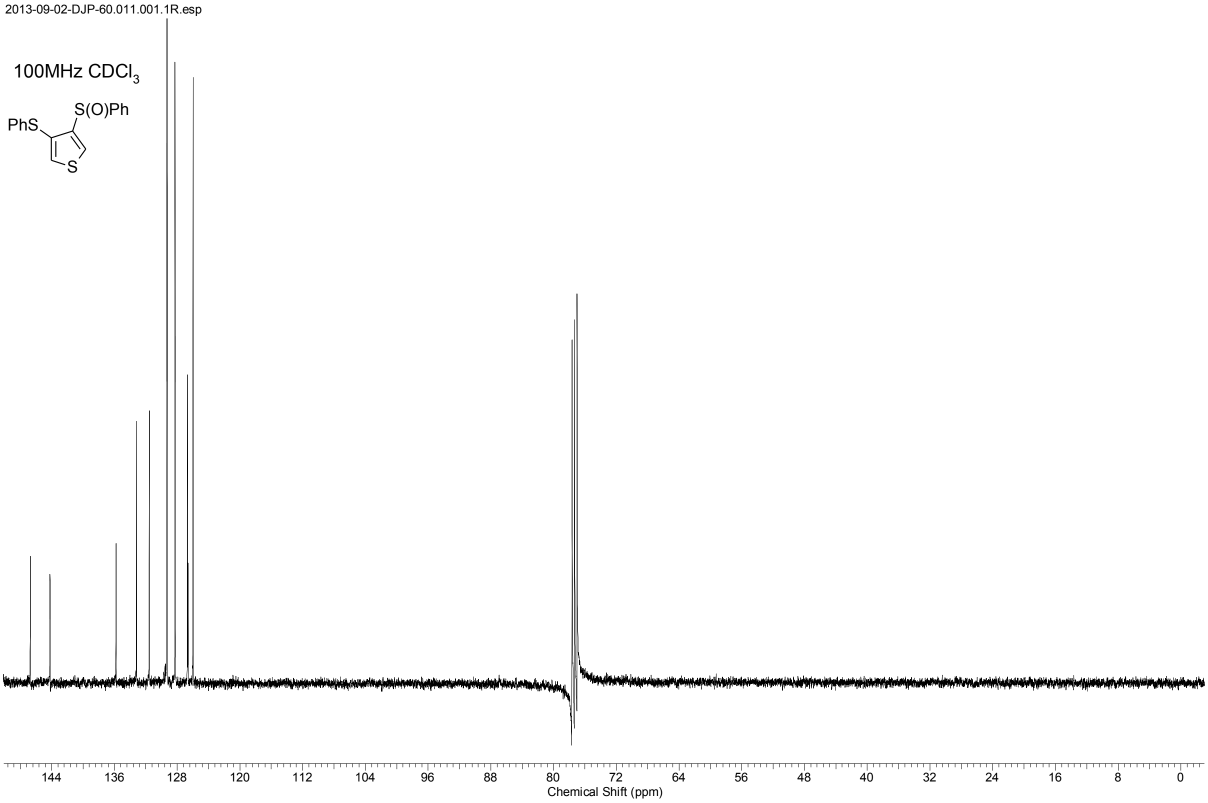

## 3-(Phenylsulfinyl)furan 1aj

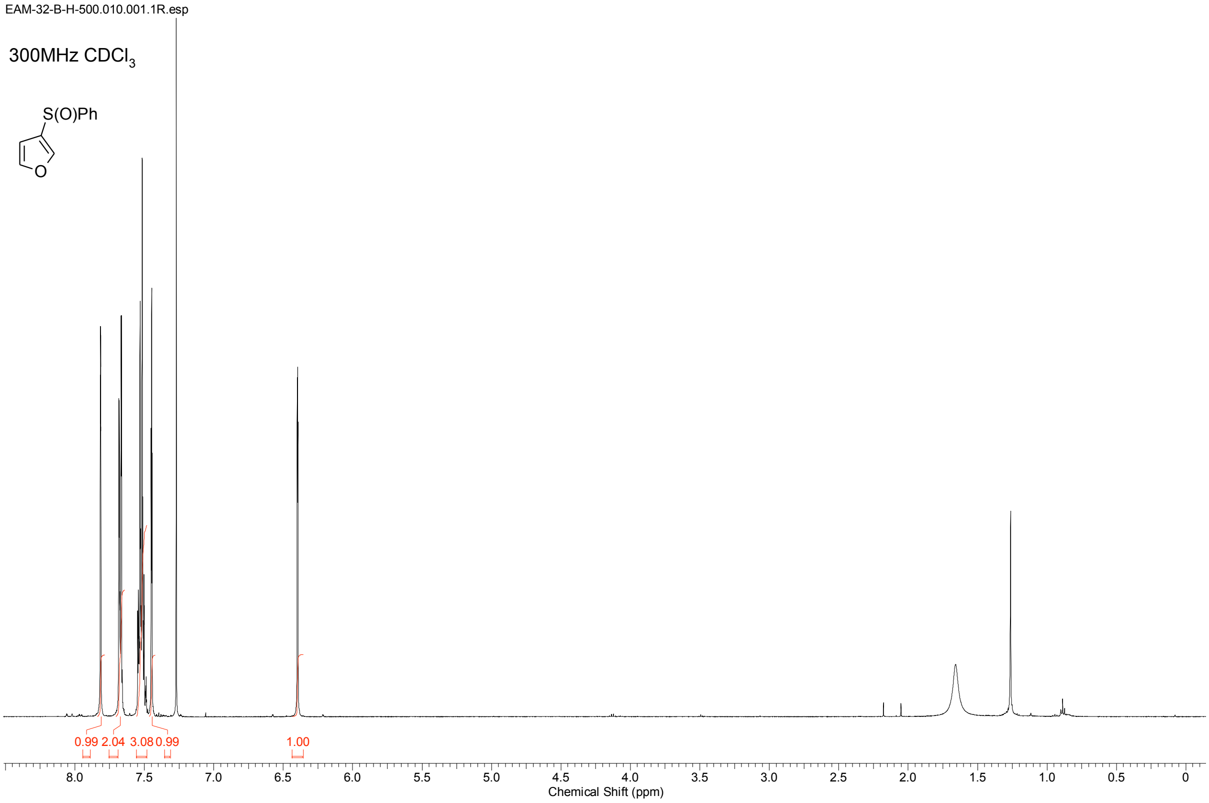


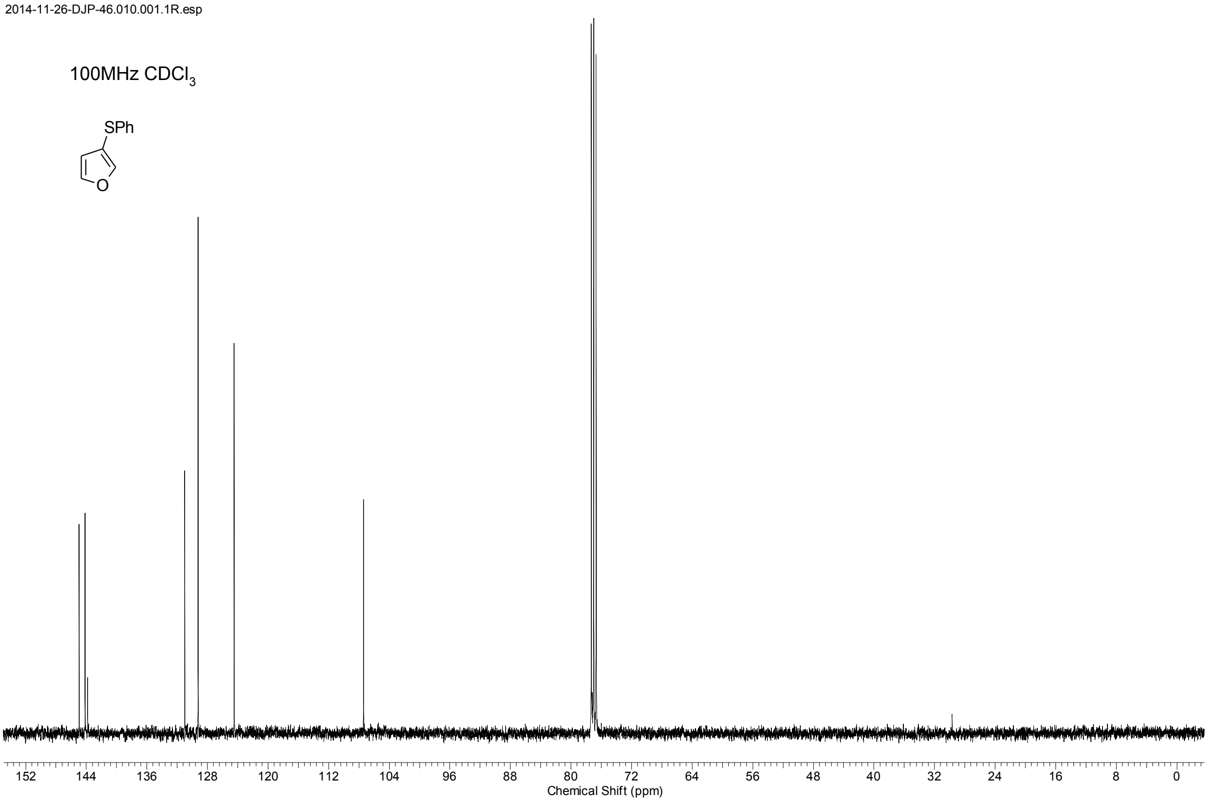

## 3,4-*bis*(phenylsulfinyl)thiophene 1ak

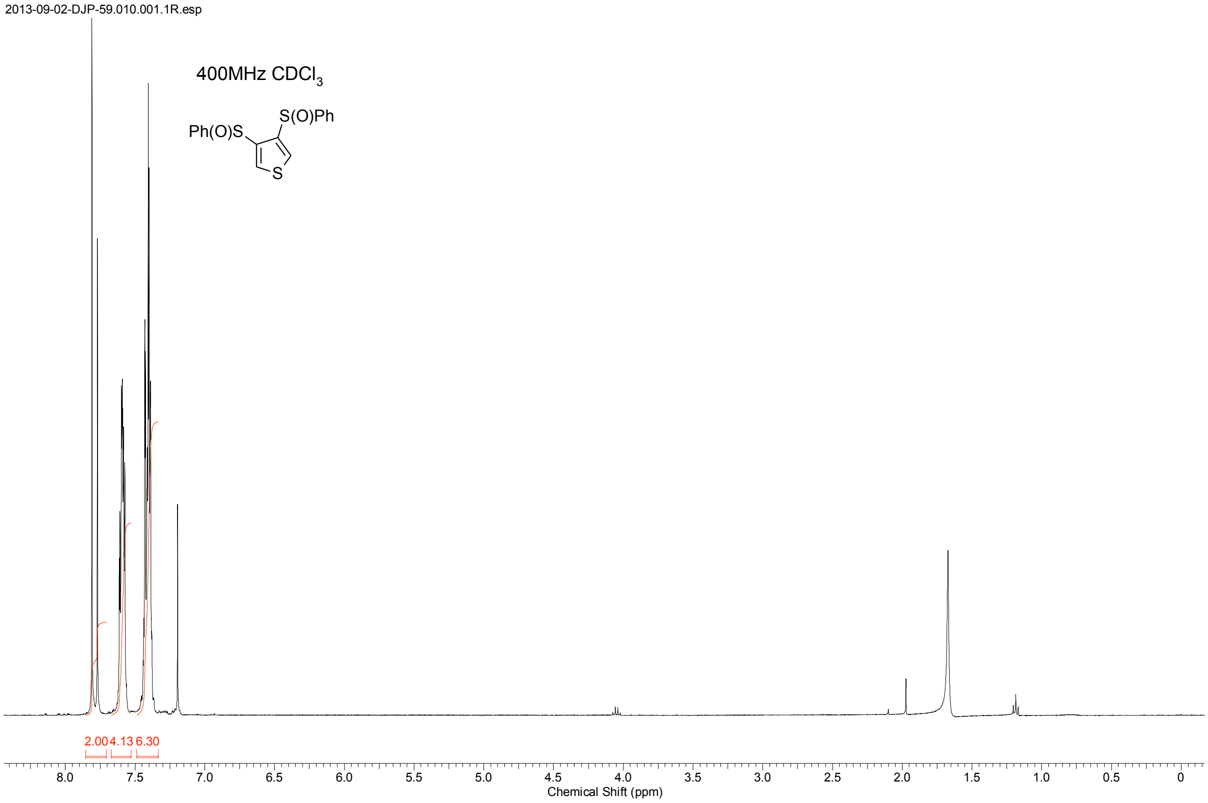


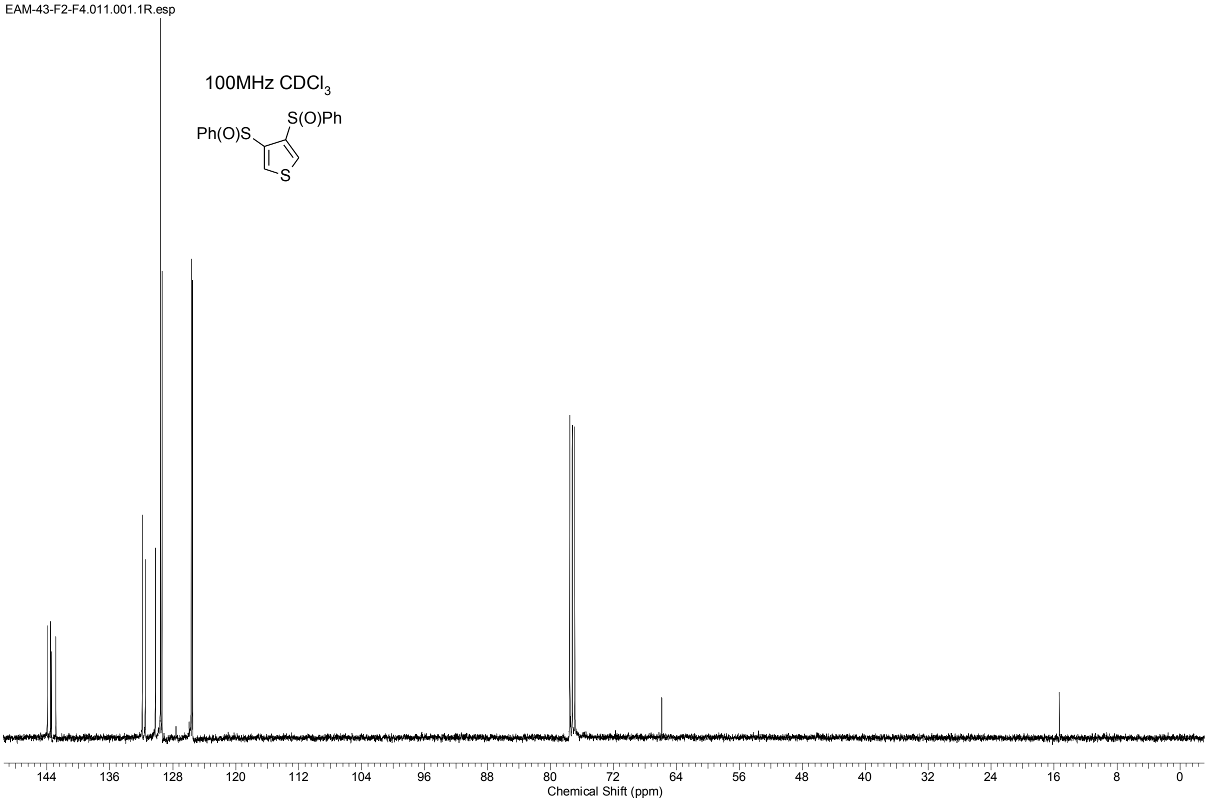

## 2-(Hept-2-yn-1-yl)-3-(methylsulfanyl)thiophene 3ab

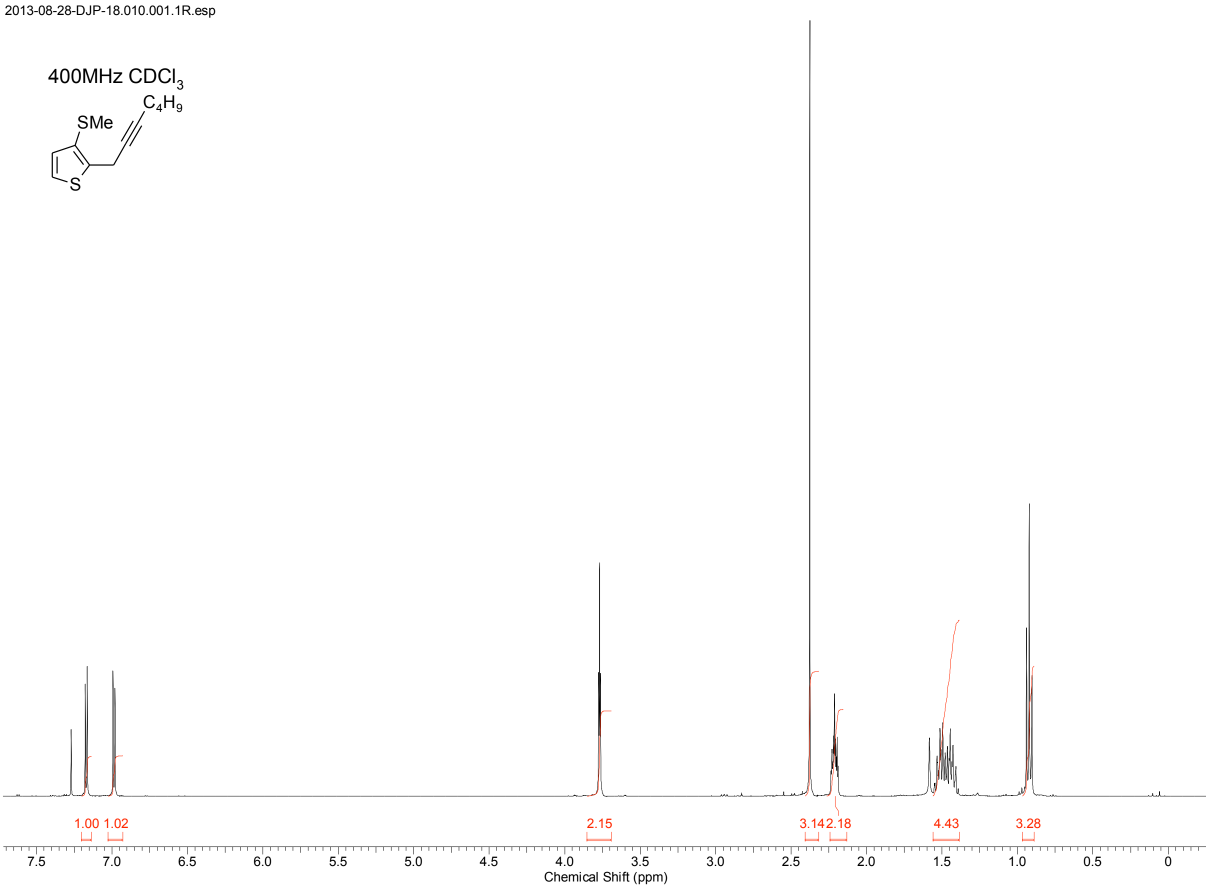


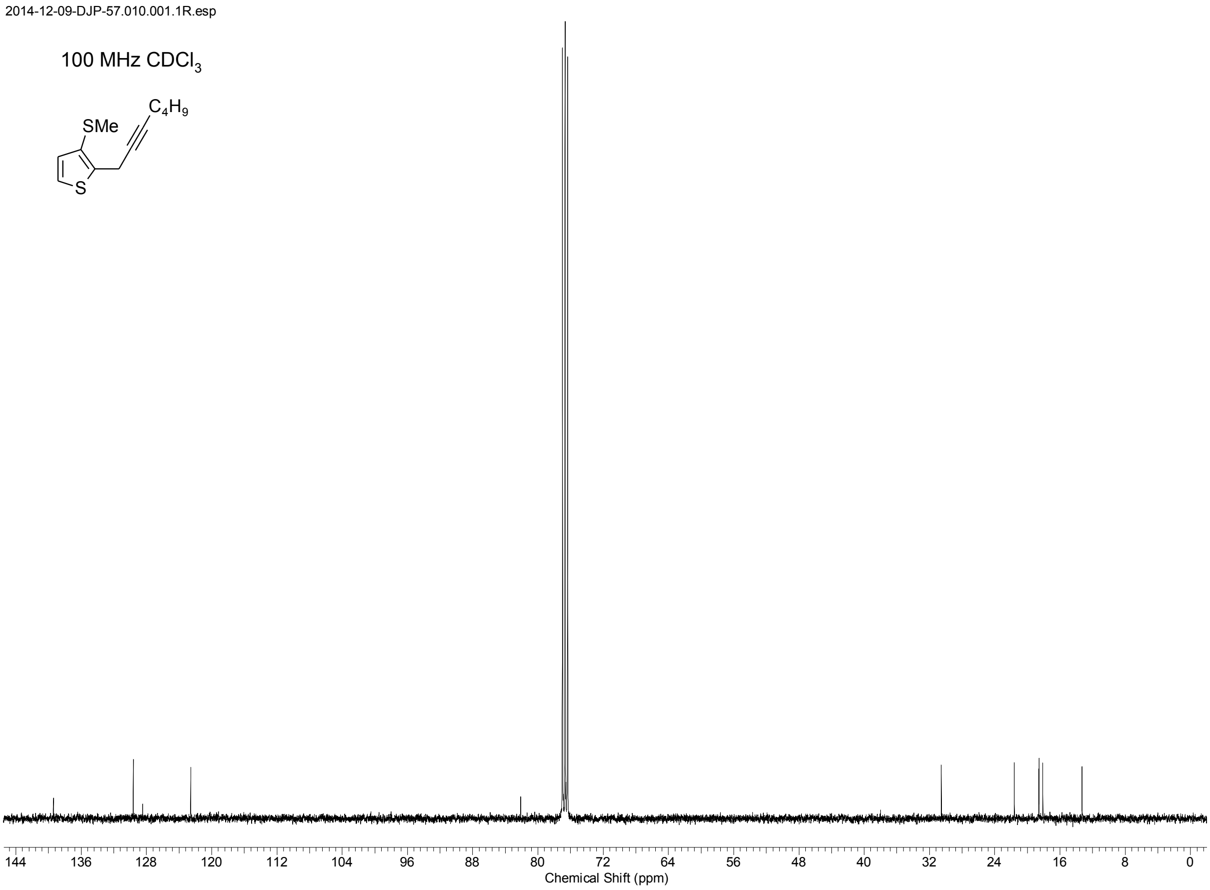

## 3-(Hept-2-yn-1-yl)-2-(methylsulfanyl)thiophene 3ac

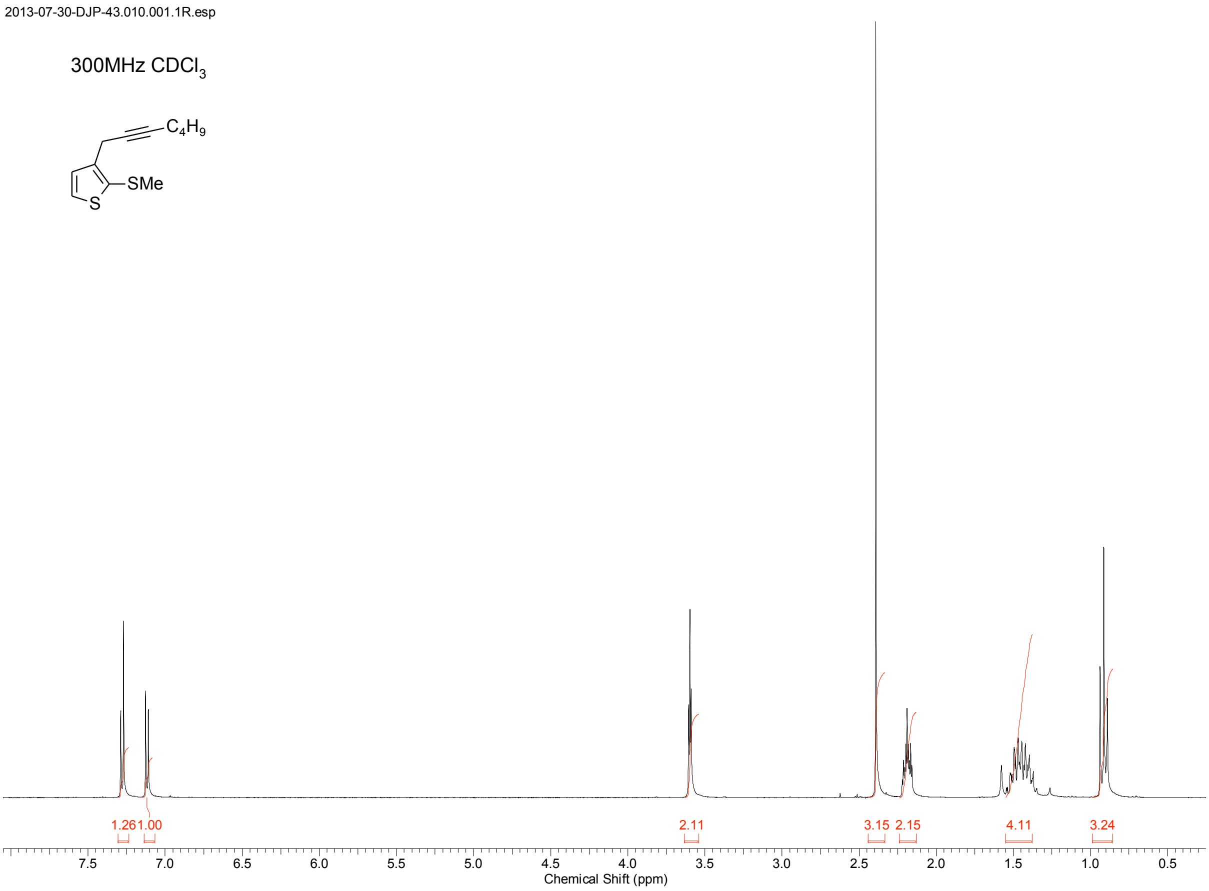


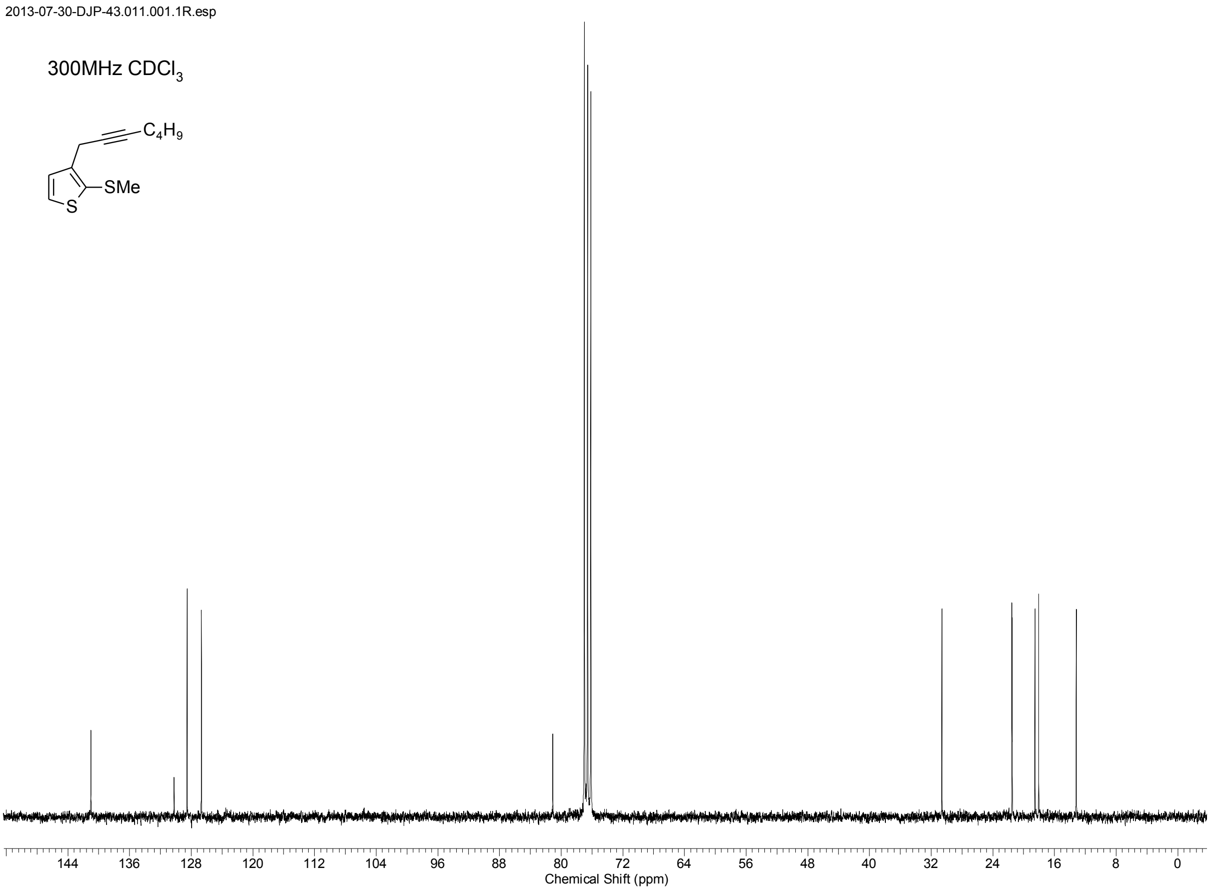

## 2-(Hept-2-yn-1-yl)-3-(methylsulfanyl)furan 3ad

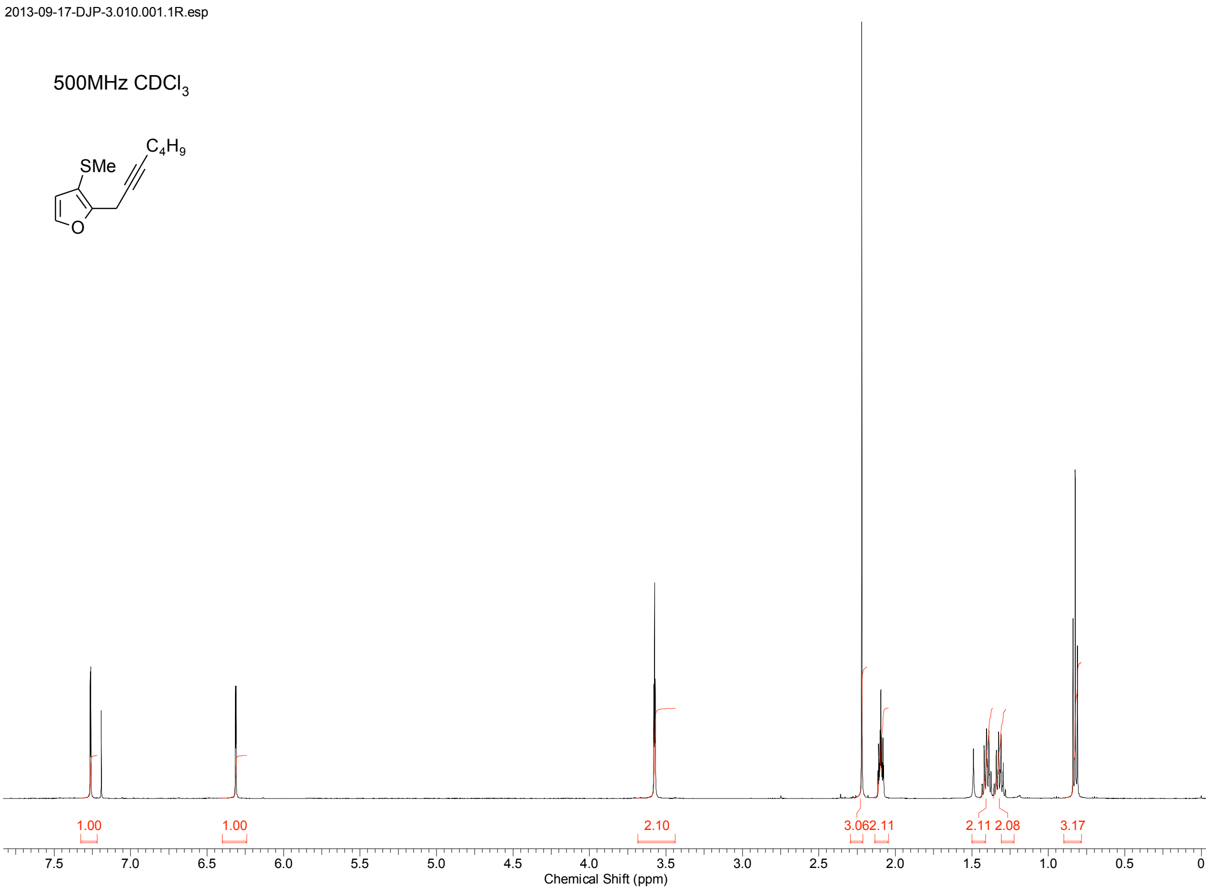


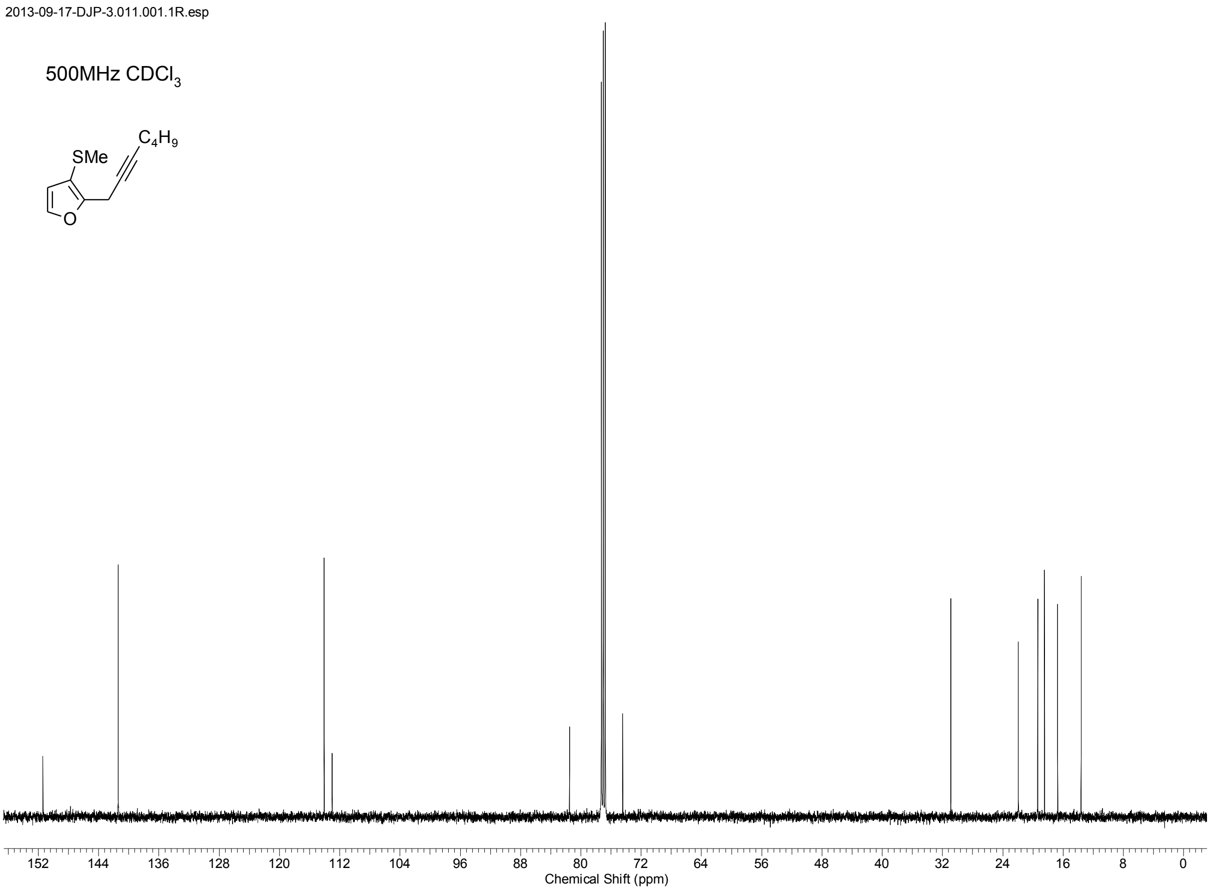

## 3-(Hept-2-yn-1-yl)-2-(methylsulfanyl)furan 3ae

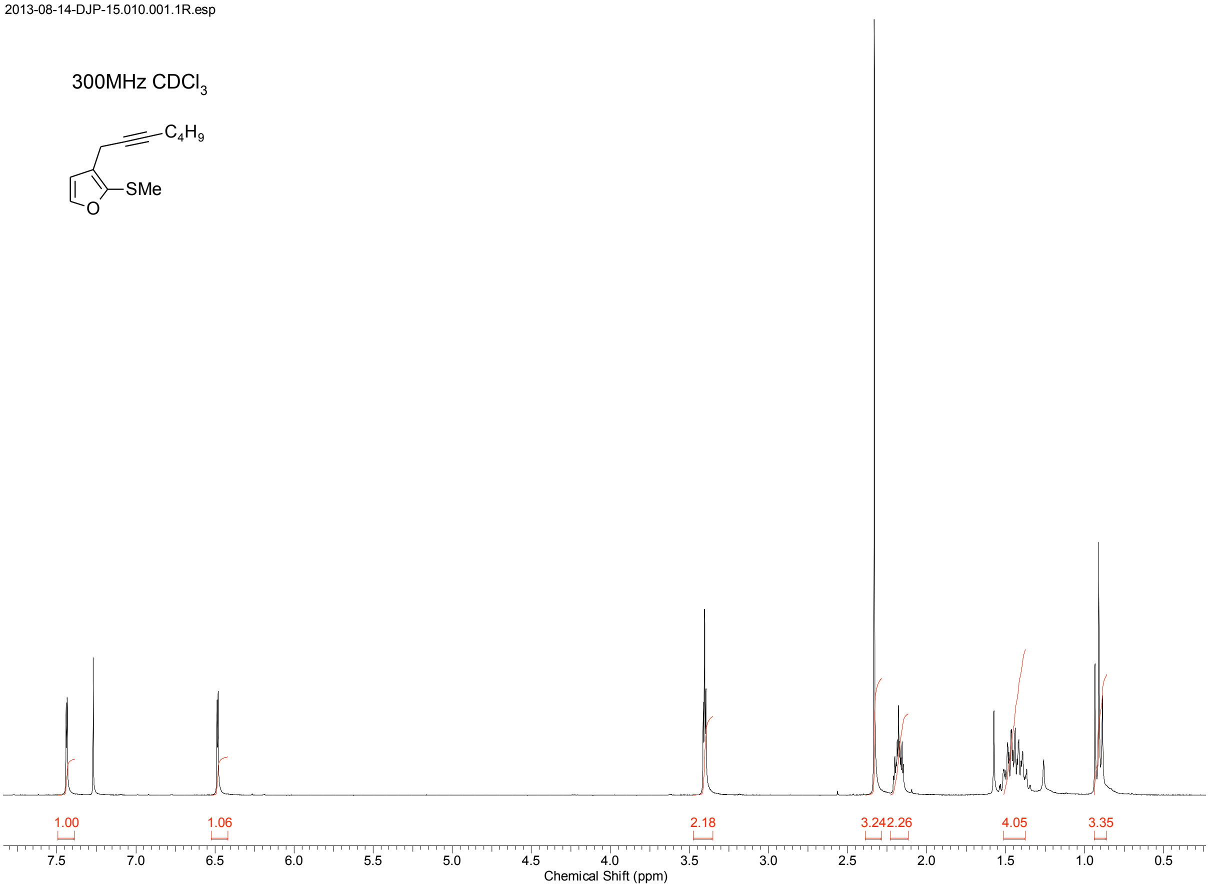


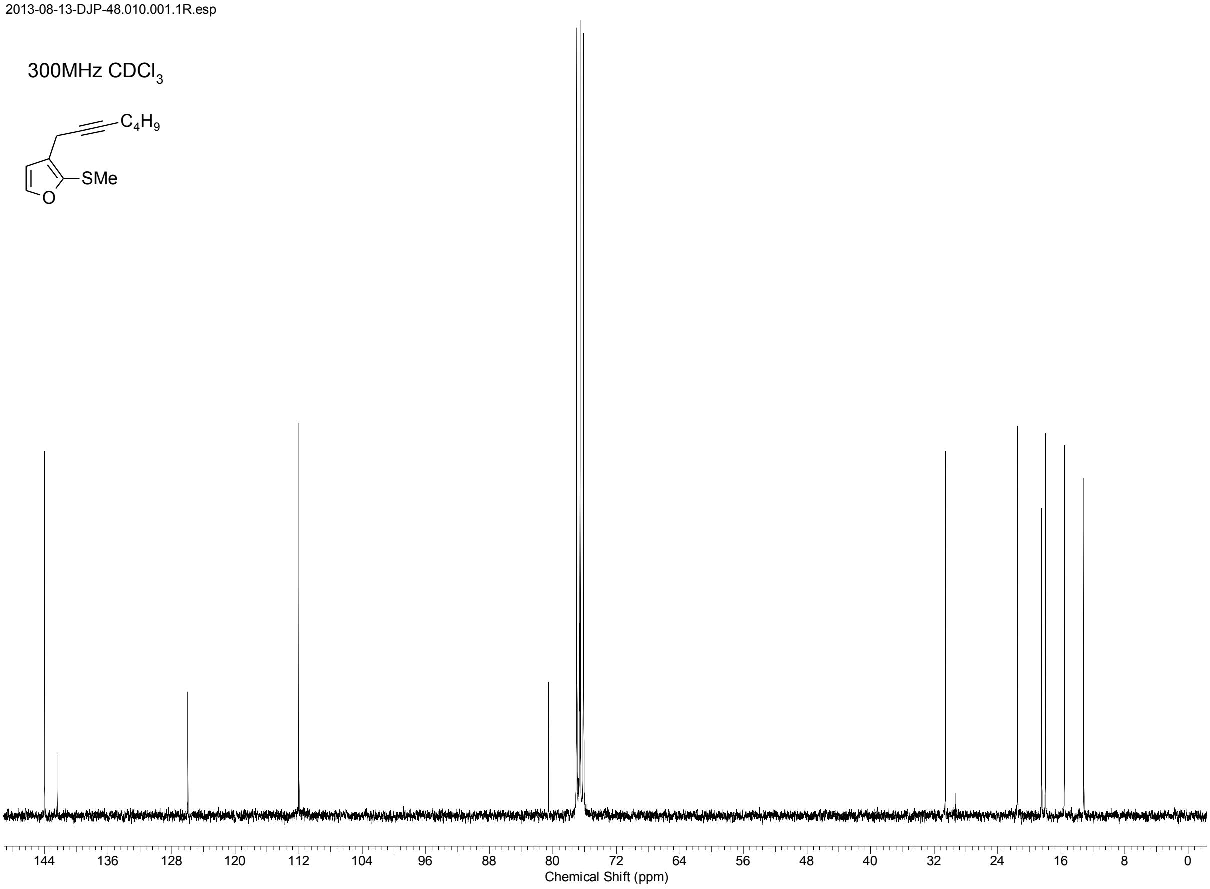

## 2-(Hept-2-yn-1-yl)-3-(phenylsulfanyl)thiophene 3af

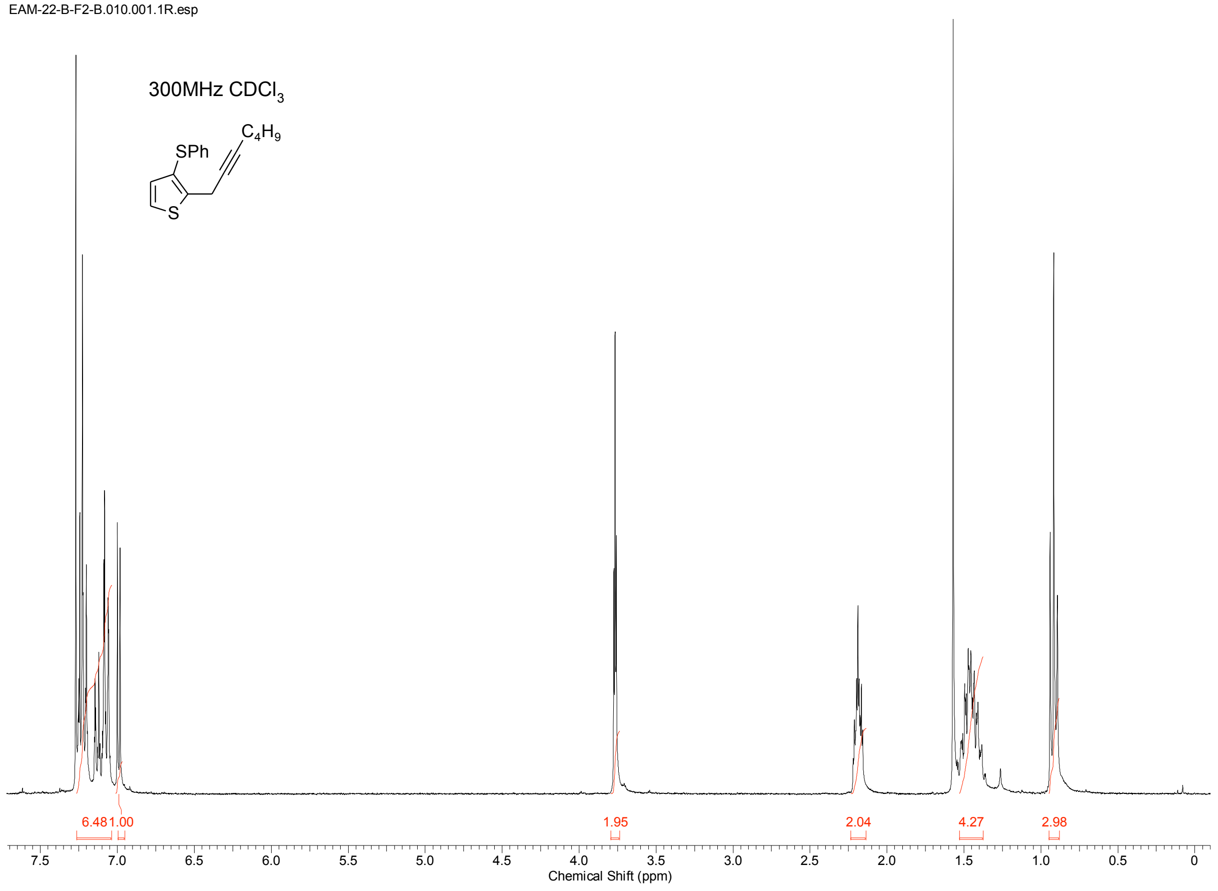


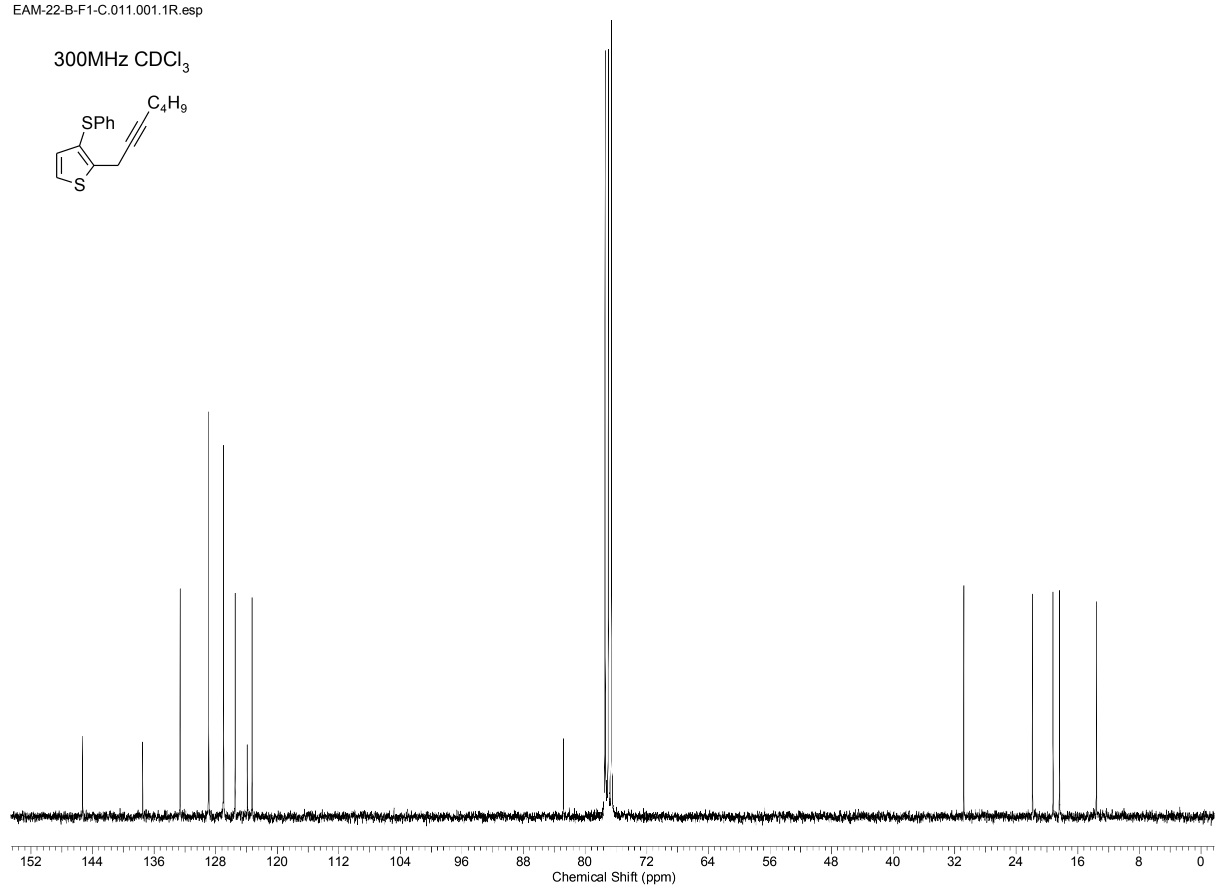

## 4-Bromo-2-(hept-2-yn-1-yl)-3-(phenylsulfanyl)thiophene 3ag

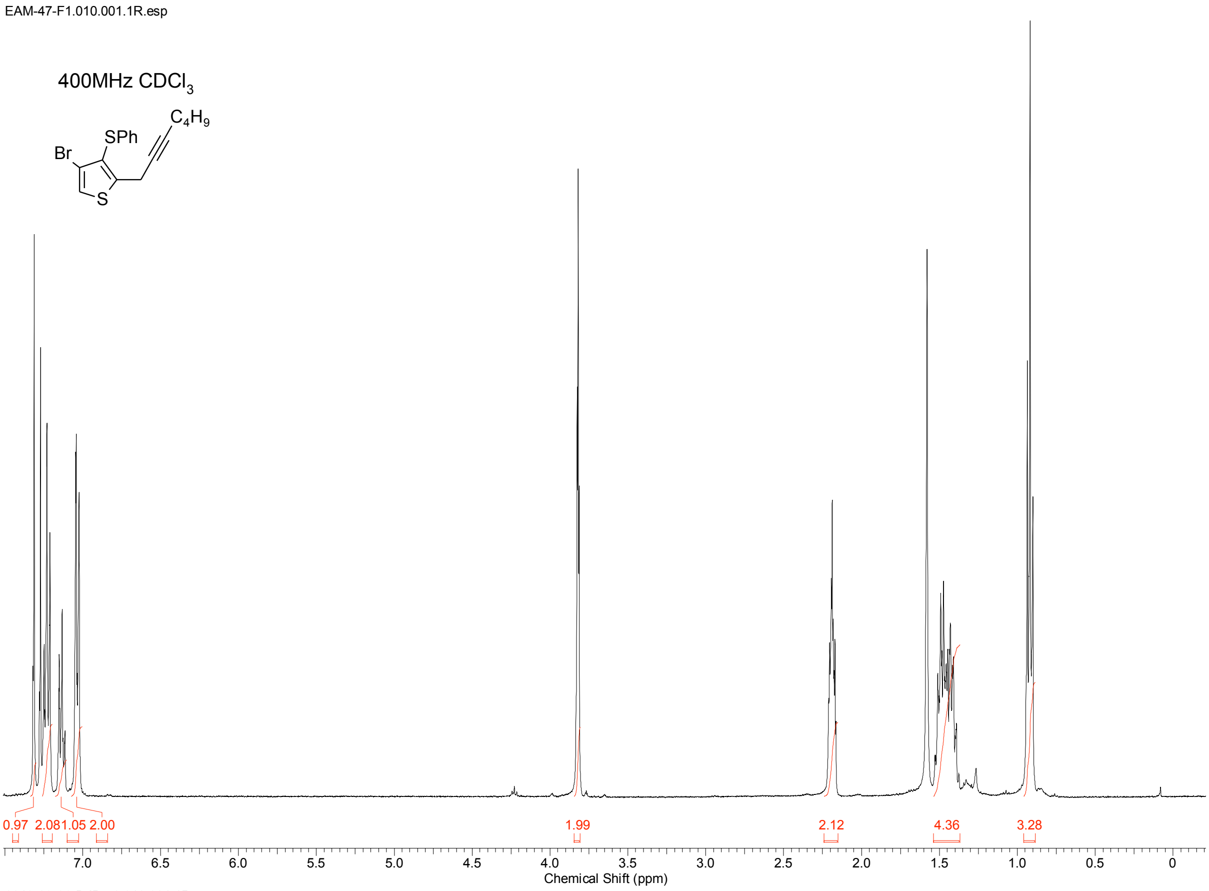


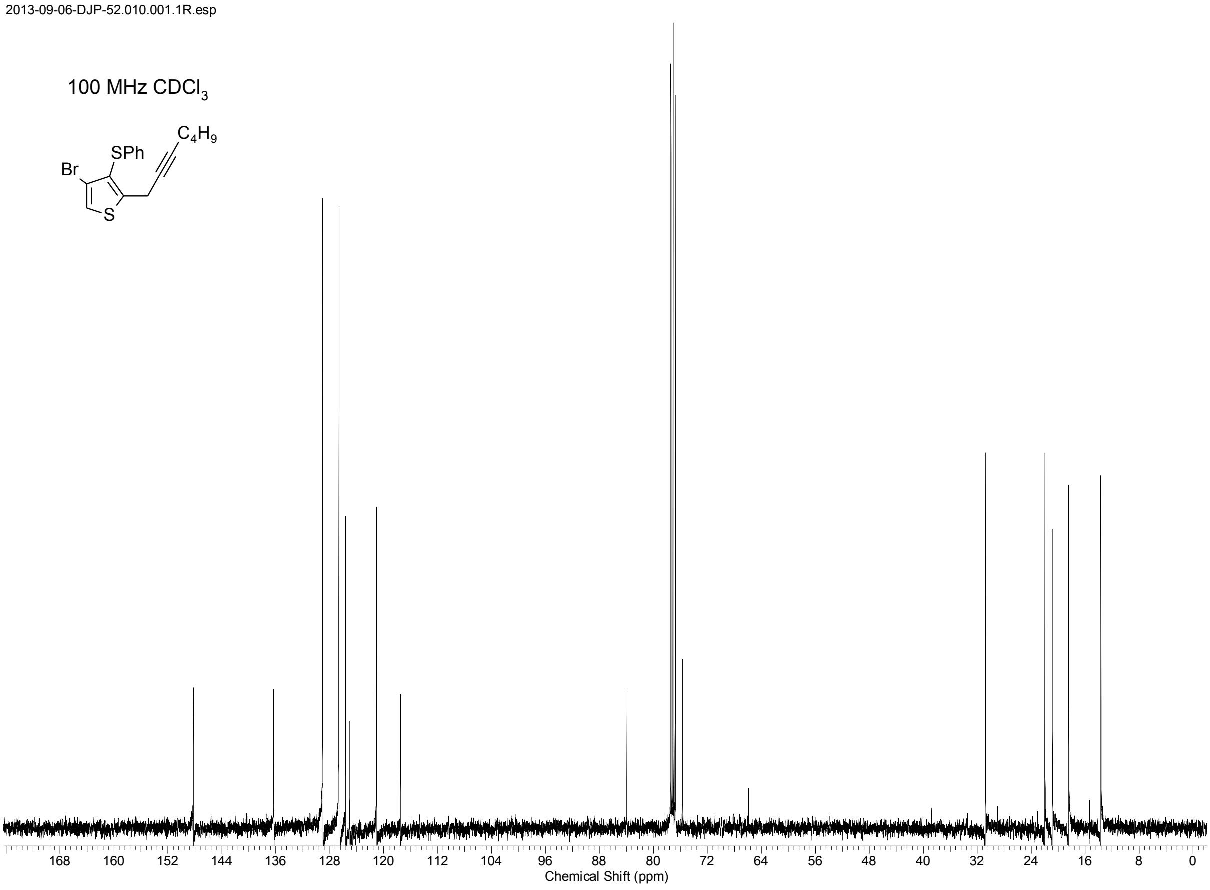

## 2-(Hept-2-yn-1-yl)-3,4-bis(phenylsulfanyl)thiophene 3ah

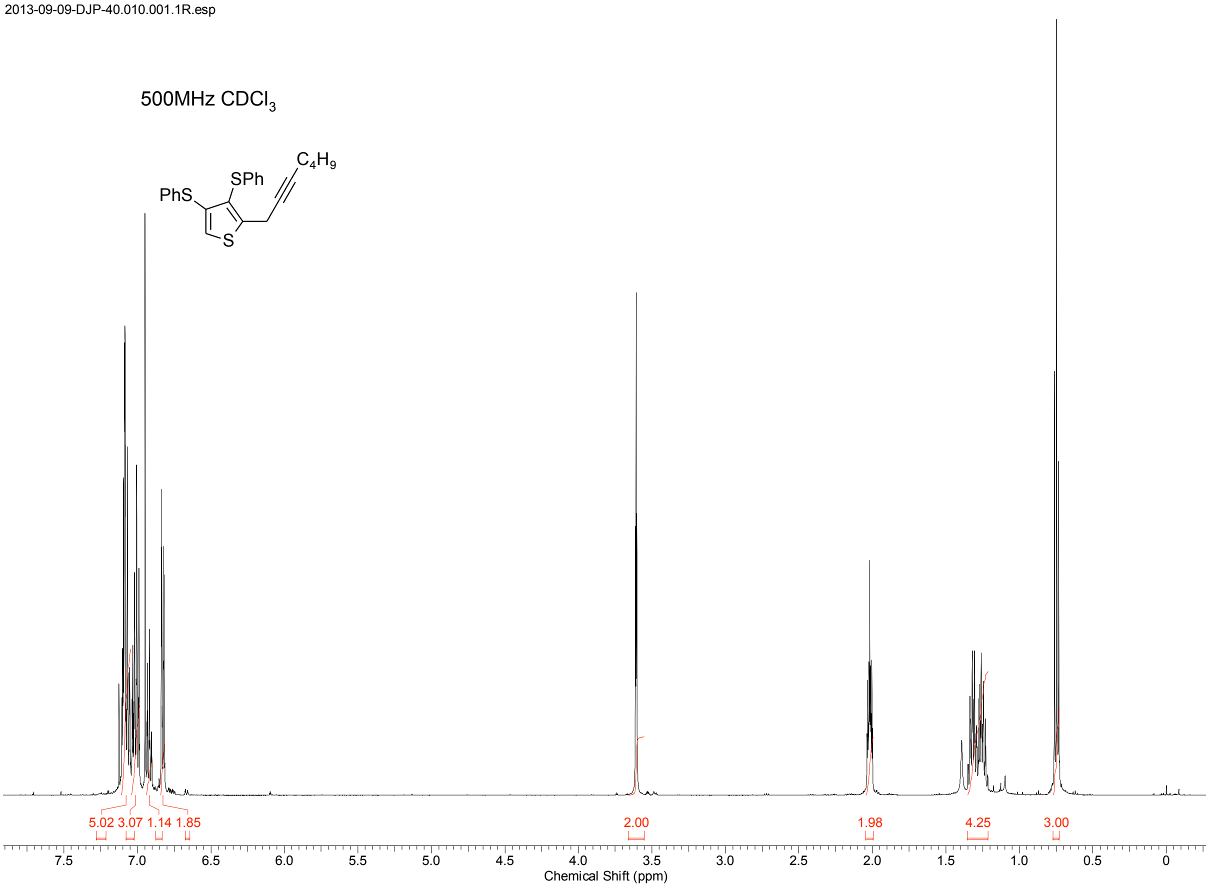


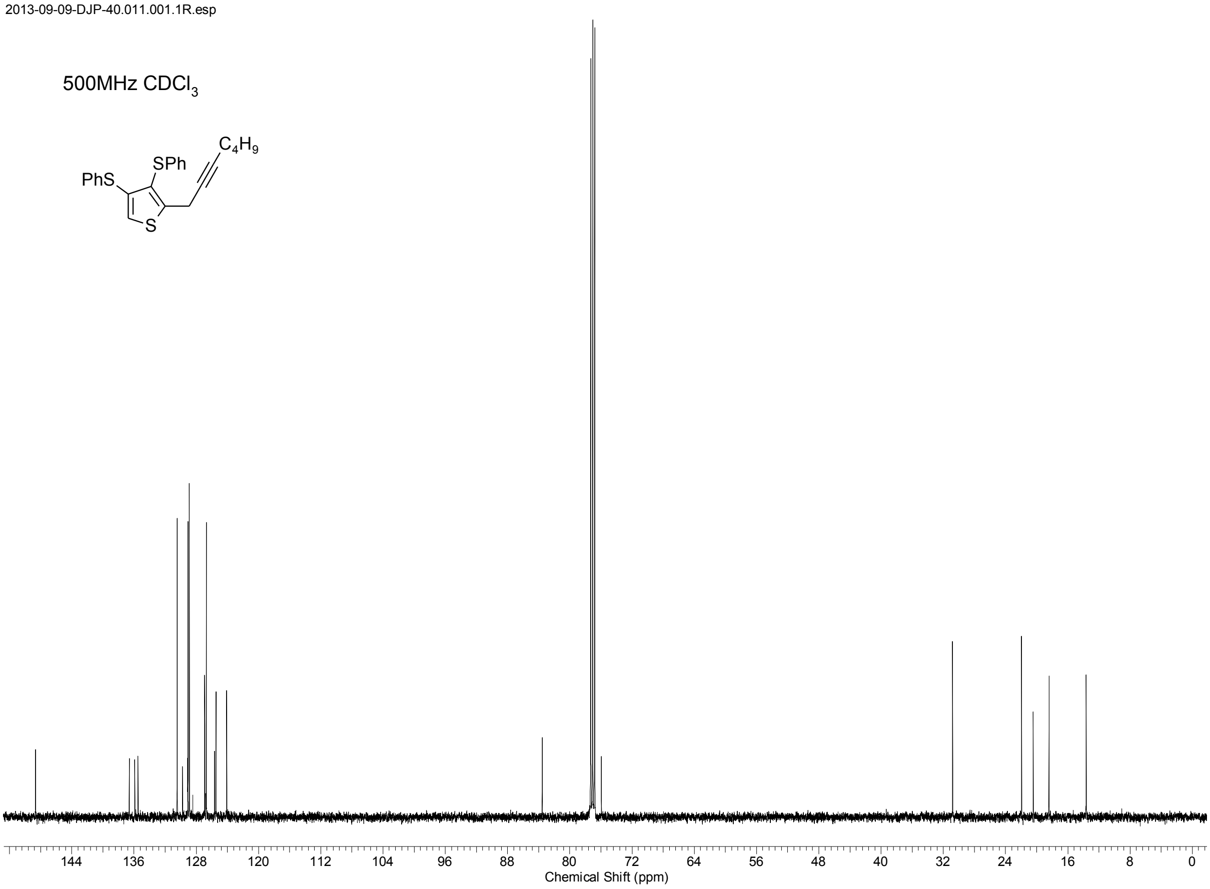

# 3-(Hept-2-yn-1-yl)-2-(phenylthio)thiophene 3ai

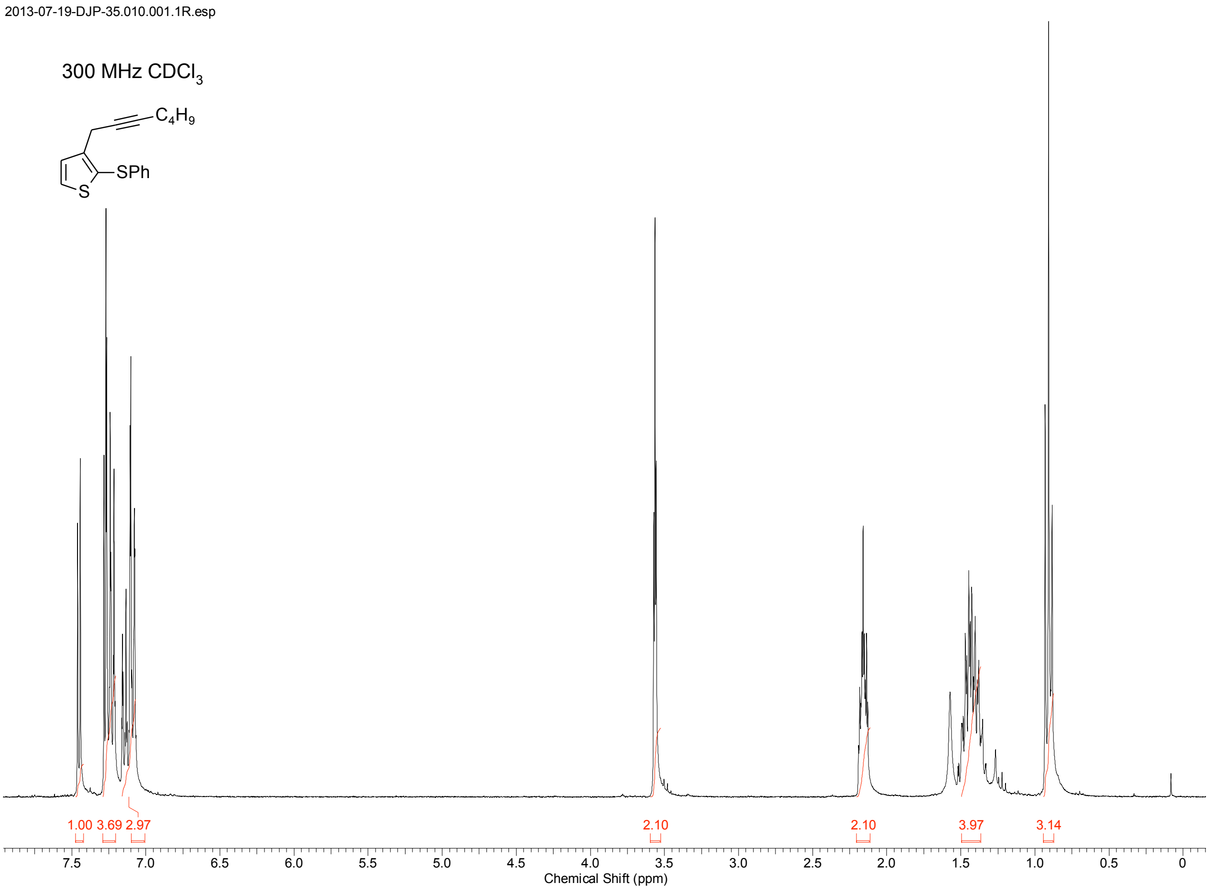


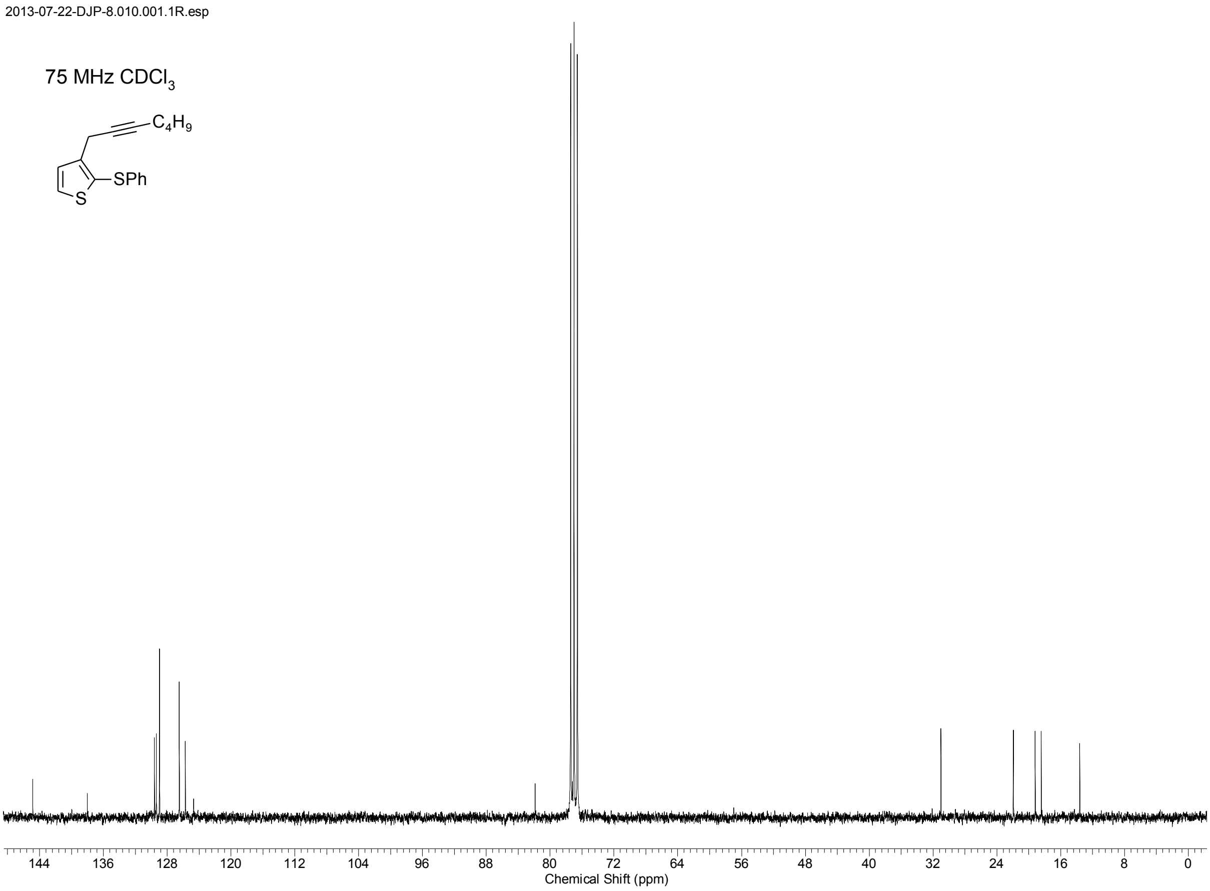

## 2-(Hept-2-yn-1-yl)-3-(phenylsulfanyl)furan 3aj

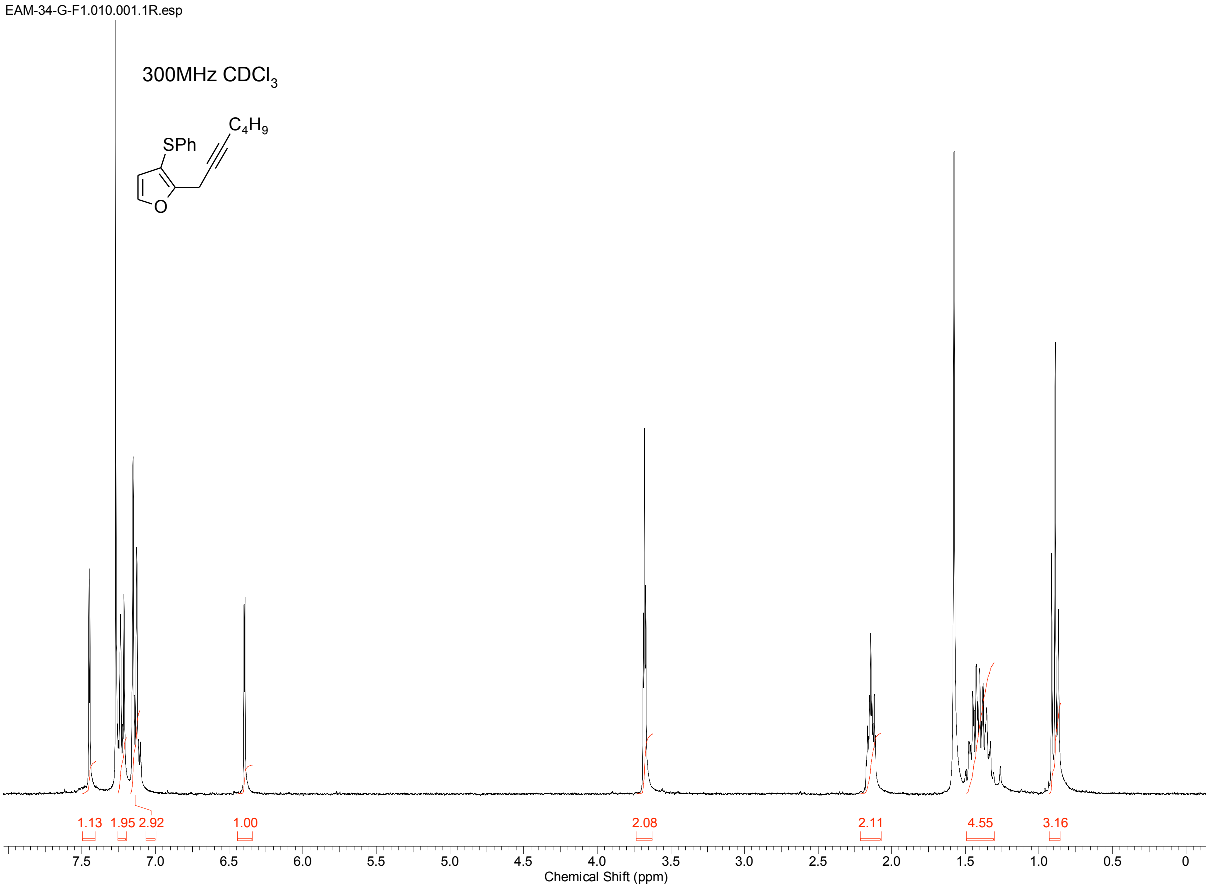


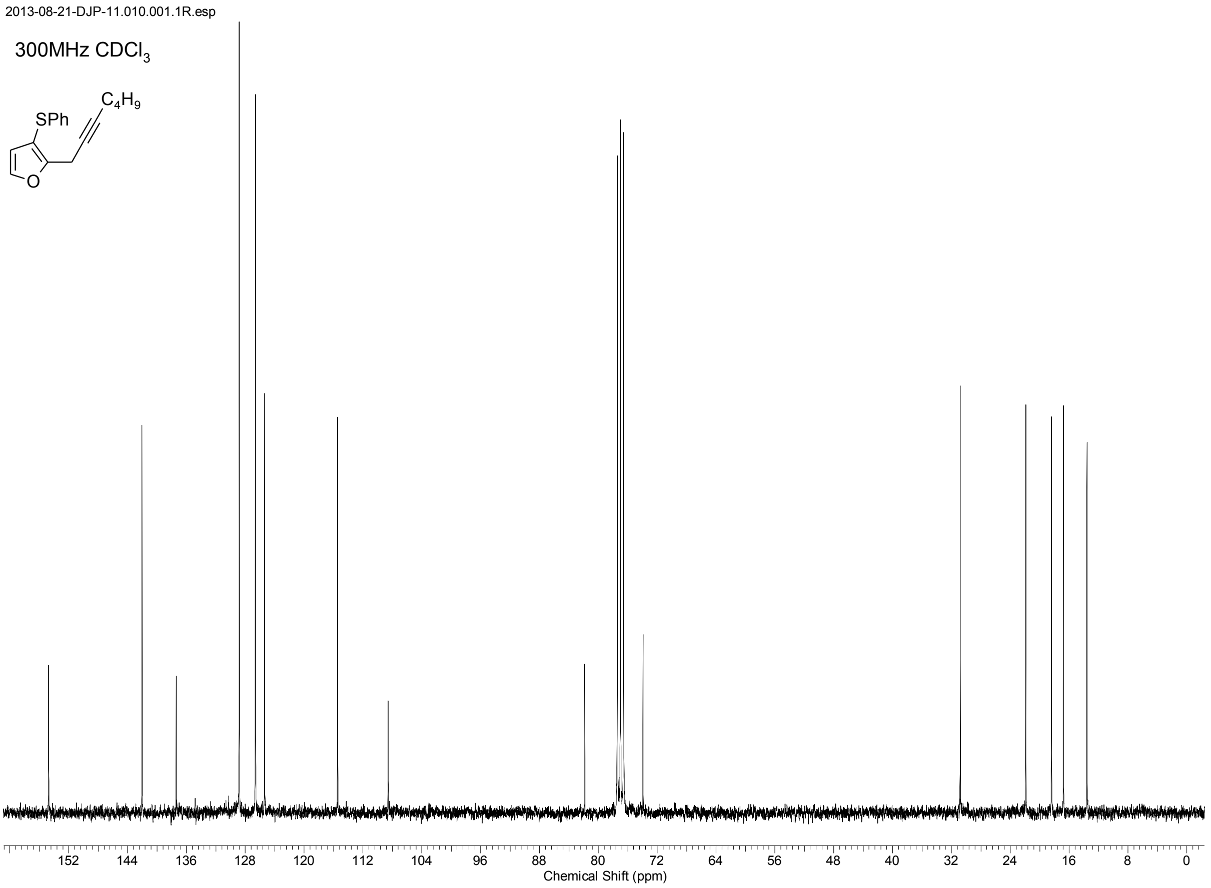

## 2,5-Di(hept-2-yn-1-yl)-3,4-*bis*(phenylthio)thiophene 3ak

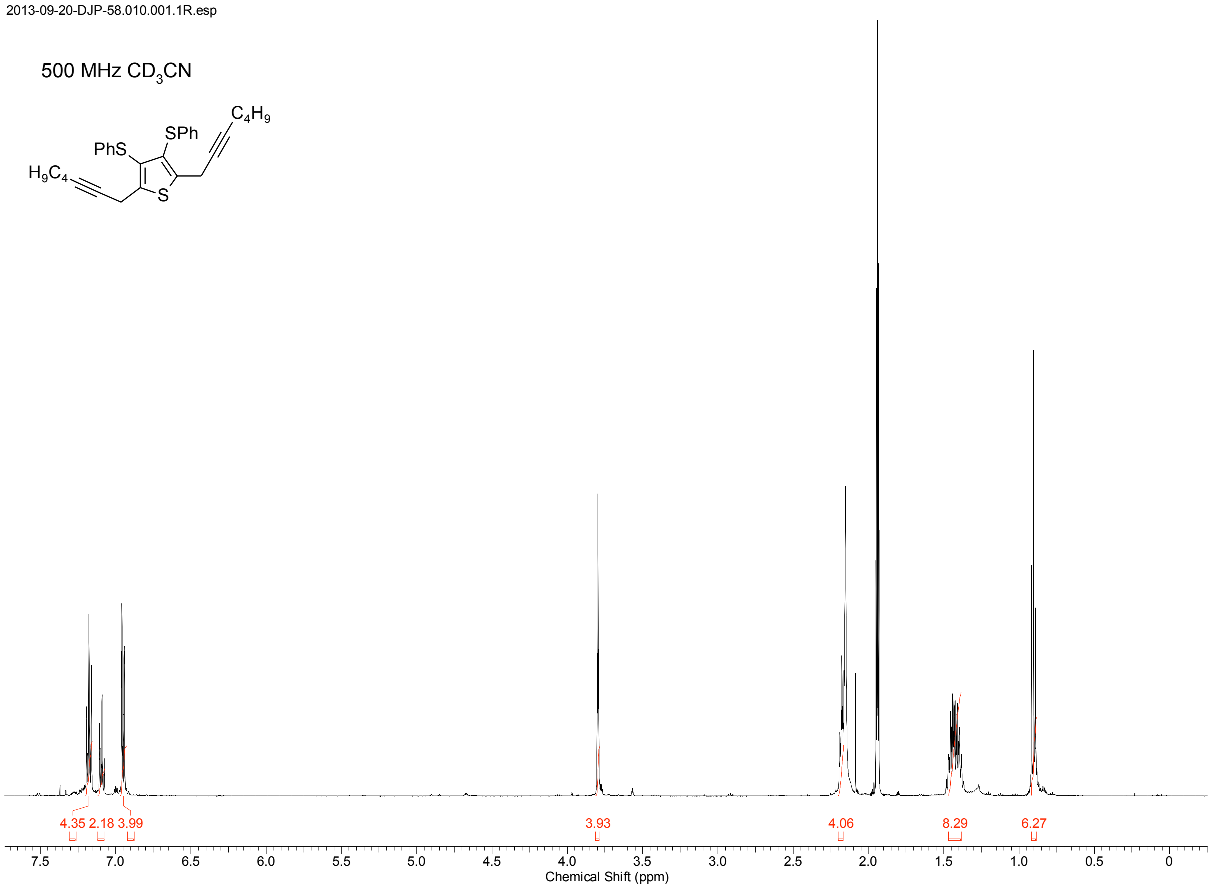


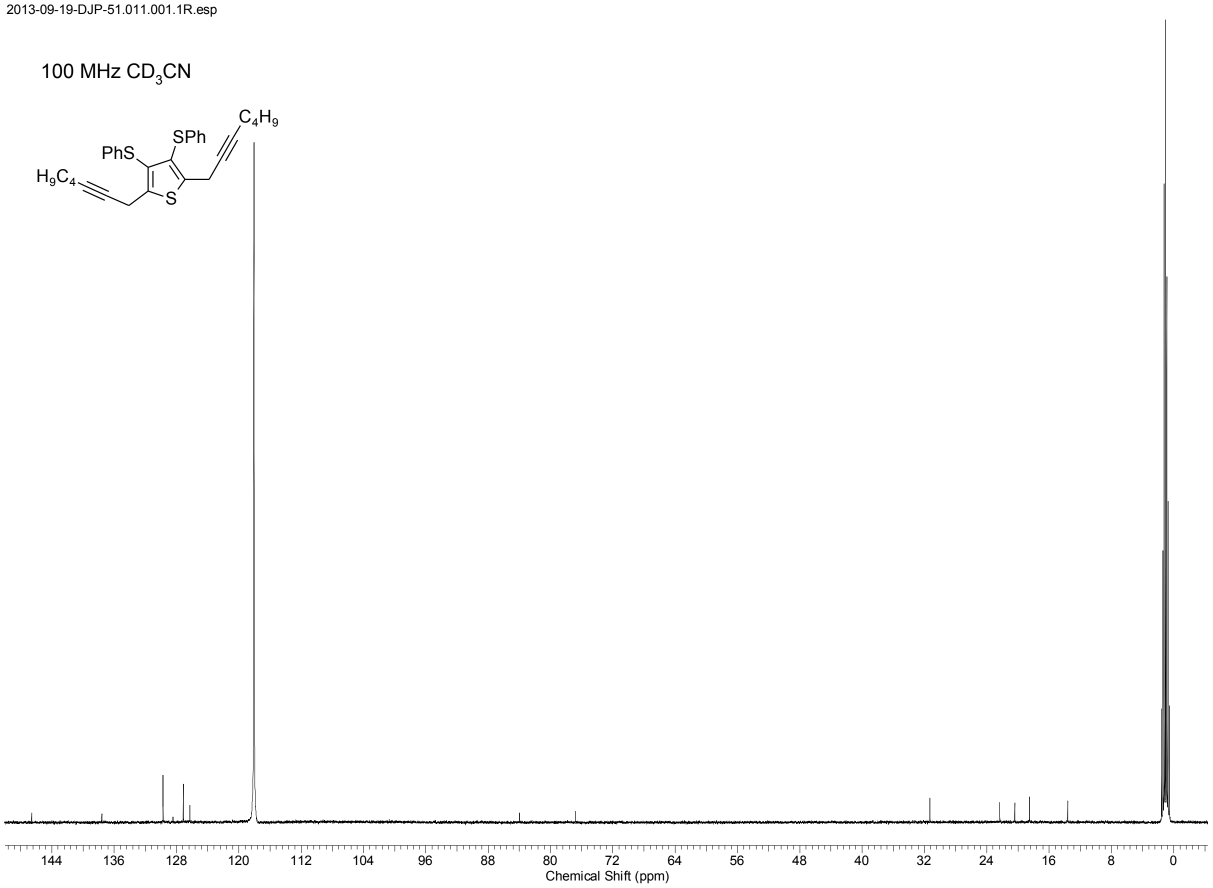

## Trimethyl(3-(3-(methylsulfanyl)thiophen-2-yl)prop-1-yn-1-yl)silane 3aq

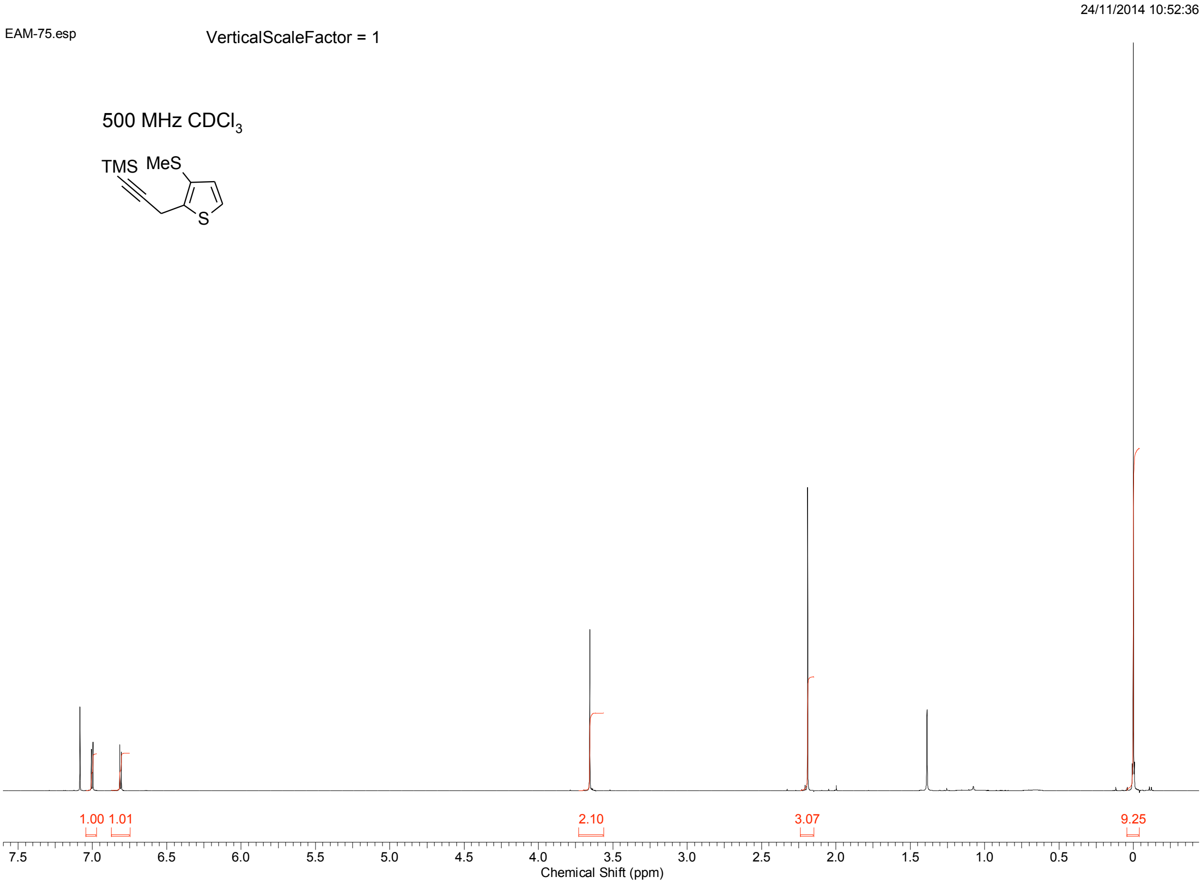


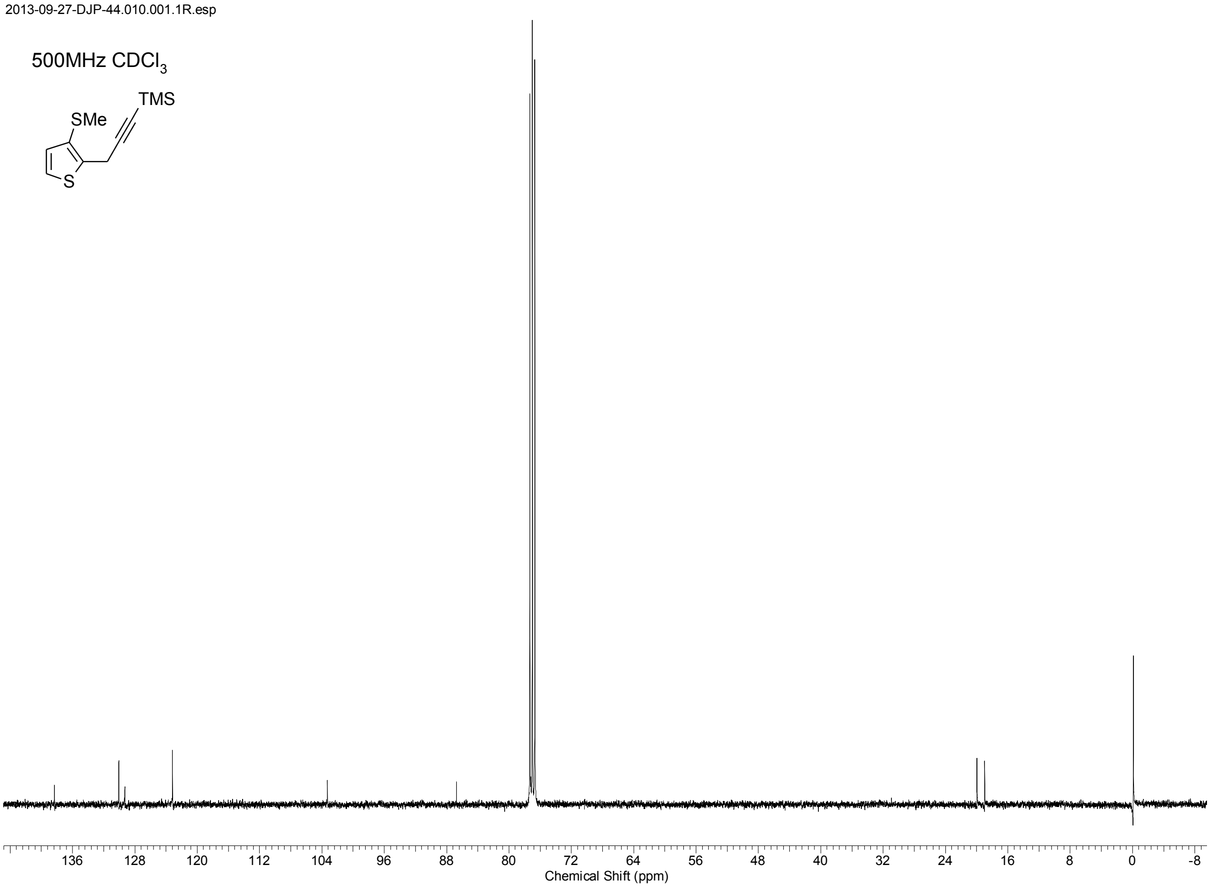

# 2-(3-Cyclohexylprop-2-yn-1-yl)-3-(methylsulfanyl)thiophene 3ar

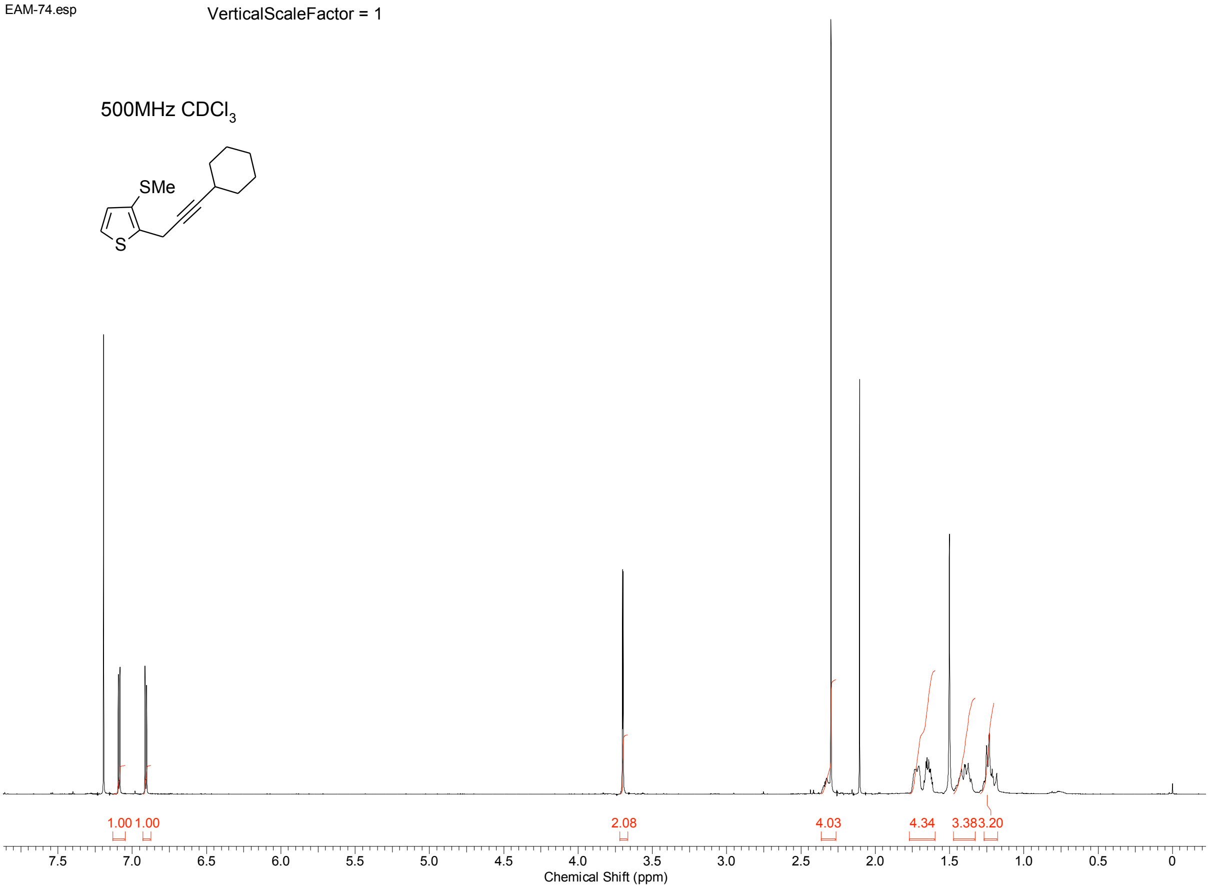


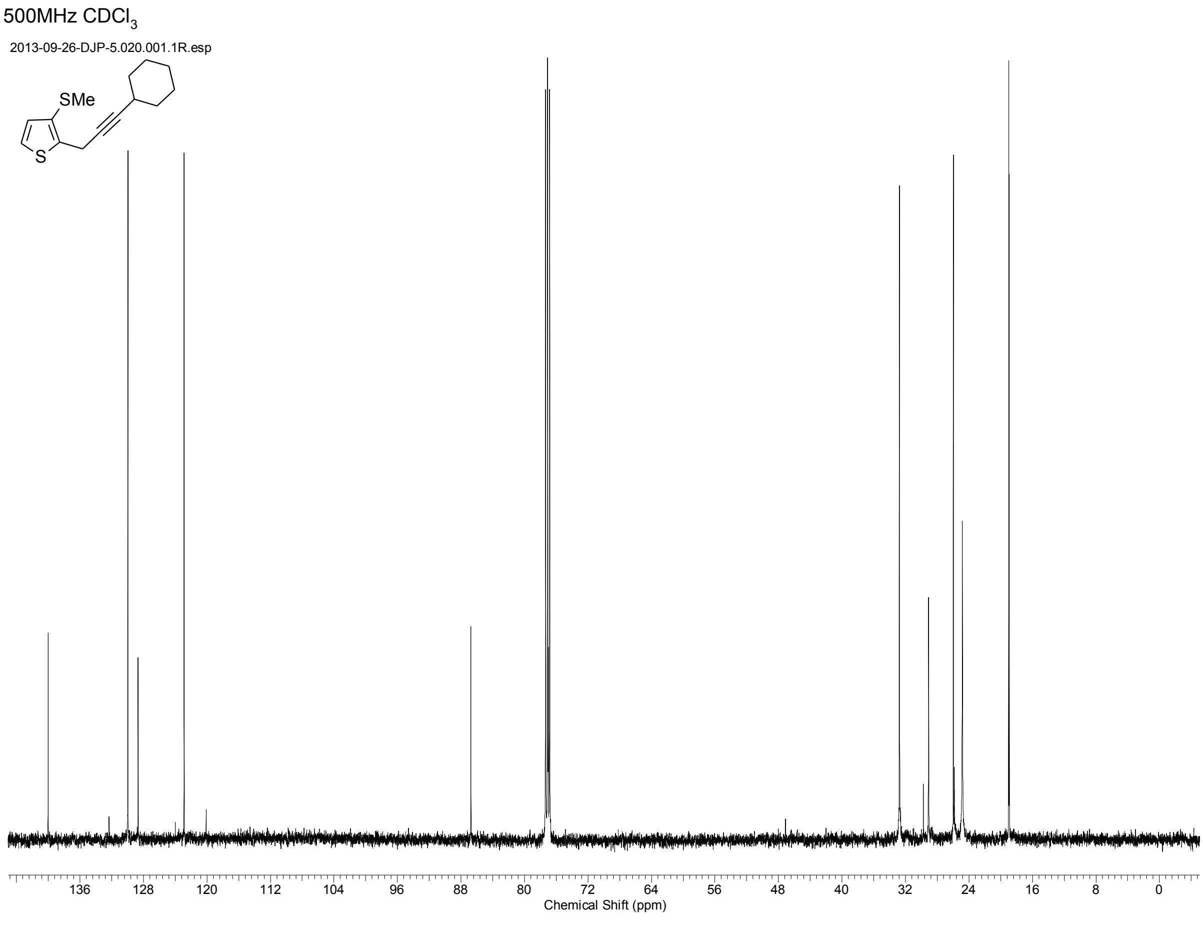

## Trimethyl(3-(3-(methylsulfanyl)thiophen-2-yl)hex-1-yn-1-yl)silane 3at

2013-09-24-DJP-44.010.001.1R.esp

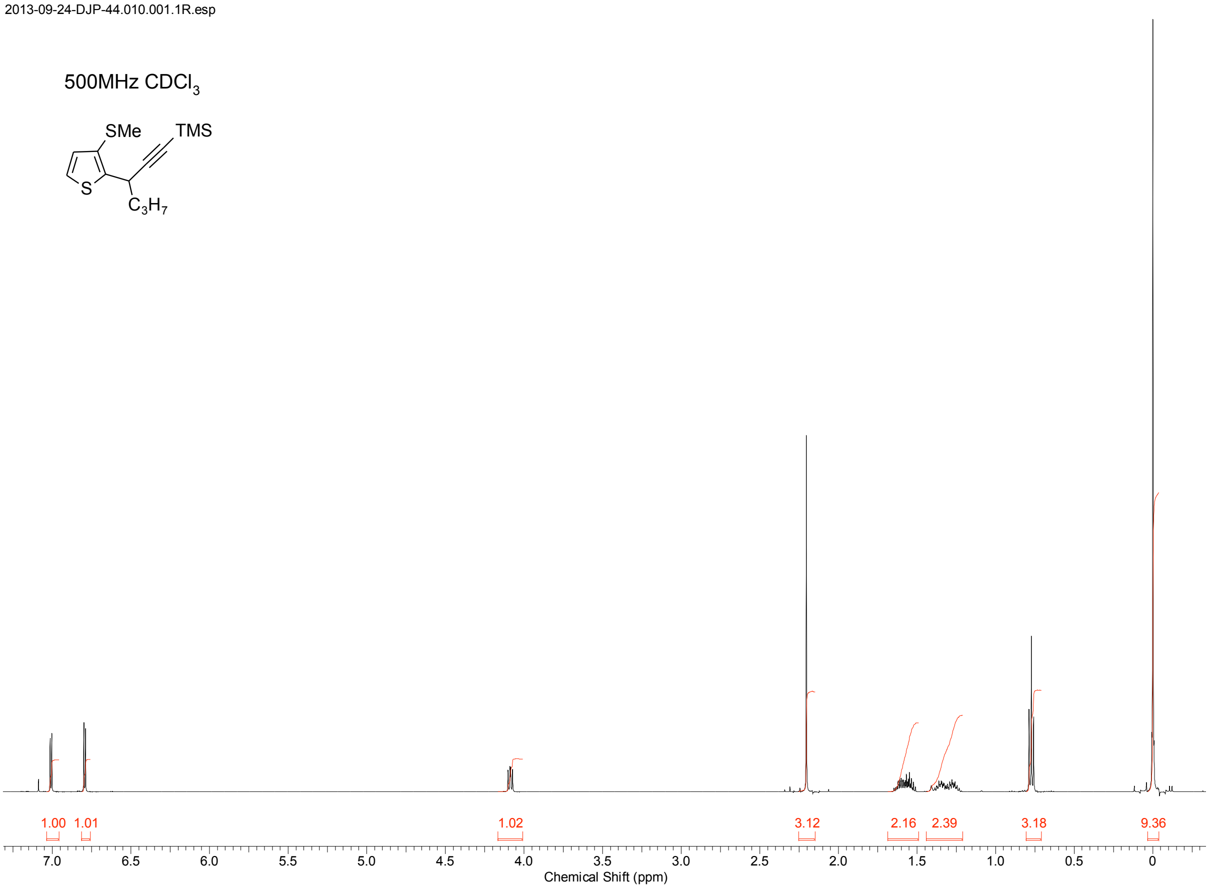


2013-09-24-DJP-44.011.001.1R.esp

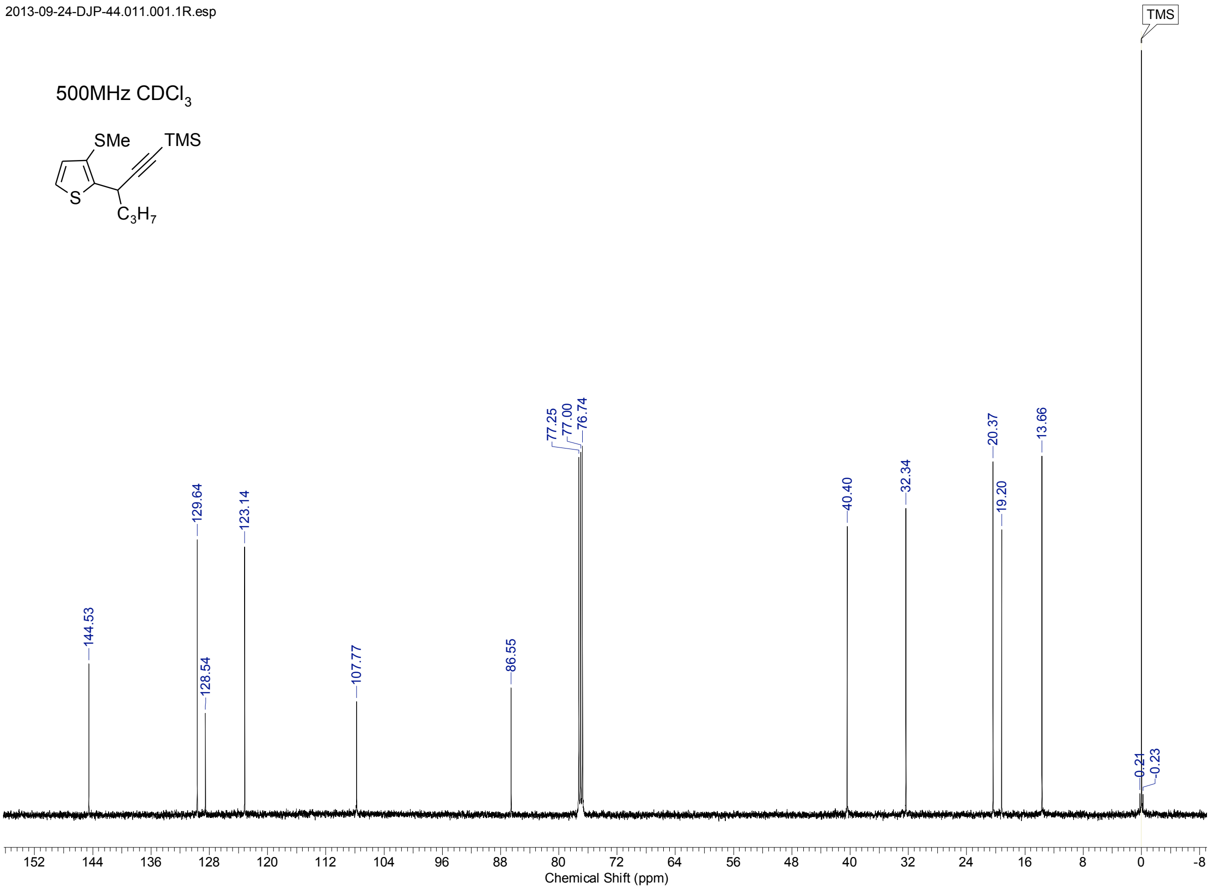

## Trimethyl(3(2-(methylsulfanyl)phenyl)hex-1-yn-1yl)silane 3au

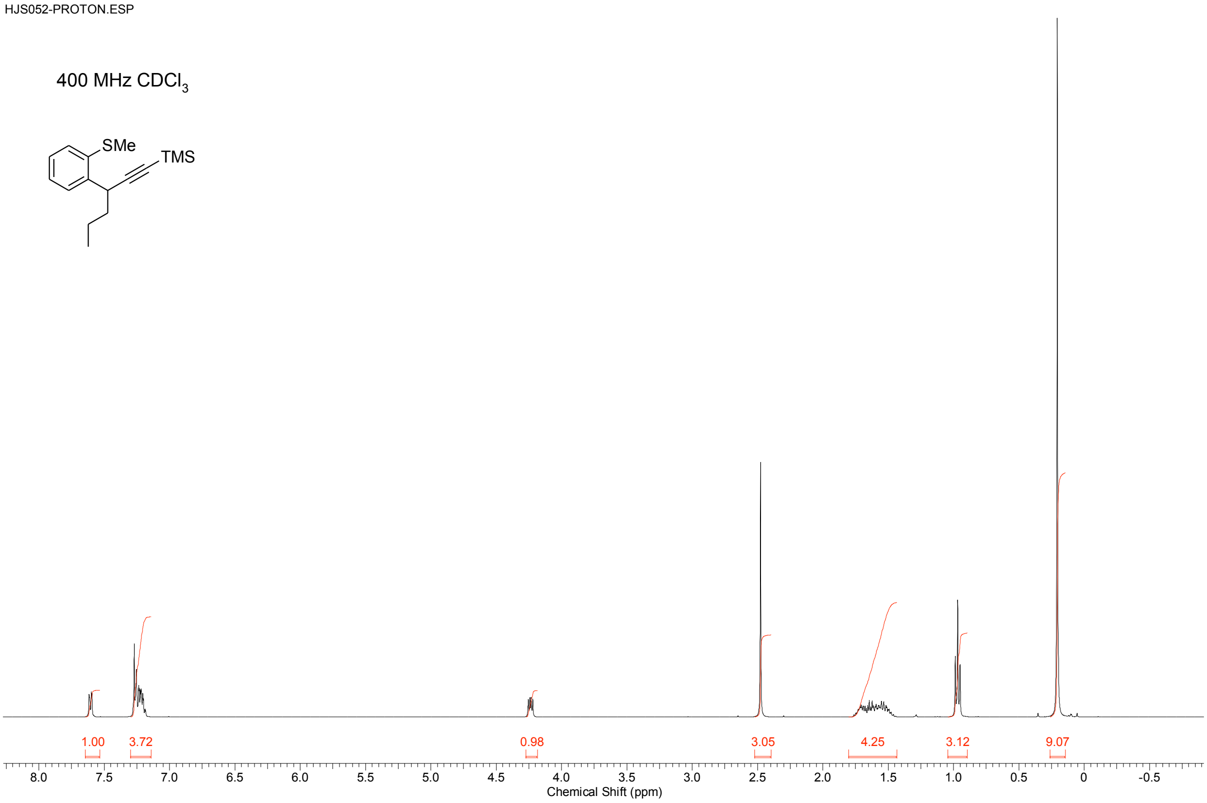


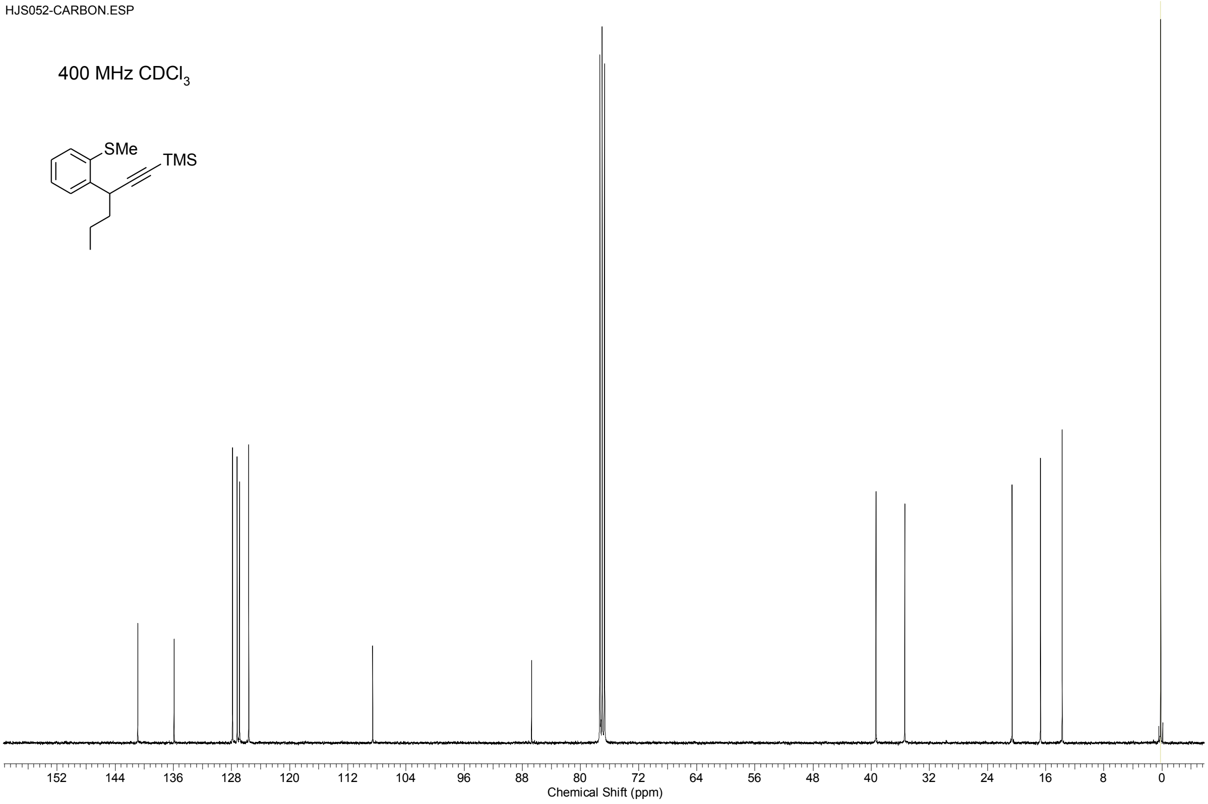

# Trimethyl(3-(2-methylsulfanyl)pent-1-yn-yl)silane 3av

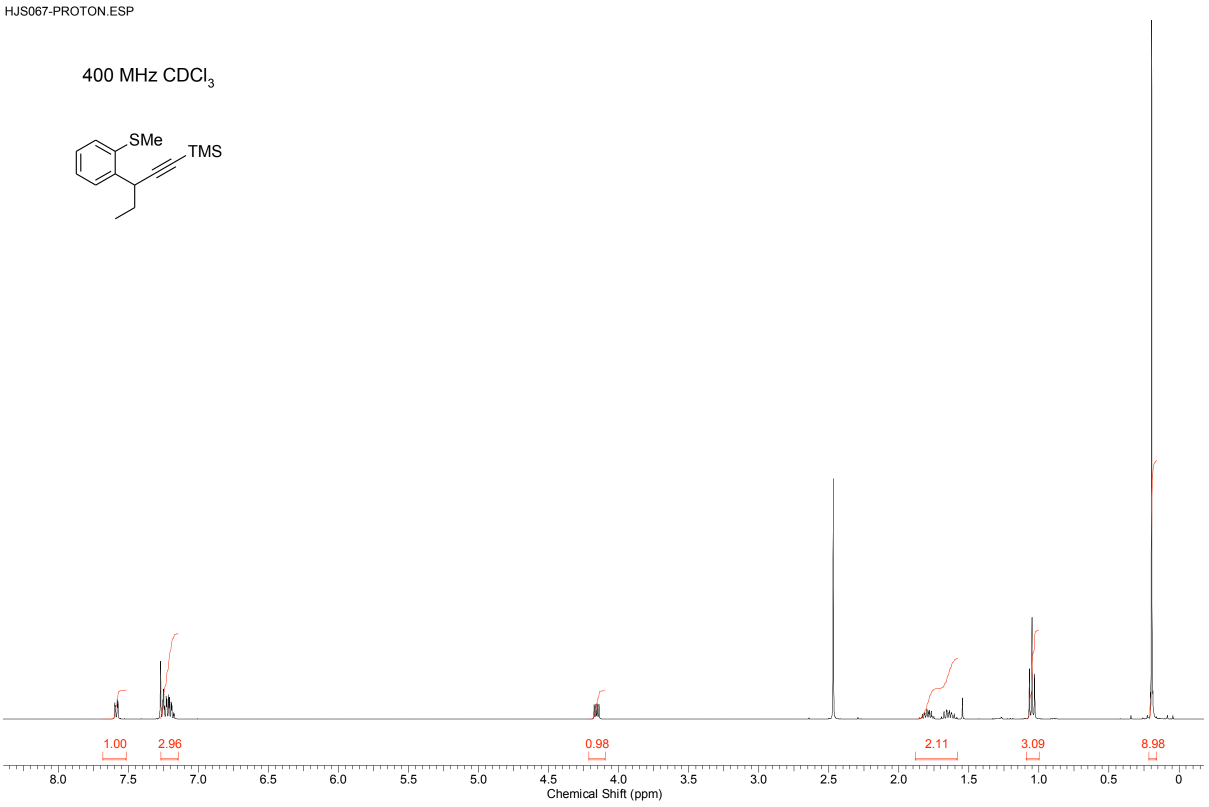


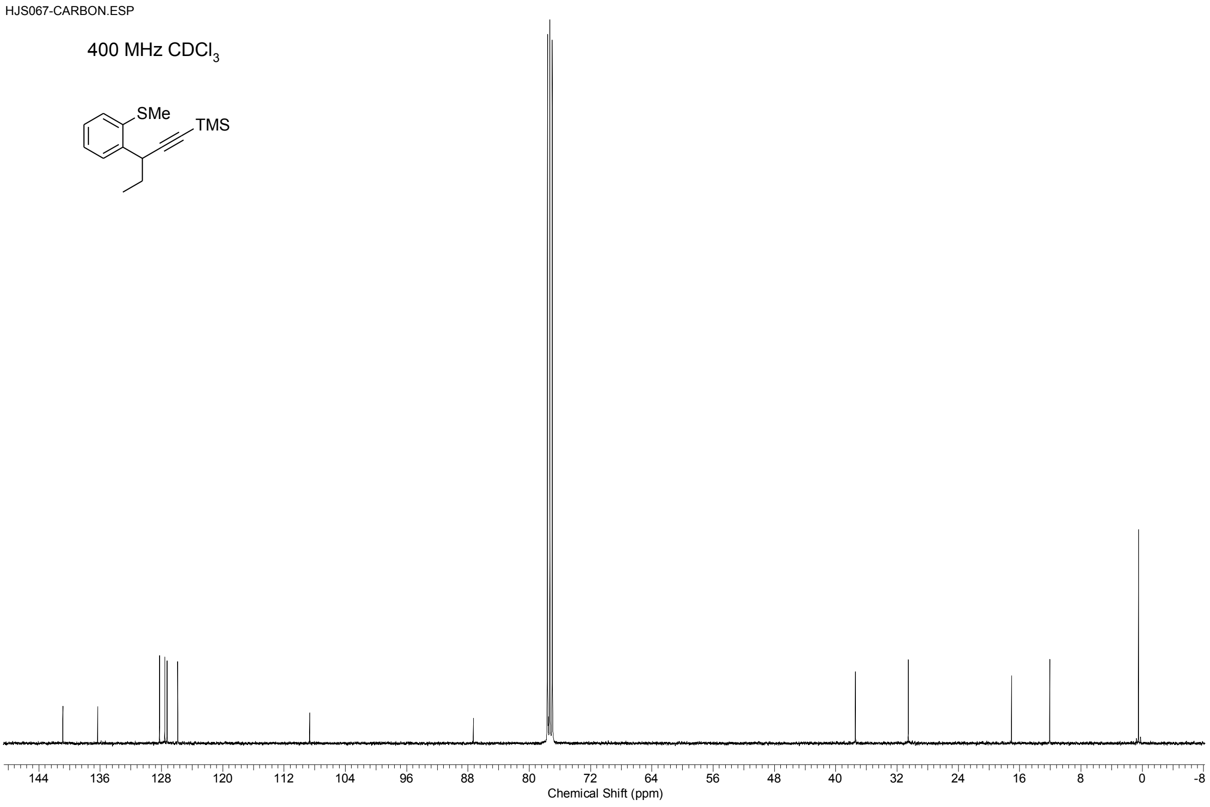

# Trimethyl(4-methyl-3-(2-(methylsulfanyl)phenyl)pent-1-yn-1-yl)silane 3aw

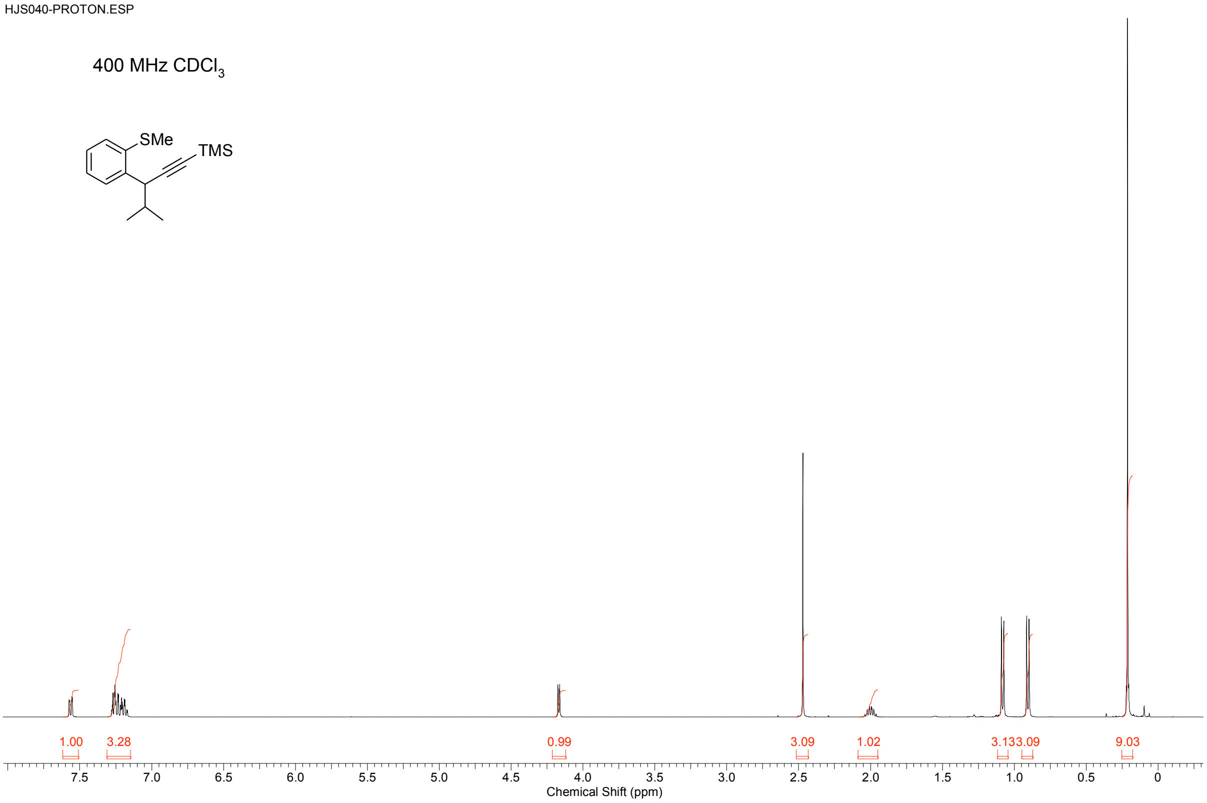


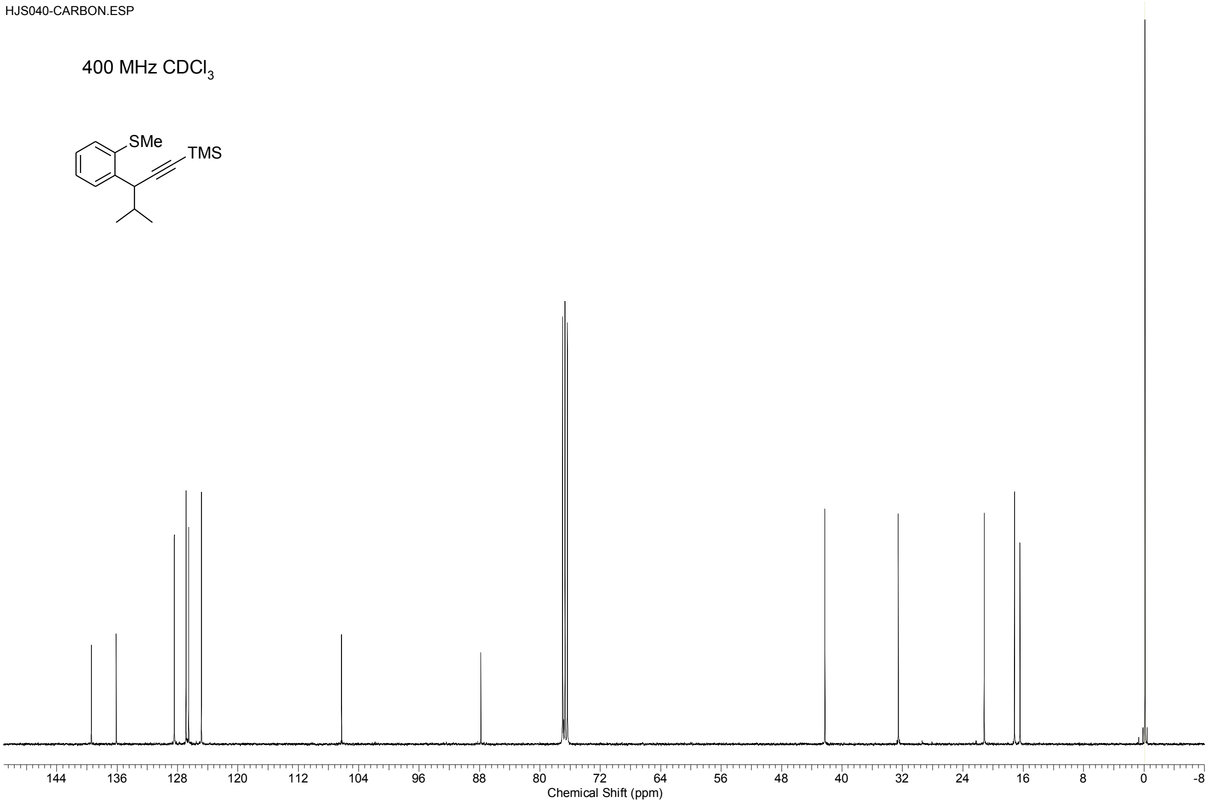

## (3-Cyclopentyl-3-(2-(methylsulfanyl)phenyl)prop-1-yn-1-yl)trimethylsilane 3ax

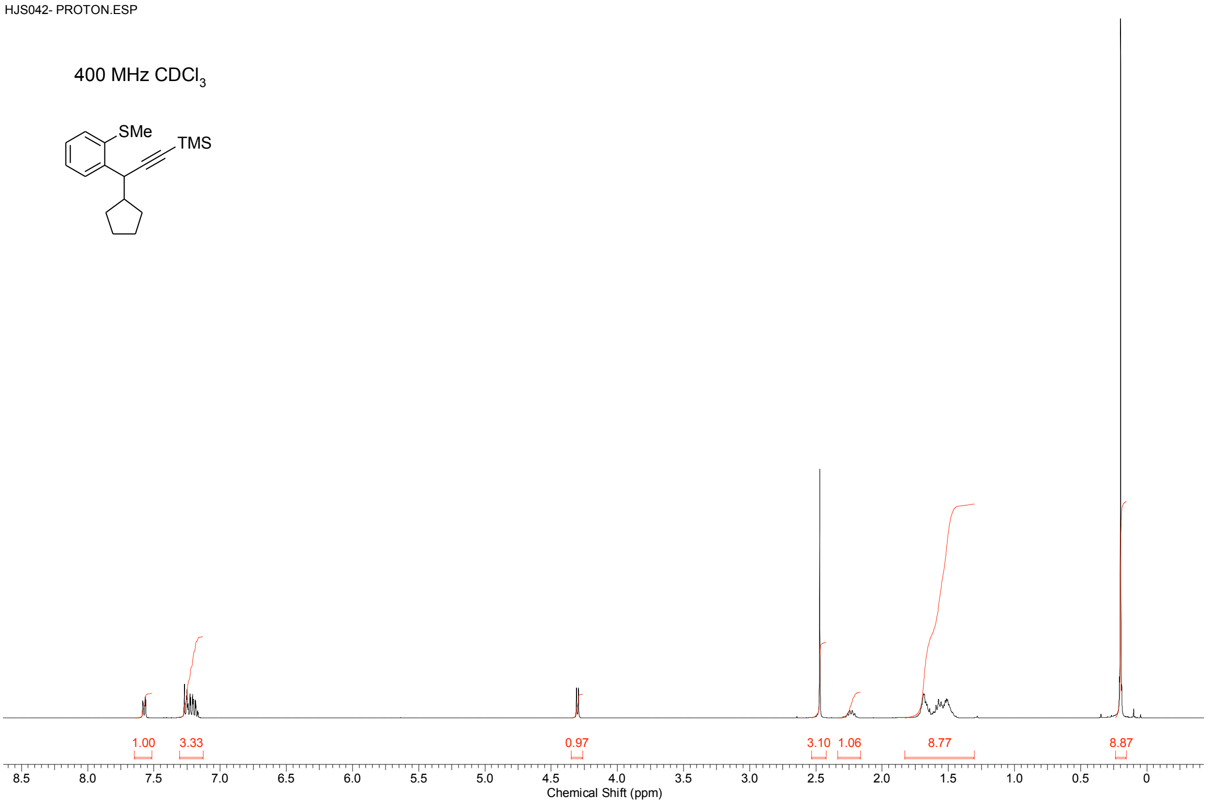


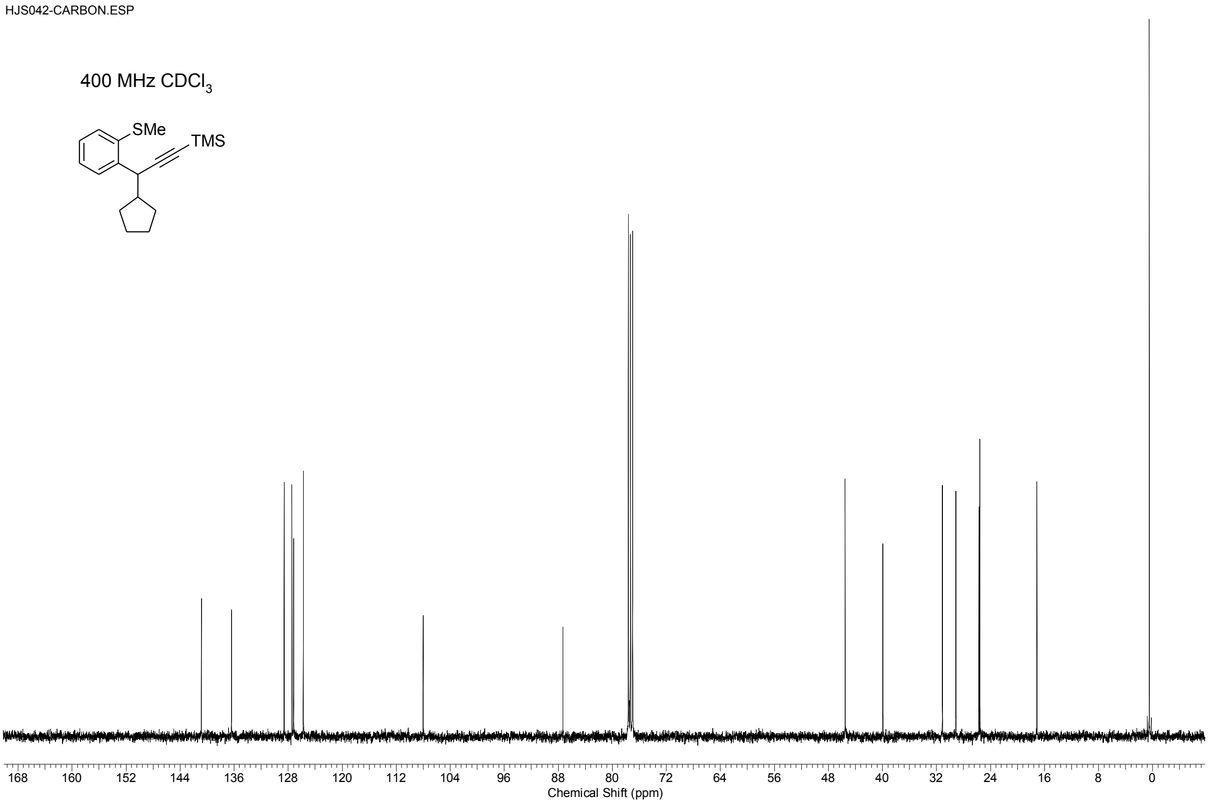

## (3-Cyclohexyl-3-(2-methylsulfanyl)phenyl)prop-1-yn-1-yl)trimethylsilane 3ay

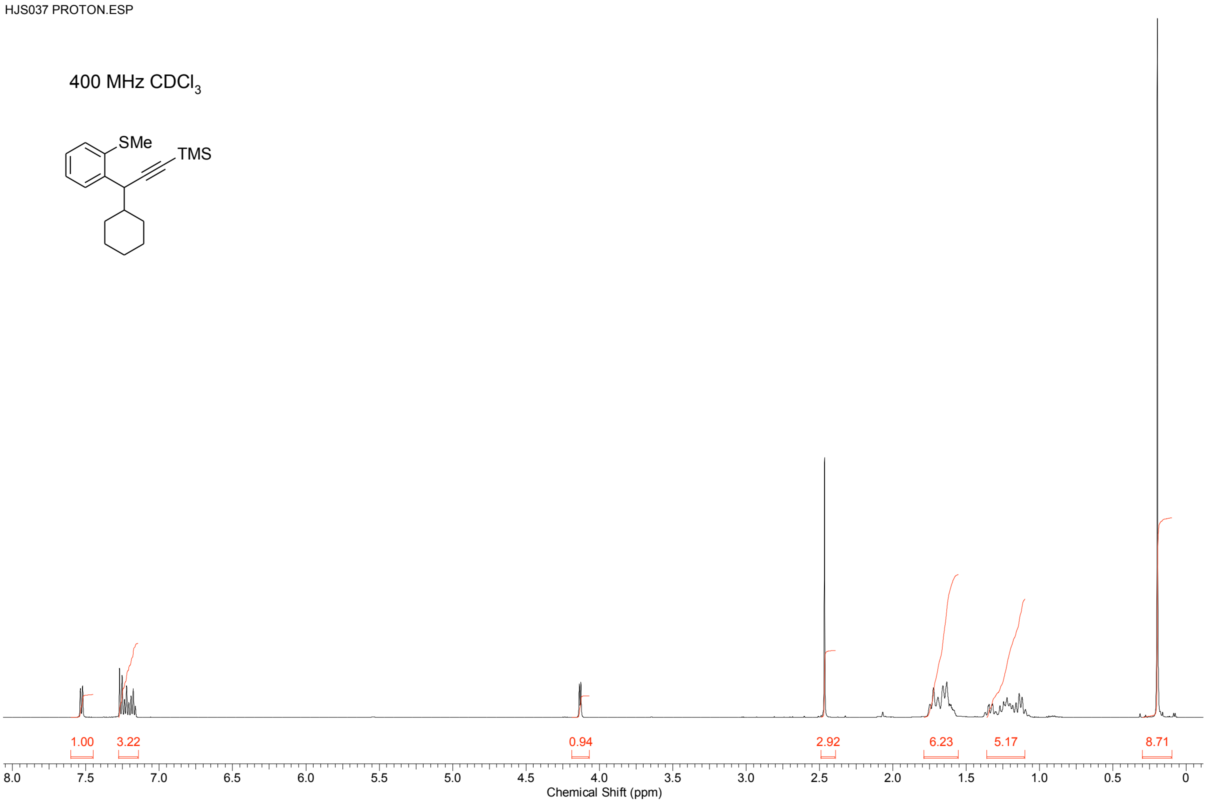


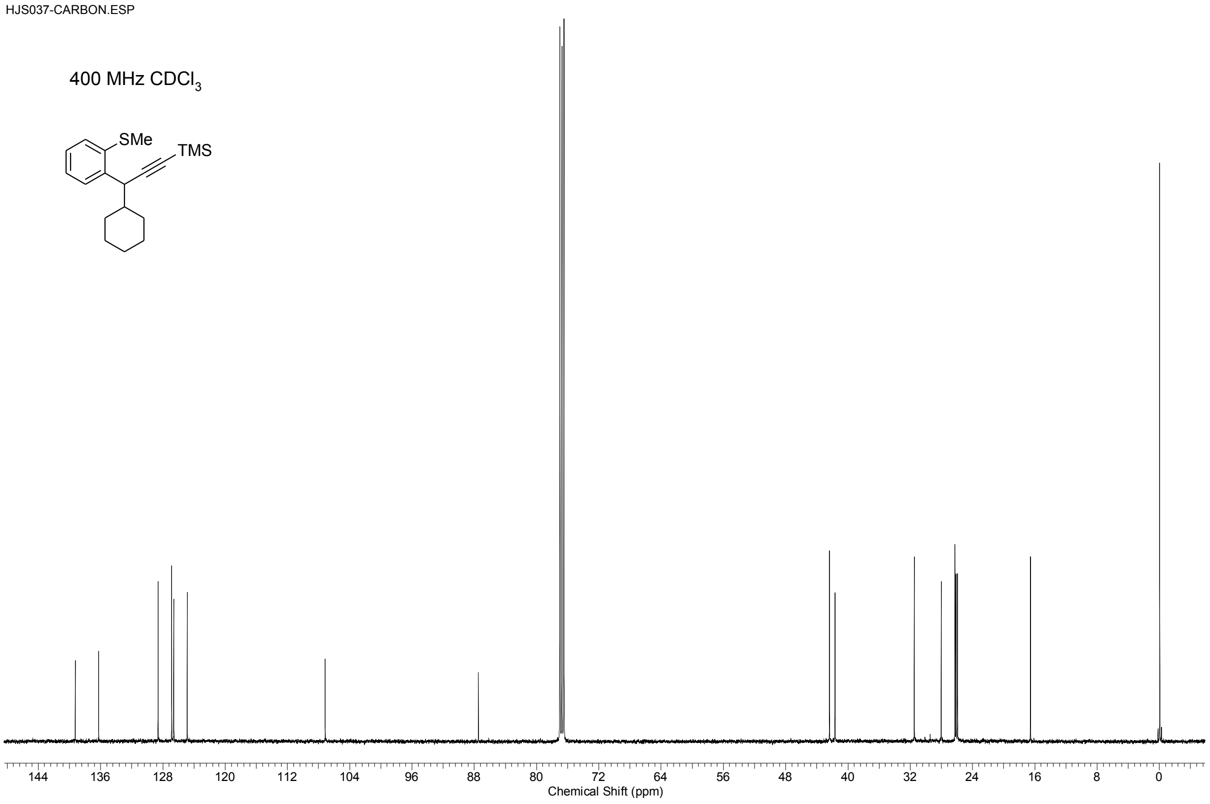

# (2-(Hex-3-yn-2-yl)phenyl)(methyl)sulfide 3az

HJS062-PROTON.ESP

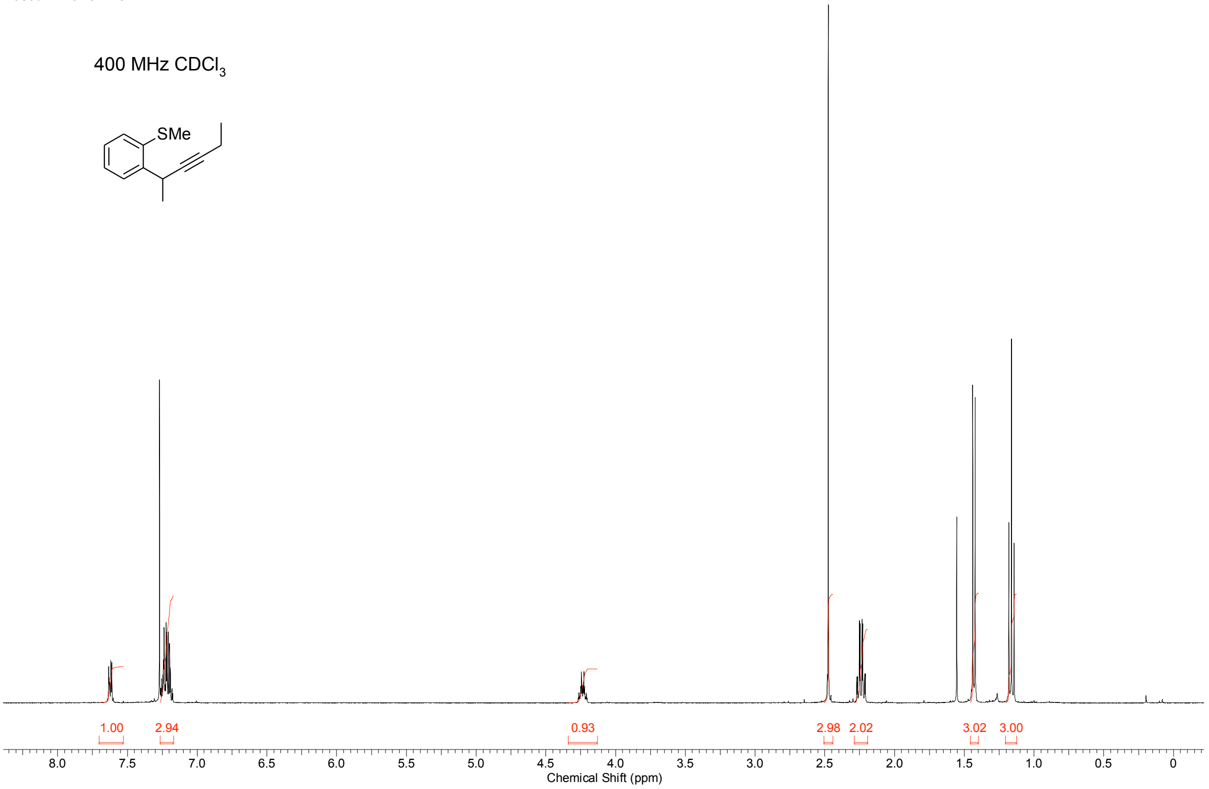


HJS062-CARBON.ESP

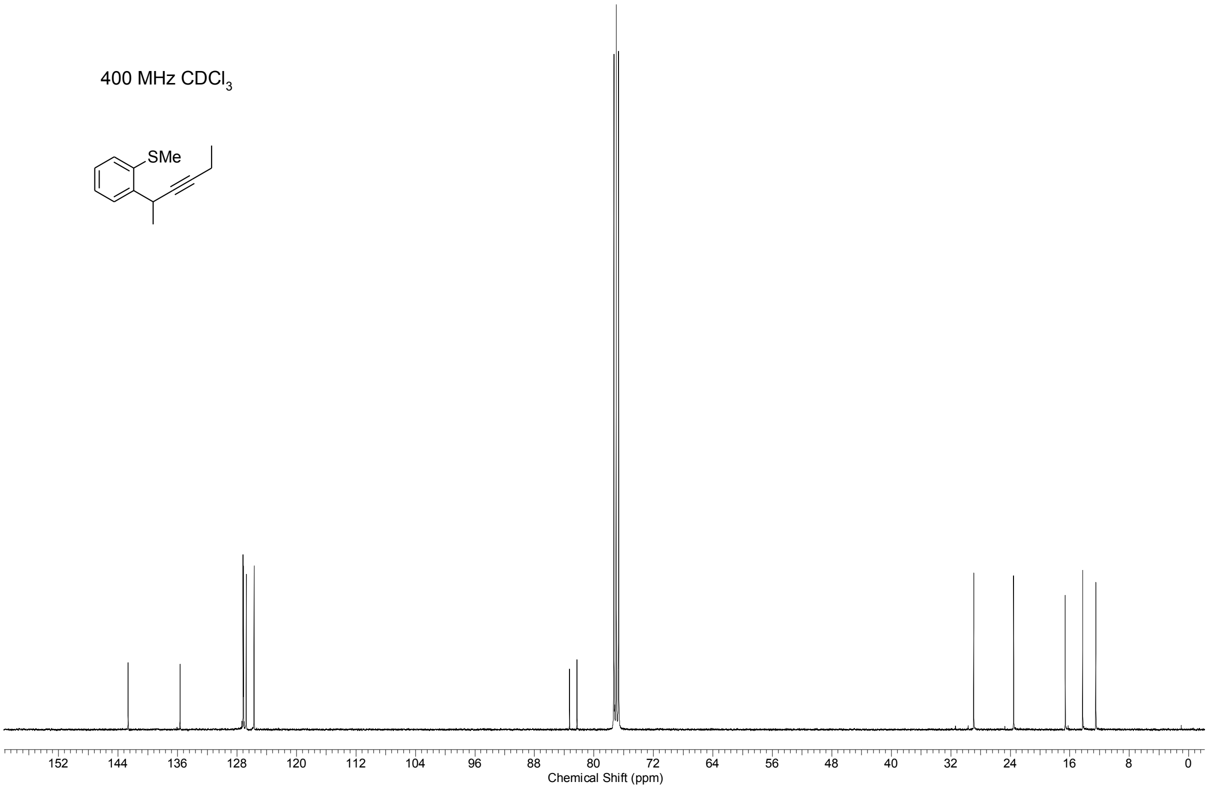

# Methyl(2-(oct-4-yn-3-yl)phenyl)sulfide 3ba

HJS065-PROTON.ESP

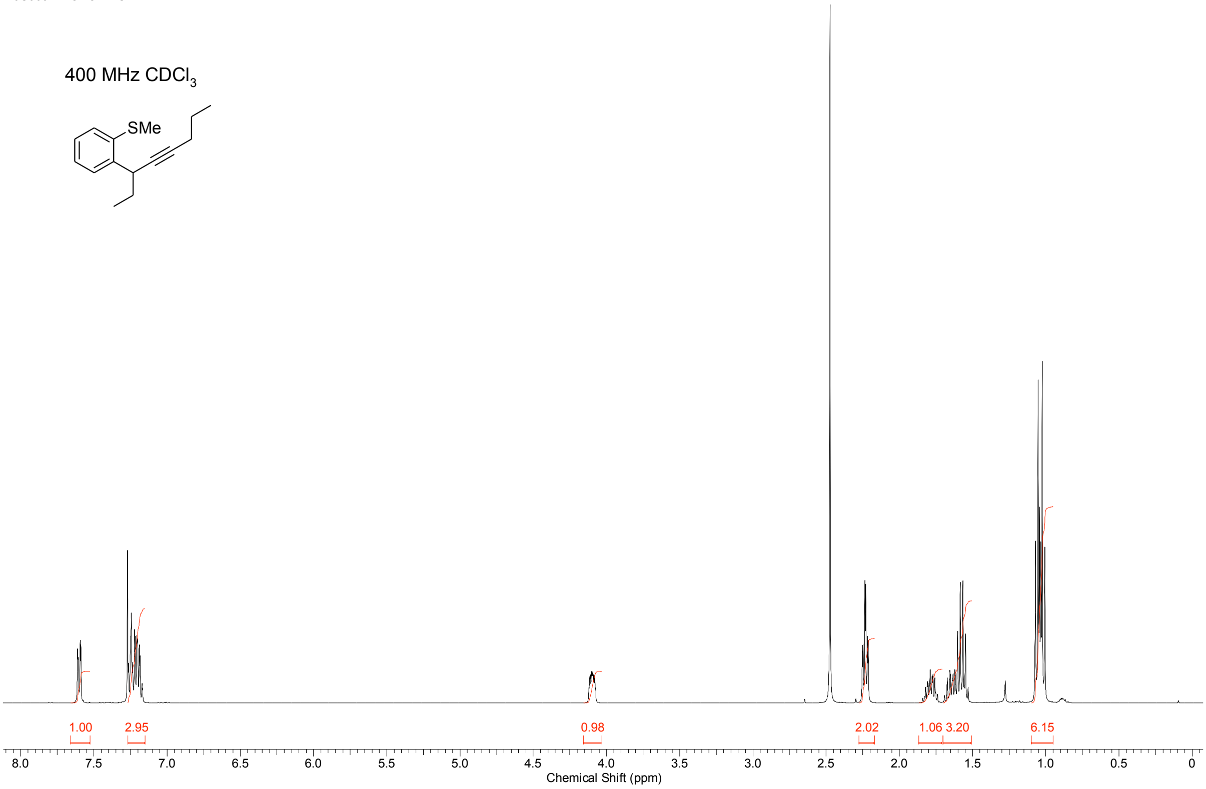


HJS065-CARBON.ESP

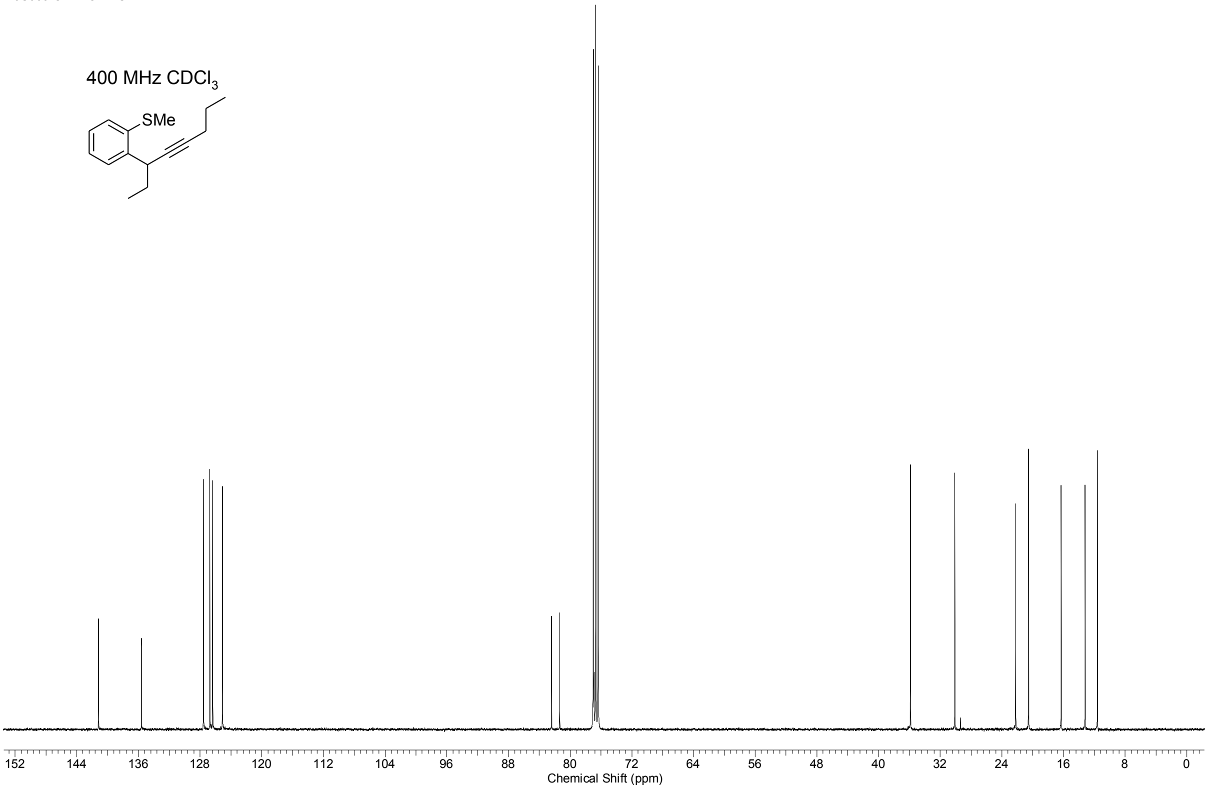

## 2-(Hept-2-yn-1-yl)-4-methoxy-1-(methylsulfinyl)benzene 1al

AC-063-1.010.001.1R.esp

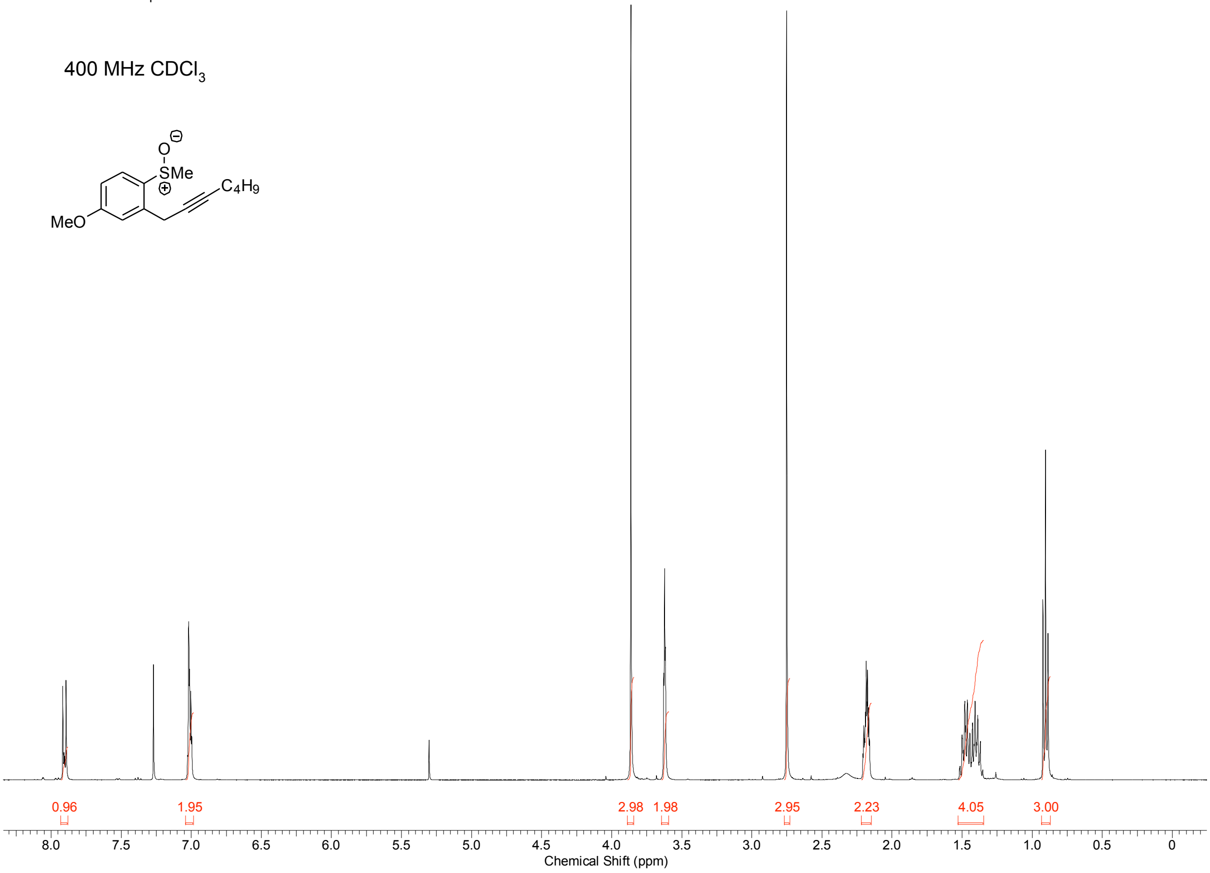


AC-063-1.011.001.1R.esp

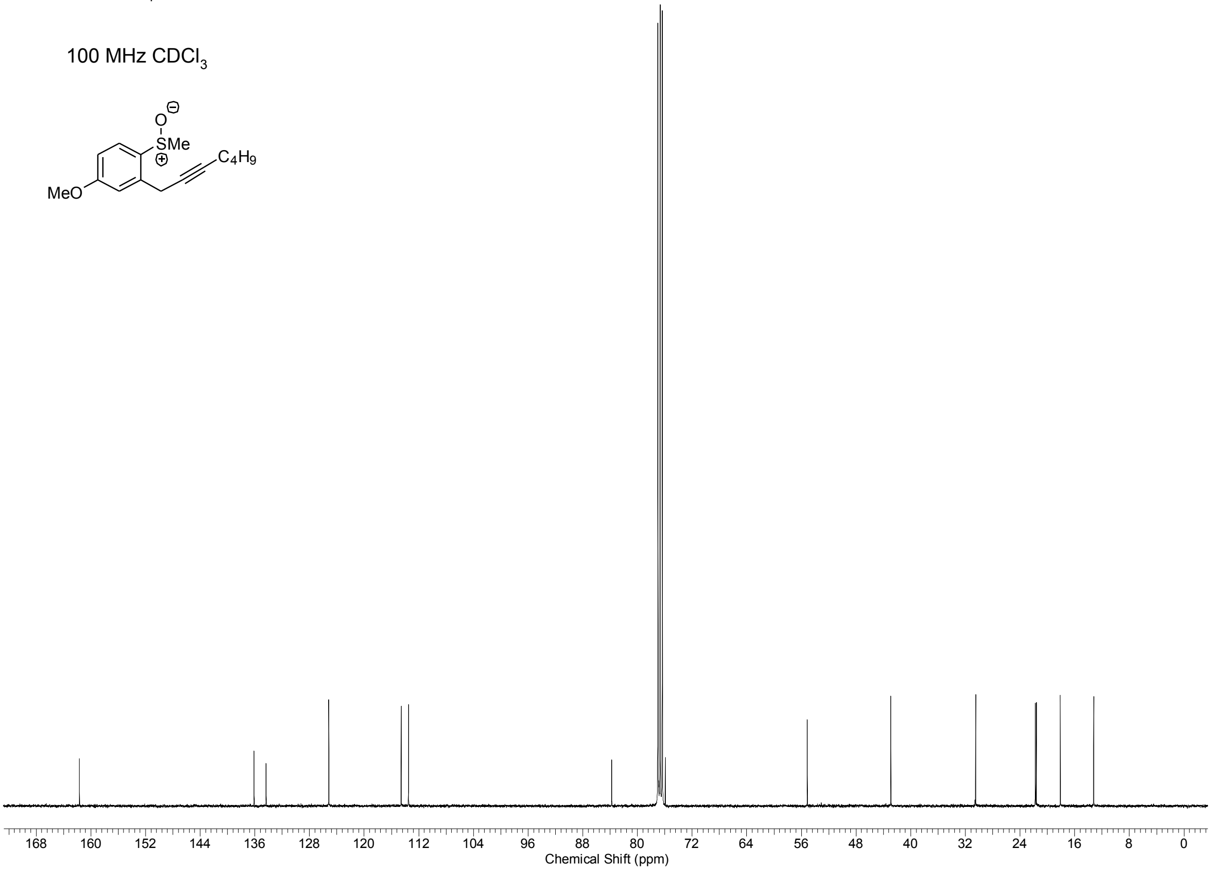

## (2-(3-Cyclohexylprop-2-yn-1-yl)-6-(hept-2-yn-1-yl)-4-methoxyphenyl)(methyl)sulfane 3ba

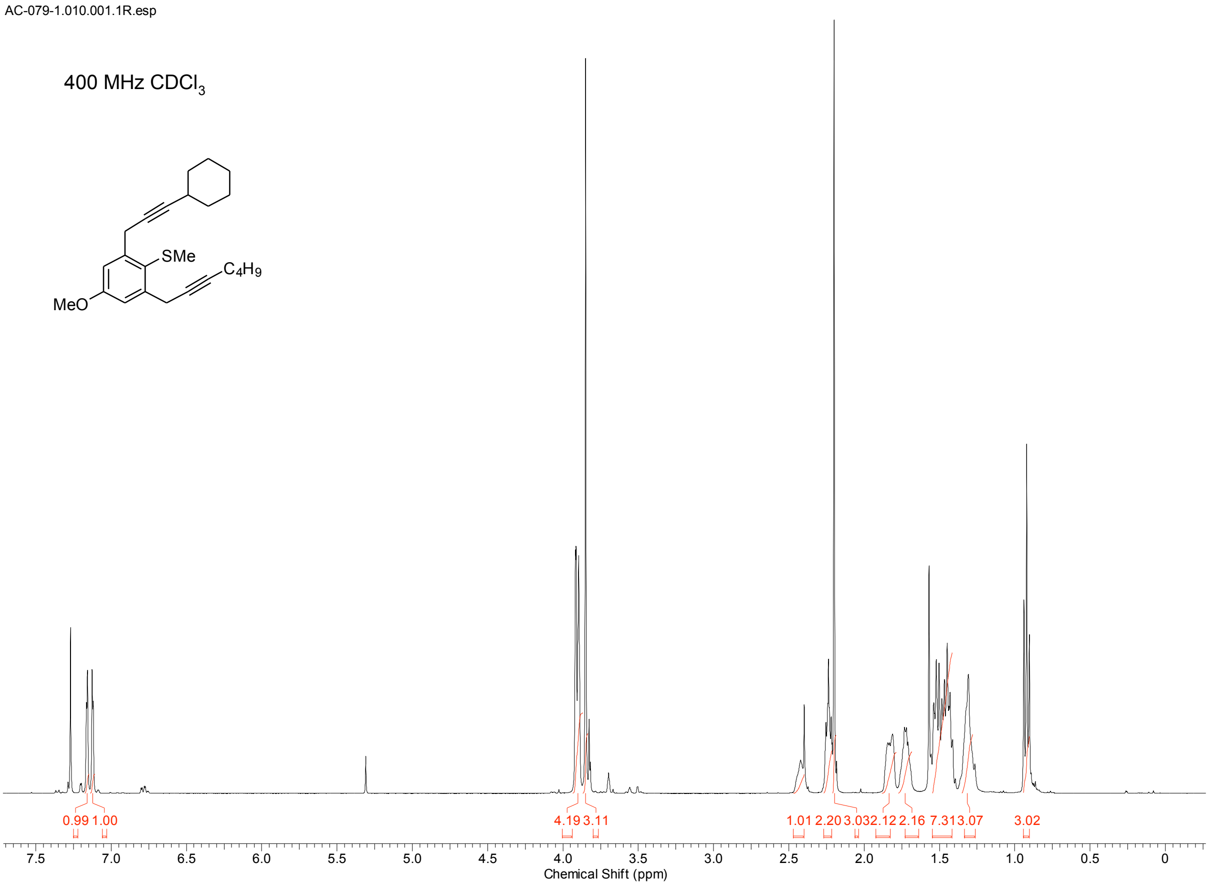


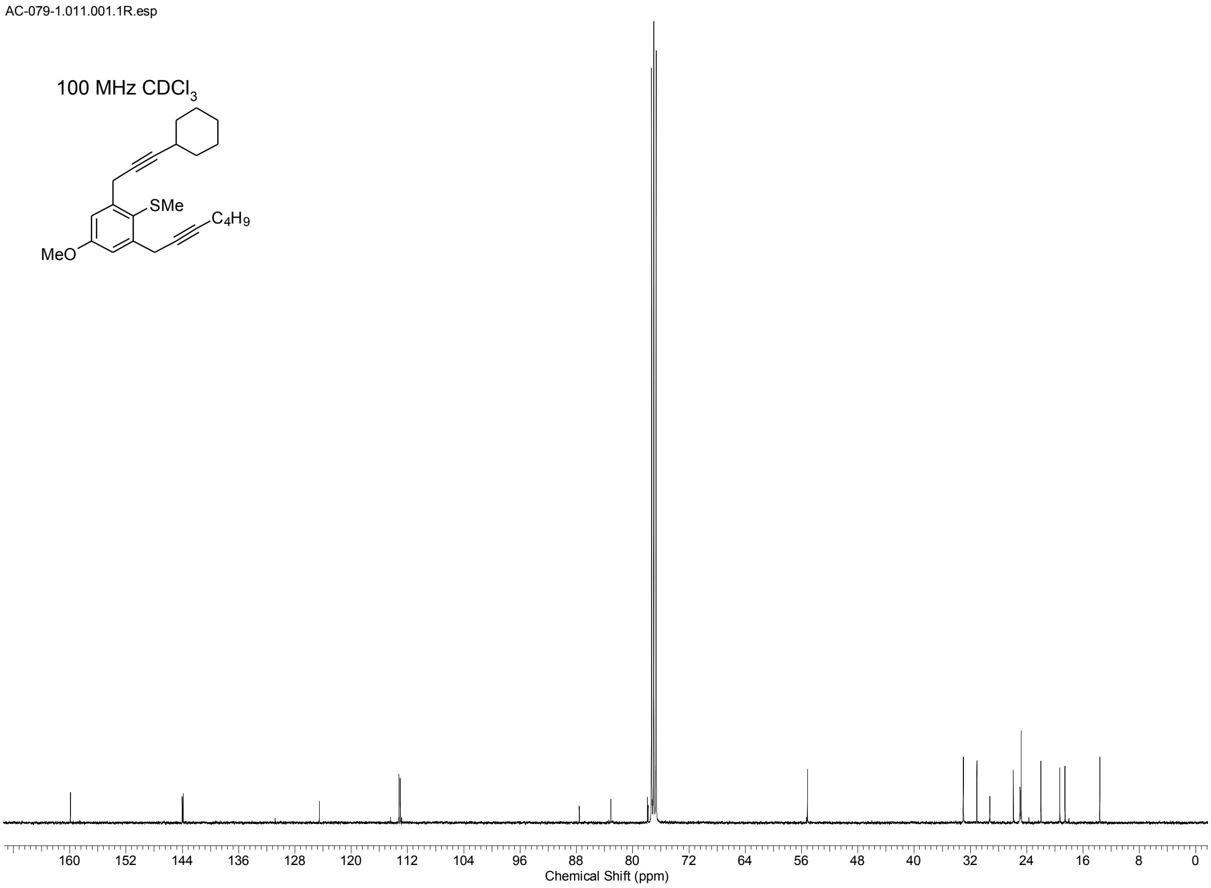

# 1-(3-methylbenzo[b]thiophen-2-yl)propan-1-one 7c

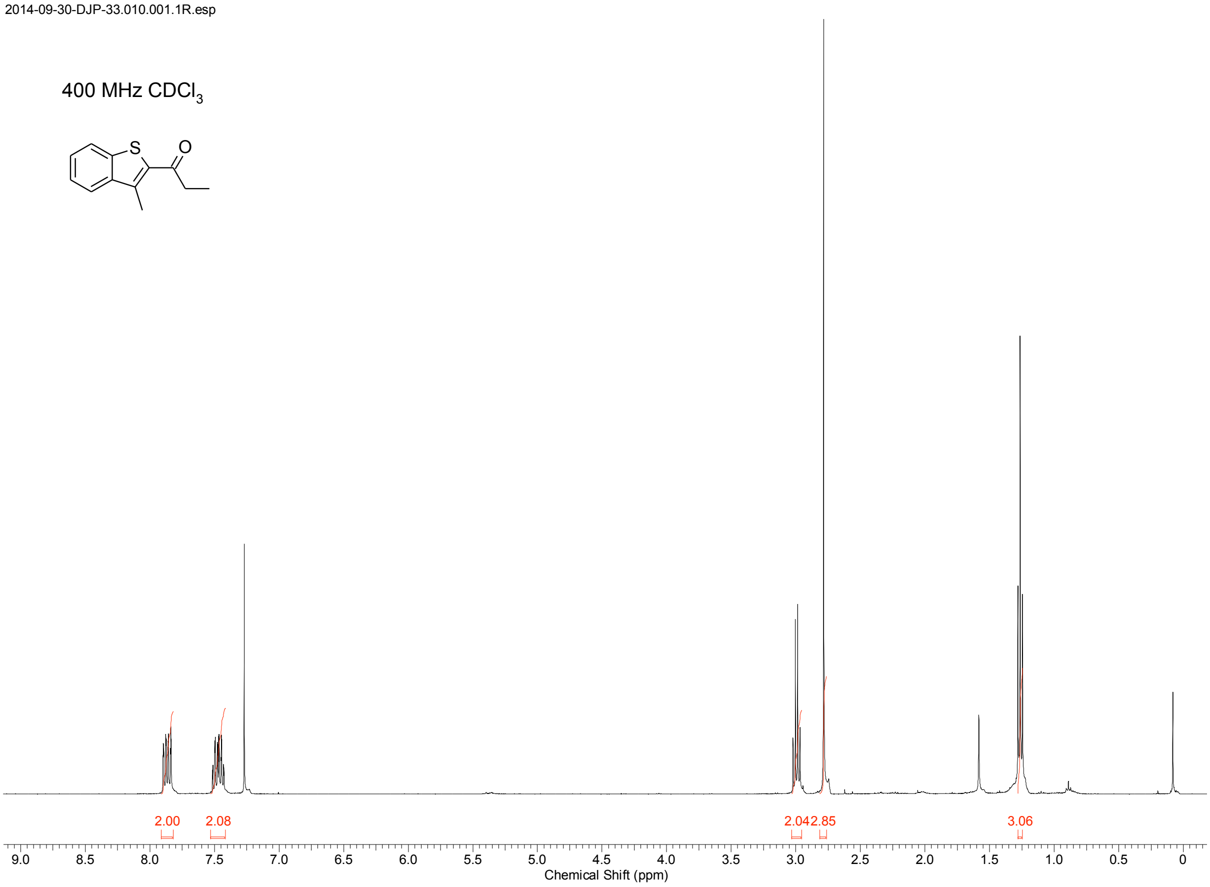


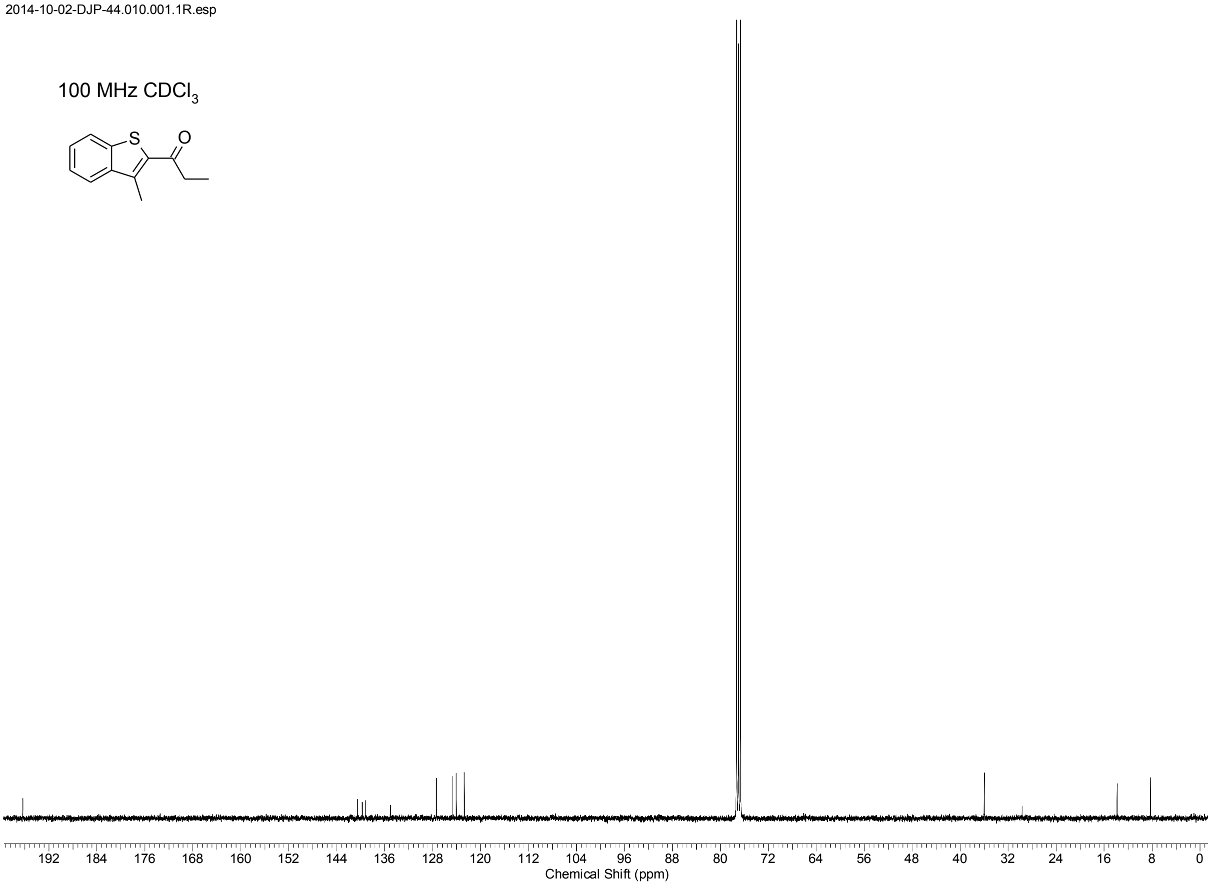

Supplement: Supplementary file 1 — miscellaneous_information [file chem0021-7428-sd1.pdf]
